# Identification of metabolites associated with prostate cancer risk: a nested case-control study with long follow-up in the Northern Sweden Health and Disease Study

**DOI:** 10.1186/s12916-020-01655-1

**Published:** 2020-07-23

**Authors:** Hanna E. Röhnisch, Cecilie Kyrø, Anja Olsen, Elin Thysell, Göran Hallmans, Ali A. Moazzami

**Affiliations:** 1 Department of Molecular Sciences, Swedish University of Agricultural Sciences, Box 7015, 75007 Uppsala, Sweden; 2 Unit of Diet, Genes and Environment, Danish Cancer Society Research Center, Copenhagen, Denmark; 3 Department of Medical Biosciences, Umeå University, Umeå, Sweden; 4 Department of Public Health and Clinical Medicine, Nutritional Research, Umeå University, Umeå, Sweden

**Keywords:** Prostate cancer, Metabolomics, Nested case-control study, Nuclear magnetic resonance spectroscopy, Mass spectrometry, Risk biomarkers

## Abstract

**Background:**

Prostate cancer is the second most frequently diagnosed cancer in men. Metabolomics can potentially provide new insights into the aetiology of prostate cancer by identifying new metabolic risk factors. This study investigated the prospective association between plasma metabolite concentrations and prostate cancer risk, both overall and by stratifying for disease aggressiveness and baseline age.

**Methods:**

In a case-control study nested in the Northern Sweden Health and Disease Study, pre-diagnostic concentrations of 148 plasma metabolites were determined using targeted mass spectrometry- and nuclear magnetic resonance-based metabolomics in 777 prostate cancer cases (follow-up ≥ 5 years) and 777 matched controls. Associations between prostate cancer risk and metabolite concentrations were investigated using conditional logistic regression conditioned on matching factors (body mass index, age and sample storage time). Corrections for multiple testing were performed using false discovery rate (20%) and Bonferroni. Metabolomics analyses generated new hypotheses, which were investigated by leveraging *food frequency questionnaires* (FFQs) and oral glucose tolerance tests performed at baseline.

**Results:**

After correcting for multiple testing, two lysophosphatidylcholines (LPCs) were positively associated with risk of overall prostate cancer (all ages and in older subjects). The strongest association was for LPC C17:0 in older subjects (OR = 2.08; 95% CI 1.45–2.98; *p* < 0.0001, significant also after the Bonferroni correction). Observed associations with risk of overall prostate cancer in younger subjects were positive for glycine and inverse for pyruvate. For aggressive prostate cancer, there were positive associations with six glycerophospholipids (LPC C17:0, LPC C20:3, LPC C20:4, PC ae C38:3, PC ae C38:4 and PC ae C40:2), while there was an inverse association with acylcarnitine C18:2. Moreover, plasma LPC C17:0 concentrations positively correlated with estimated dietary intake of fatty acid C17:0 from the FFQs. The associations between glycerophospholipids and prostate cancer were stronger in case-controls with normal glucose tolerance.

**Conclusions:**

Several glycerophospholipids were positively associated with risk of overall and aggressive prostate cancer. The strongest association was observed for LPC C17:0. The associations between glycerophospholipids and prostate cancer risk were stronger in case-controls with normal glucose tolerance, suggesting a link between the glucose metabolism status and risk of prostate cancer.

## Background

Prostate cancer is the second most common cancer among men worldwide [1]. The rapid increase in incidence during recent decades may owe partly to extrinsic factors (diet and lifestyle) [2, 3]. Insulin-like growth factor I (IGF-I), overweight and obesity have been consistently identified as risk factors for prostate cancer [4, 5]. In addition, associations with risk of prostate cancer have been observed positively with intake of dairy products and calcium, and inversely with plasma concentrations of alpha-tocopherol and selenium; however, the evidences are limited [5].

Using metabolomics to measure a large number of low molecular weight compounds (metabolites) in biofluids, reflecting the final read-out of gene-environment interactions [6–8], may help to identify novel risk factors for prostate cancer.

To the best of our knowledge, seven previous studies, conducted within three cohorts, have investigated the association between pre-diagnostic levels of plasma and serum metabolites and the risk of prostate cancer incidence [9–15]. The results of these studies differ somewhat as regards metabolites associated with disease risk. The differences may be partly explained by the use of different metabolomics methodologies, varying characteristics of the study populations and dissimilarities in the experimental designs (e.g. sample size, fasting status, follow-up time, matching factors and disease subtype categorisation). Therefore, the identification of metabolites associated with risk of prostate cancer warrants further investigation.

By carefully monitoring individual metabolites, we and others have previously shown that the concentrations of circulating metabolites are subjected to changes in response to a meal [16–18]. For example, concentrations of phospholipids, amino acids and their breakdown products, glycolytic products, acylcarnitines and ketone bodies vary by up to 80% 0–3 h after a meal [16] and by up to 100% 0–8 h after a meal [17]. The variation in concentration of metabolites because of varying time since last meal is not related to the estimation of risk and may therefore hinder identification of metabolites associated with disease risk. This variation can be reduced by including only case-control sets for which plasma samples are collected after overnight fasting. Moreover, prostate cancer can pass through a period of latency during which the subclinical disease may cause metabolic changes [13]. These changes are not directly related to the aetiology of the disease and should therefore be avoided. The possibility of such changes can be reduced by including only cases with a long minimum follow-up time (e.g. time between sample collection and diagnosis ≥ 5 years). However, the two largest studies previously performed did not include only (1) case-control sets with fasting samples or (2) cases with a long follow-up time in all subgroups analysed [13, 15]. In addition, baseline age-specific association with risk of prostate cancer has been shown for IGF-I [19]. However, to our knowledge, it has not been investigated whether the association between metabolites and prostate cancer risk varies with baseline age [9–15].

The aim of this study was to investigate the prospective association between metabolite concentrations and risk of prostate cancer in a case-control study nested within the Northern Sweden Health and Disease Study (NSHDS) cohort. Subgroup analyses were performed after stratification by disease subtype (non-aggressive, aggressive) and baseline age (40–50, 60 years). In order to reduce the variation in concentrations of metabolites that are not directly related to estimation of disease risk, i.e. the variation caused by eating a meal or by subclinical prostate cancer, we only included case-control sets with fasting samples and cases with a minimum follow-up time of at least 5 years (≥ 5 years). In addition, for the first time, we applied combined targeted mass spectrometry (MS) and nuclear magnetic resonance (NMR) spectroscopy-based metabolomics approach to quantify a larger number of metabolites. Metabolomics analyses led to the generation of new hypotheses, which were corroborated for the first time by leveraging *food frequency questionnaires* (FFQs) and a complementary oral glucose tolerance test (OGTT) performed at baseline.

## Methods

### The Northern Sweden Health and Disease Study

The NSHDS is a population-based cohort that started in 1985 [20]. In brief, residents in the Swedish province of Västerbotten were invited to a health assessment at 40, 50 and/or 60 years of age. In the assessment, each participant underwent a health examination, including measurement of height, weight and blood pressure. In addition, an OGTT was performed with a 75-g oral glucose load, according to the World Health Organization (WHO) standards, and all participants were asked to complete a validated FFQ. The FFQ covered various food items from which dietary intake of different nutrients, e.g. different fatty acids (FAs), can be calculated [21]. Nutrient intake was calculated by multiplying the daily intake of different food items calculated from the FFQ by the nutrient content of each food item extracted from a food composition database at the Swedish National Food Administration [22]. Finally, participants were asked to donate blood for future research purposes. The blood sample from each participant was drawn after overnight fasting; separated into buffy coat, erythrocytes and (heparin) plasma aliquots; and then stored at − 80 °C (within 2 h of collection) at the Medicinal Biobank, Umeå University. All participants gave written informed consent to participate in the study. The study was approved by the Research Ethics Committee of Umeå University Hospital and the Regional Ethics Committee in Uppsala (no. 2013-124).

### Study design

A nested case-control study on prostate cancer was designed within the NSHDS cohort. Among the 38,467 men who participated in the health survey between 1985 and 2007, 1664 were diagnosed with prostate cancer based on national registries to which reporting is mandated by law (e.g. patients’ registry, cancer registry and cause of death registry) during the follow-up period until 2012. A total of 777 cases were selected for the present study after applying some inclusion/exclusion criteria. The criteria for inclusion were as follows: (1) overnight fasting, (2) no previous cancer incidence before prostate cancer diagnosis, (3) ≥ 5 years between the time of sample collection (baseline) and prostate cancer diagnosis and (4) no type 2 diabetes (T2D) diagnosed at baseline (declared during the health assessment).

Each selected case was classified as having either an aggressive or non-aggressive subtype of the disease, based on the International Union Against Cancer classification system [23, 24]. Aggressive prostate cancer was defined as poorly differentiated tumour (Gleason’s score 8–10 or grade 3 in the three-level WHO grading system, with grade 3 indicating the lowest level of differentiation [19, 25, 26]), non-localised tumour (T3–4), lymph node metastasis (N1), bone metastasis (M1), serum prostate-specific antigen (PSA) level above 50 ng/mL at diagnosis or fatal prostate cancer by March 2007 (irrespective of tumour characteristics at diagnosis). Cases not classified as having an aggressive form of the disease were included in the group of non-aggressive cases.

One control was selected for each prostate cancer case among men who were alive, free of any cancer history at the time of diagnosis of the corresponding case and had a plasma sample collected after overnight fasting. Cases and controls were matched according to age, body mass index (BMI) and duration of sample storage in the freezer. The windows used for matching factors to select a control for each case from NSHDS cohort were as follows: age ± 210 days, BMI ± 0.8 kg/m^2^ and sample storage in freezer ± 220 days. None of the selected controls had a T2D diagnosis at baseline.

During selection of cases and controls from NSHDS for the present study, we set a criteria to exclude cases and controls who declared having T2D in the health assessment at the time of sample collection (baseline). However, the metabolomics analysis led to new hypotheses which were investigated after retrieving the data from an OGTT performed at baseline. Using the OGTT data, we identified some new cases and controls with T2D according to the WHO criteria [27] at baseline, but whose T2D had not been declared in the health assessment.

In NSHDS, at enrolment, the participants were invited at even decades, i.e. 40, 50 and 60 years of age. Therefore, the present study included participants who were 40 (*n* = 45 sets), 50 (*n* = 288 sets) and 60 (*n* = 444 sets) years old. This allowed the stratification of case-control sets into age-specific subgroups [19, 24]. Because of the lower prevalence of prostate cancer in younger participants, we included 40- and 50-year-olds in the same subgroup (younger, 40–50 years, *n* = 333 sets), while 60-year-olds were included in another subgroup (older, 60 years, *n* = 444 sets).

### Sample analysis

#### Targeted MS- and NMR-based metabolomics

Plasma samples were analysed using both targeted MS- and NMR-based metabolomics (Additional file 1). The targeted MS-based metabolomics was performed using the AbsoluteIDQ p180 assay (BIOCRATES, Innsbruck, Austria), quantifying 188 metabolites [28]. The targeted NMR-based metabolomics was performed using an automated quantification algorithm (AQuA), which was specifically developed in our laboratory for large epidemiological studies quantifying 67 metabolites from the NMR spectra of human plasma [29].

#### Metabolite inclusions and exclusions

Metabolites with occurrence below 50% in samples from the nested cohort or an analytical coefficient of variation (CV) above 15% in quality control (QC) samples were excluded from the statistical analyses. The quality control criteria were fulfilled by 103 (out of 188) metabolites in the targeted MS analysis and by 45 (out of 67) metabolites in the targeted NMR analysis, which were used in statistical analysis (Additional file 2).

### Statistical analyses

#### Identification of metabolites associated with risk of prostate cancer

The association between each individual metabolite and the risk of prostate cancer was assessed using a conditional logistic regression model conditioned on matching factors (BMI, age and sample storage time). The metabolite concentrations were log_2_-transformed, and thus, linear estimates were per doubling. This derived the crude odds ratio (OR_crude_), the 95% confidence interval (CI) and the corresponding *p* value (*p*_crude_) for each association. Analyses were repeated after stratification by disease subtype (non-aggressive, aggressive) and baseline age (40–50, 60 years). The conditional logistic regression analyses were conducted using the PHREG procedure in SAS (version 9.3, SAS Institute Inc., Cary, NC), with the case-control sets as strata.

Correction for multiple testing was performed using two approaches of varying stringency/conservancy, in consistence with previous studies (low stringency: [9]; high stringency: [12]). For the low stringency approach, false discovery rate (FDR) correction (significance level at 20%) was performed using the Q-value package [30] in RStudio (version 3.0.3, R Foundation for Statistical Computing Platform, Vienna, Austria). This approach was employed for holistic interpretation of results and hypothesis generation. For the high stringency approach, the Bonferroni correction was employed, using the number of metabolites as number of variables (*α* = 0.05/148).

For statistically significant metabolites (FDR 20%), each conditional logistic regression model was further adjusted for BMI and exact age (continuously) in one model and additionally for alcohol intake (< 10, 10–19, 20–39, ≥ 40 g/day) and smoking (no, past, current, unknown) in another model [13]. The estimated risk for each metabolite by the adjusted models did not differ from the unadjusted model by more than 10%, and therefore, only results from the unadjusted model are presented.

Possible differential associations by age or disease type were investigated for each of the metabolites that were statistically significant after correction for multiple testing (FDR 20%). This was done by associating individual plasma metabolites with prostate cancer risk allowing for different associations for each of the two categories for disease type and baseline age, respectively (for disease type: binary, non-aggressive, aggressive; for age: binary, 40–50, 60 years). Each comparison was made using a Wald test for the hypothesis of equal regression coefficients. Log-transformed metabolite data were used.

For statistically significant metabolites (FDR 20%), each conditional logistic regression model was also repeated for subgroups by follow-up time (≤ 10, > 10 years). Possible differential associations by follow-up time were performed as described above.

Categorical logistic regression analysis was also performed for each metabolite (FDR 20%) based on quartile values. Each subject was assigned to a quartile based on cut-off values derived from the distribution of concentrations in the control group. The first quartile was used as a referent level.

#### Correlation analyses

Spearman’s correlation coefficient was used to assess the correlation between metabolite concentrations (lysophosphatidylcholines, LPCs) and baseline characteristics (glucose values from the OGTT and estimated daily intake of different FAs from the FFQ). Correlation analysis was performed using the CORR procedure in SAS (version 9. 3, SAS Institute Inc., Cary, NC).

#### Status of glucose metabolism at baseline and risk of prostate cancer

Metabolomics findings led to a hypothesis of a link between baseline glucose metabolism and risk of prostate cancer. The subjects in this study underwent an OGTT at enrolment, so the status of glucose metabolism at baseline, i.e. normal glucose tolerance (NGT), impaired fasting glucose (IFG) and impaired glucose tolerance (IGT) or T2D, could be determined using the WHO criteria [27]. A categorical logistic regression analysis (NGT as the referent) was used to assess the association between the status of glucose metabolism and risk of prostate cancer (overall, and by restricting to aggressive or non-aggressive cases and stratifying by baseline age).

Conditional logistic regression analyses were repeated after excluding case-controls with abnormal glucose metabolism at baseline (i.e. IFG, IGT or T2D) and thereby restricting to case-controls with NGT. These analyses were limited to metabolites found to be associated with prostate cancer risk after correction for multiple testing (FDR 20%). This was performed to examine whether the associations between these metabolites and prostate cancer risk were improved in matched case-controls with NGT.

## Results

### Baseline characteristics

Table 1 presents baseline characteristics (clinical measures, blood parameters, status of glucose metabolism based on OGTT results and estimated daily intakes of different FAs reported in the FFQ) for the respective case-control sets included in the present study. Values are presented for the entire study population and for the subgroups of younger (40–50 years) and older (60 years) subjects, respectively.

**Table 1:** Selected baseline characteristics of the study subjects

	**40–60 years (*n*** **= 777:777)**		**40–50 years (*n*** **= 333:333)**		**60 years (*n*** **= 444:444)**	
	**Controls**	**Cases**	**Controls**	**Cases**	**Controls**	**Cases**
**Clinical characteristics**^**a,b**^						
Age (years)^c^	59.8 (40.3–60.3)	59.8 (40.4–60.3)	50.0 (40.1–50.3)	50.0 (40.1–50.4)	60.0 (59.7–60.3)	60.0 (59.5–60.4)
BMI (kg/m^2^)^c^	25.8 (21.8–31.5)	25.7 (21.7–31.4)	25.5 (22.0–31.3)	25.5 (22.0–31.3)	26.1 (21.6–31.6)	26.0 (21.4–31.5)
Height (cm)	177 (167–187)	177 (167–186)	179 (167–189)	178 (168–187)	176 (167–186)	176 (166–186)
Weight (kg)	81 (66–100)	81 (66–101)	81 (67–101)	81 (66–101)	82 (65–100)	81 (65–101)
SBP (mmHg)	130 (110–165)	130 (110–165)	125 (106–155)	125 (105–153)	137 (110–170)	138 (110–170)
DBP (mmHg)	82 (65–100)	83 (65–100)	80 (64–98)	80 (65–99)	85 (70–100)	85 (70–100)
**Dietary intake (FFQ)**^**a,b**^						
Energy (kcal/day)	1952 (1116–3183)	1974 (1141–3091)	1910 (1126–3032)	1985 (1155–3140)	1972 (1116–3262)	1940 (1129–3082)
Total fat (g/day)	74.1 (39.4–133)	75.2 (40.3–129)	75.4 (42.7–133)	77.7 (37.6–129)	73.2 (36.4–135)	72.6 (40.5–131)
FA C14:0 (g/day)	3.34 (1.34–7.28)	3.30 (1.51–7.18)	3.64 (1.59–7.30)	3.60 (1.61–7.48)	3.08 (1.25–7.22)	3.14 (1.44–6.58)
FA C16:0 (g/day)	14.9 (7.39–27.9)	15.1 (7.89–28.1)	16.3 (8.54–28.3)	16.8 (8.11–28.5)	14.1 (7.02–27.2)	13.8 (7.73–27.4)
FA C17:0 (g/day)	0.13 (0.03–0.33)	0.13 (0.04–0.34)	0.15 (0.04–0.33)	0.14 (0.04–0.35)	0.12 (0.03–0.34)	0.13 (0.04–0.32)
FA C18:2 (g/day)	7.23 (3.62–15.0)	7.24 (3.64–14.8)	7.74 (4.28–17.2)	7.99 (3.82–15.7)	6.90 (3.39–13.4)	6.61 (3.51–14.1)
FA C20:4 (g/day)	0.07 (0.03–0.17)	0.07 (0.03–0.17)	0.08 (0.03–0.18)	0.08 (0.04–0.18)	0.07 (0.03–0.15)	0.07 (0.03–0.16)
**Blood parameters (OGTT; mmol/L)**^**a,b**^						
Glucose (0 h)	5.5 (4.6–6.6)	5.5 (4.5–6.7)	5.4 (4.6–6.4)	5.4 (4.4–6.6)	5.5 (4.7–6.7)	5.5 (4.5–6.8)
Glucose (2 h)	6.5 (4.0–9.5)	6.4 (4.1–9.3)	6.3 (3.9–8.6)	6.0 (3.8–8.7)	6.7 (4.1–10.3)	6.7 (4.4–9.8)
**Status of glucose metabolism**^**a,b**^						
NGT	541 (70)	580 (75)	255 (77)	272 (82)	286 (65)	308 (69)
IFG	67 (9)	70 (9)	26 (8)	27 (8)	41 (9)	43 (10)
IGT	138 (18)	93 (12)	46 (14)	23 (7)	92 (21)	70 (16)
T2D	27 (3)	31 (4)	5 (1)	10 (3)	22 (5)	21 (5)

*Abbreviations*: *SBP* systolic blood pressure, *DBP* diastolic blood pressure

^a^Data on each baseline characteristic were available for > 95% of the case-controls

^b^Continuous variables are listed as median (5th–95th percentile), and categorical variables are listed as *N* (%)

^c^Matching factors: age and BMI (and sample storage time)

### Case characteristics

Table 2 displays the characteristics of prostate cancer cases for the entire study population and for the subgroups of younger and older subjects. The median age at prostate cancer diagnosis was 67 years (range 45.9–80.2 years), and the median time between sample collection (baseline) and prostate cancer diagnosis was 10.3 years (range 5.0–19.9 years). For younger cases, the follow-up time was 11.8 (5.1–19.5) years, while for older cases, it was 9.5 (5.0–19.9) years. Overall, 22% of cases were classified as having an aggressive form of the disease (Table 2).

**Table 2:** Case characteristics at the time of diagnosis

	***N*(%)**		
	**40–60 years (*n* = 777)**	**40–50 years (*n* = 333)**	**60 years (*n* = 444)**
**Follow-up time**			
≥ 5, < 10 years	368 (47)	118 (35)	250 (56)
≥ 10 years	409 (53)	215 (65)	194 (44)
**Age at diagnosis**			
< 65 years	282 (36)	276 (83)	6 (1)
≥ 65 years	495 (64)	57 (17)	438 (99)
**Tumour differentiation**^**a**^			
Poorly	114 (15)	33 (10)	81 (18)
Highly/intermediately	656 (84)	299 (90)	357 (81)
Missing	7 (1)	1 (0)	6 (1)
**Primary tumour**			
Non-assessed (TX)	15 (2)	9 (3)	6 (1)
Non-palpable (T1)	433 (56)	209 (63)	224 (50)
Localised (T2)	248 (32)	94 (28)	154 (35)
Non-localised (T3–T4)	74 (9)	18 (5)	56 (13)
Missing	7 (1)	3 (1)	4 (1)
**Serum PSA**			
≤ 50 ng/mL	703 (90)	314 (94)	389 (88)
> 50 ng/mL	67 (9)	18 (6)	49 (11)
Missing	7 (1)	1 (0)	6 (1)
**Disease aggressiveness**^**b**^			
Non-aggressive	608 (78)	289 (87)	319 (72)
Aggressive	169 (22)	44 (13)	125 (28)

^a^Poorly differentiated tumour: Gleason’s sum score 8–10 or grade 3 (in the three-level WHO grading system). Highly/intermediately differentiated tumour: Gleason’s sum score ≤ 7 or grade 1–2 (in the three-level WHO grading system)

^b^Aggressive case subjects: poorly differentiated (Gleason sum’s score 8–10 or grade 3), non-localised tumour (i.e. primary tumour stage T3–4), lymph node metastasis (N1), bone metastasis (M1), serum PSA concentration > 50 ng/mL or fatal prostate cancer by March 2007

### Identification of metabolites associated with risk of prostate cancer

#### Metabolites with nominal *p* value < 0.05

**Fig. 1:**
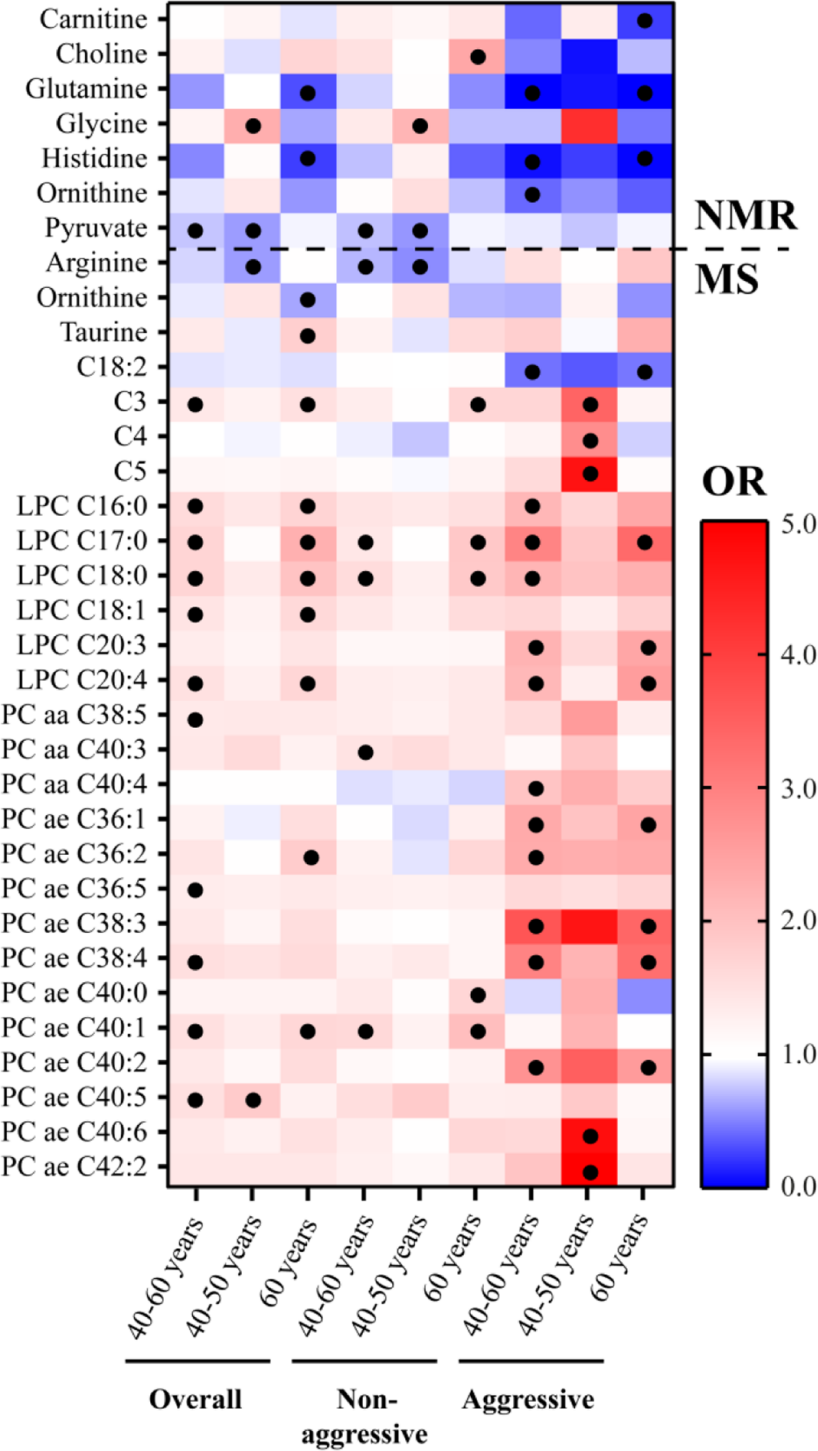
Odds ratio (OR) values for the association between individual metabolites (LPC, lysophosphatidylcholine; PC, phosphatidylcholine) and prostate cancer risk in different subgroups. OR > 1, red; OR < 1, blue; nominal *p* value < 0.05, filled black circles. Top: metabolites measured with nuclear magnetic resonance (NMR). Bottom: metabolites measured with mass spectrometry (MS). Subgroups: overall prostate cancer (40–60 years, 777 matched case-control sets; 40–50 years, 333 sets; 60 years, 444 sets), non-aggressive prostate cancer (40–60 years, 608 sets; 40–50 years, 289 sets; 60 years, 319 sets) and aggressive prostate cancer (40–60 years, 169 sets; 40–50 years, 44 sets; 60 years, 125 sets)

The metabolites associated with prostate cancer risk before and after stratification by disease subtype and baseline age (nominal *p* value < 0.05; Additional file 3) are shown in Fig. 1. Several glycerophospholipids (LPCs and phosphatidylcholines (PCs)) and glycine were frequently associated with risk of prostate cancer (OR > 1). The associations observed for glycerophospholipids were typically stronger for risk of aggressive disease and in older subjects. The association observed for glycine was generally stronger for risk of overall and non-aggressive disease in younger subjects. Pyruvate, arginine, ornithine and acylcarnitine C18:2 were frequently associated with risk of prostate cancer (OR < 1).

#### Metabolites significant after correction for multiple testing

Metabolites with a significant association with risk of prostate cancer after correction for multiple testing (FDR 20% and Bonferroni) are shown in Table 3 (Additional file 4). Higher plasma concentrations of LPC C17:0 and LPC C18:0 were associated with higher risk of overall prostate cancer, and each respective association was stronger in older subjects. Importantly, the association between LPC C17:0 and overall prostate cancer risk in older subjects was significant after the Bonferroni correction. Higher plasma concentration of glycine and pyruvate was associated with higher and lower risk of overall prostate cancer in younger subjects, respectively. Higher concentrations of six glycerophospholipids (LPC C17:0, LPC C20:3, LPC C20:4, PC ae C38:3, PC ae C38:4 and PC ae C40:2) were associated with higher risk of aggressive prostate cancer, while higher concentrations of acylcarnitine C18:2 were associated with lower risk of aggressive prostate cancer. The association observed for LPC C17:0 was stronger in older subjects, where individuals in the top quartile had 3.9-fold higher odds of developing aggressive disease (Table 3). None of the associations with non-aggressive prostate cancer risk (Fig. 1) was significant after correction for multiple testing. There was heterogeneity between the two age categories (40–50, 60 years) for LPC C17:0, LPC C18:0 and glycine (Additional file 5). Only PC ae C38:0 showed heterogeneity between disease subtype categories (aggressive, non-aggressive; Additional file 5). There was no heterogeneity between follow-up time categories (5–10, > 10 years; Additional file 6).

**Table 3:** Relationship between baseline metabolite concentration and risk of prostate cancer after correction for multiple testing

**Age**	**Metabolite**	**Linear estimates**^**a**^		**OR (95% CI) for quartiles**^**b**^			
		OR (95% CI)	***p***_**crude**_	Q1	**Q2**	**Q3**	**Q4**
**Overall prostate cancer**							
40–60 years	LPC C17:0	1.59 (1.21–2.08)	0.0007	1.00 (referent)	1.44 (1.07–1.96)*	1.43 (1.06–1.94)*	1.63 (1.20–2.21)*
	LPC C18:0	1.59 (1.17–2.18)	0.0034	1.00 (referent)	1.08 (0.81–1.45)	1.32 (1.00–1.75)	1.36 (1.02–1.81)*
40–50 years	Glycine	2.11 (1.21–3.69)	0.0084	1.00 (referent)	1.13 (0.70–1.83)	1.12 (0.70–1.81)	1.75 (1.09–2.82)*
	Pyruvate	0.65 (0.48–0.89)	0.0066	1.00 (referent)	0.62 (0.40–0.97)*	1.04 (0.69–1.57)	0.46 (0.29–0.74)*
60 years	LPC C17:0	2.08 (1.45–2.98)	< 0.0001*	1.00 (referent)	1.97 (1.29–3.01)*	2.10 (1.37–3.24)*	2.56 (1.67–3.93)*
	LPC C18:0	1.83 (1.22–2.75)	0.0037	1.00 (referent)	0.95 (0.64–1.39)	1.30 (0.89–1.90)	1.54 (1.07–2.22)*
**Aggressive prostate cancer**							
40–60 years	LPC C17:0	2.67 (1.48–4.83)	0.0011	1.00 (referent)	1.64 (0.80–3.38)	2.69 (1.29–5.60)*	3.35 (1.66–6.76)*
	PC ae C38:3	3.29 (1.50–7.24)	0.0030	1.00 (referent)	1.54 (0.77–3.10)	1.98 (0.96–4.12)	2.52 (1.28–4.97)*
	PC ae C38:4	2.69 (1.25–5.76)	0.0110	1.00 (referent)	1.32 (0.68–2.56)	1.70 (0.85–3.39)	2.24 (1.17–4.29)*
	LPC C20:4	1.99 (1.15–3.44)	0.0142	1.00 (referent)	1.24 (0.59–2.59)	1.98 (0.98–4.02)	2.13 (1.08–4.23)*
	LPC C20:3	2.05 (1.12–3.75)	0.0206	1.00 (referent)	1.41 (0.76–2.61)	1.97 (1.03–3.77)*	1.83 (0.96–3.48)
	PC ae C40:2	2.49 (1.25–4.97)	0.0095	1.00 (referent)	1.02 (0.54–1.94)	1.69 (0.90–3.18)	1.85 (1.00–3.41)*
	C18:2	0.51 (0.29–0.89)	0.0167	1.00 (referent)	0.80 (0.46–1.40)	0.48 (0.25–0.92)*	0.55 (0.30–1.00)*
60 years	LPC C17:0	3.02 (1.52–6.01)	0.0016	1.00 (referent)	1.63 (0.69–3.84)	2.17 (0.92–5.15)	3.90 (1.70–8.99)*

*Abbreviations*: *LPC* lysophosphatidylcholine, *PC* phosphatidylcholine

^a^Listed metabolites were significant after correction for multiple testing (FDR 20%) according to *p* values (*p*_crude_) from conditional logistic regression analyses using log_2_ transformed metabolite data (Additional file 3). Associations significant after the Bonferroni correction (for 148 independent tests; 0.05/148 = 0.000338) in the log_2_-based statistical analyses are indicated by the asterisk symbol (*)

^b^The listed metabolites were also analysed by conditional logistic regression using quartiles (details on the quartile-based statistical analyses are presented in Additional file 4). Associations with *p* value < 0.05 in the quartile-based statistical analyses are indicated by the asterisk symbol (*)

#### Lysophosphatidylcholines

**Fig. 2:**
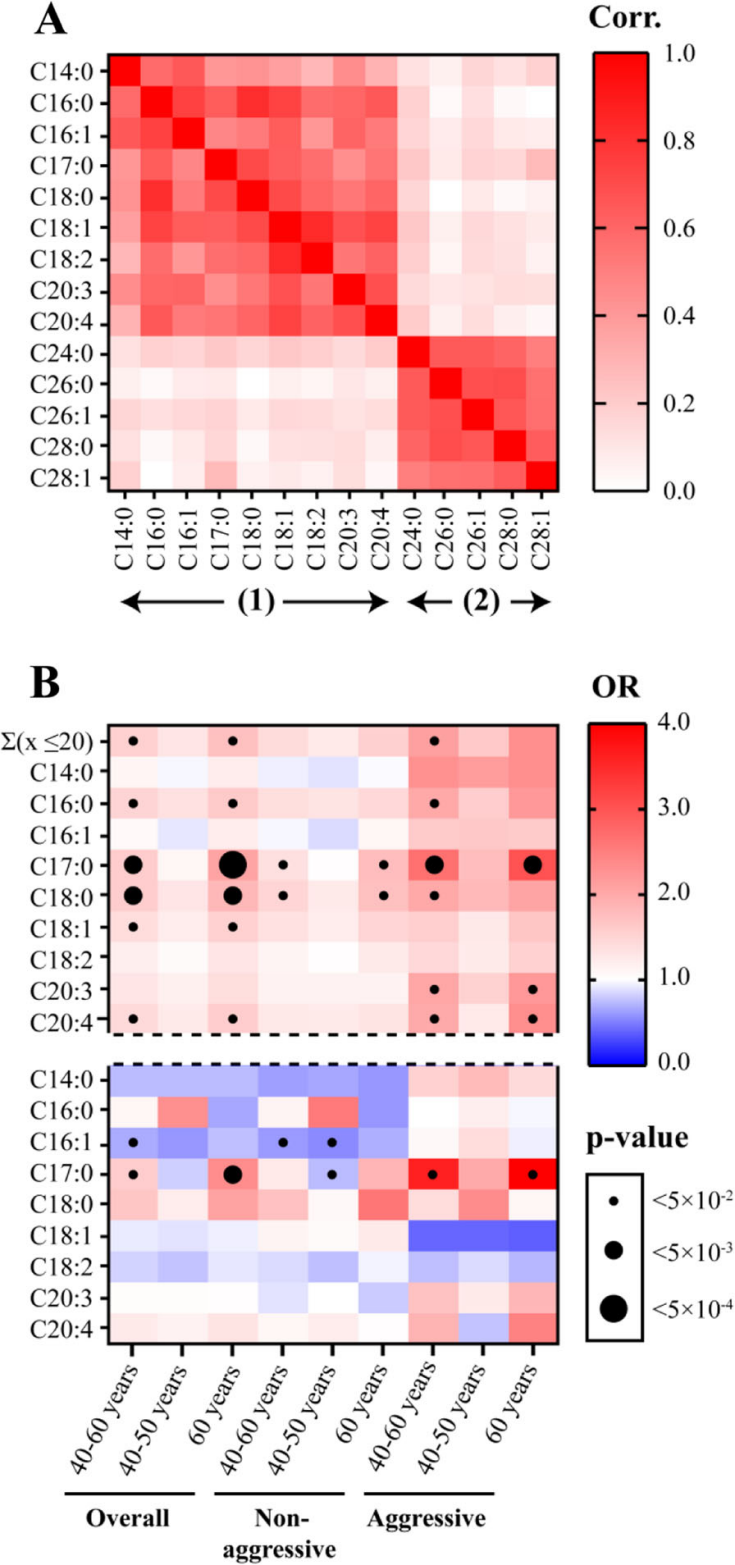
Lysophosphatidylcholines (LPCs) and prostate cancer risk. **a** Spearman’s correlation coefficient (Corr.) matrix for the LPCs Cx:y (*n* = 1554). Cluster (1): *x* ≤ 20, cluster (2): *x* > 20, where strength of each correlation is indicated by the colour intensity (red scale). **b** Odds ratio (OR) values for plasma LPCs (*x* ≤ 20) in the different subgroups before (top panel) and after (bottom panel) adjusting for the total sum of plasma LPCs Σ(*x* ≤ 20). OR > 1, red; OR < 1, blue; level of significance is indicated by the size of the black circles

LPCs attracted particular interest in the analyses, since the majority of metabolites identified belonged to this class of glycerophospholipids (Table 3). Based on Spearman’s correlation coefficients, two clusters of LPCs were identified in the correlation matrix (Fig. 2a). There were significant positive correlations between the LPCs in each cluster. The first cluster included LPCs with ≤ 20 carbons in the FA moiety, and the second cluster included LPCs with > 20 carbons in the FA moiety. The LPCs associated with prostate cancer risk (Table 3) belonged to the first cluster.

The OR values obtained for the individual LPCs in the first cluster (≤ 20 carbons) had a pattern highly similar to that of the total sum of LPCs (Fig. 2b; Additional file 7). After normalisation of each individual LPC to the total sum of LPCs, the associations between LPC C18:0, LPC C20:3 and LPC C20:4 and risk of prostate cancer were no longer significant, while the association between LPC C17:0 and risk of prostate cancer remained significant (Fig. 2b; Additional file 7). This finding clearly distinguished LPC C17:0 from other LPCs in the first cluster.

#### Lysophosphatidylcholines and dietary fatty acids

The correlations between plasma concentration of LPCs and daily dietary intake of the corresponding FA (estimated from the baseline FFQ) were investigated. The strongest correlation was observed between the plasma concentration of LPC C17:0 and estimated daily intake of FA C17:0 (Additional file 8).

#### Lysophosphatidylcholines and glucose metabolism

**Fig. 3:**
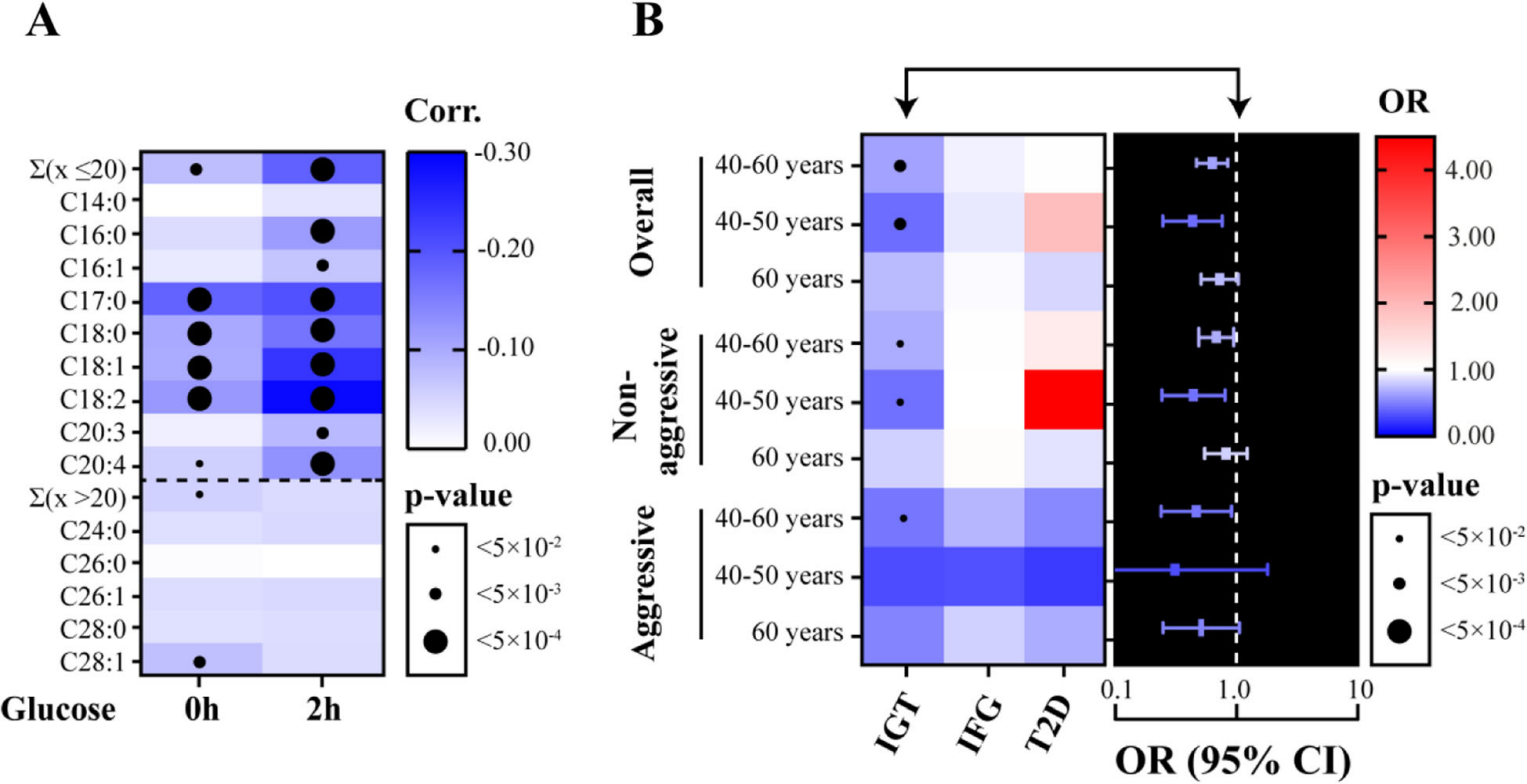
Glucose metabolism and prostate cancer risk. **a** Spearman’s correlation coefficient (Corr.) matrix for lysophosphatidylcholines (LPCs) with glucose values from the oral glucose tolerance test (0 h and 2 h; *n* = 1493); *p* values indicated by the size of the black circles and strength of each correlation by the colour intensity (blue scale). **b** Odds ratio (OR) values for the association between glucose metabolism status at baseline (impaired glucose tolerance (IGT), impaired fasting glucose (IFG) and type 2 diabetes (T2D)) and prostate cancer risk in the different subgroups (left panel). OR (95% CI) for the association between baseline IGT and prostate cancer risk in different subgroups (right panel). OR > 1, red; OR < 1, blue; level of significance indicated by the size of the black circles. Subgroups: overall prostate cancer (40–60 years, 770 matched case-control sets; 40–50 years, 331 sets; 60 years, 439 sets), non-aggressive prostate cancer (40–60 years, 604 sets; 40–50 years, 287 sets; 60 years, 317 sets) and aggressive prostate cancer (40–60 years, 166 sets; 40–50 years, 44 sets; 60 years, 122 sets)

LPCs in the first cluster (≤ 20 carbons) displayed a negative correlation with glucose values from the OGTT, while the correlations between LPCs in the second cluster (with > 20 carbons) and glucose values from the OGTT were generally non-significant (Fig. 3a). The correlations between LPCs in the first cluster and post-load glucose values (2 h) were stronger than the corresponding correlations to pre-load glucose values (0 h; Fig. 3a).

### Status of glucose metabolism and prostate cancer risk

**Fig. 4:**
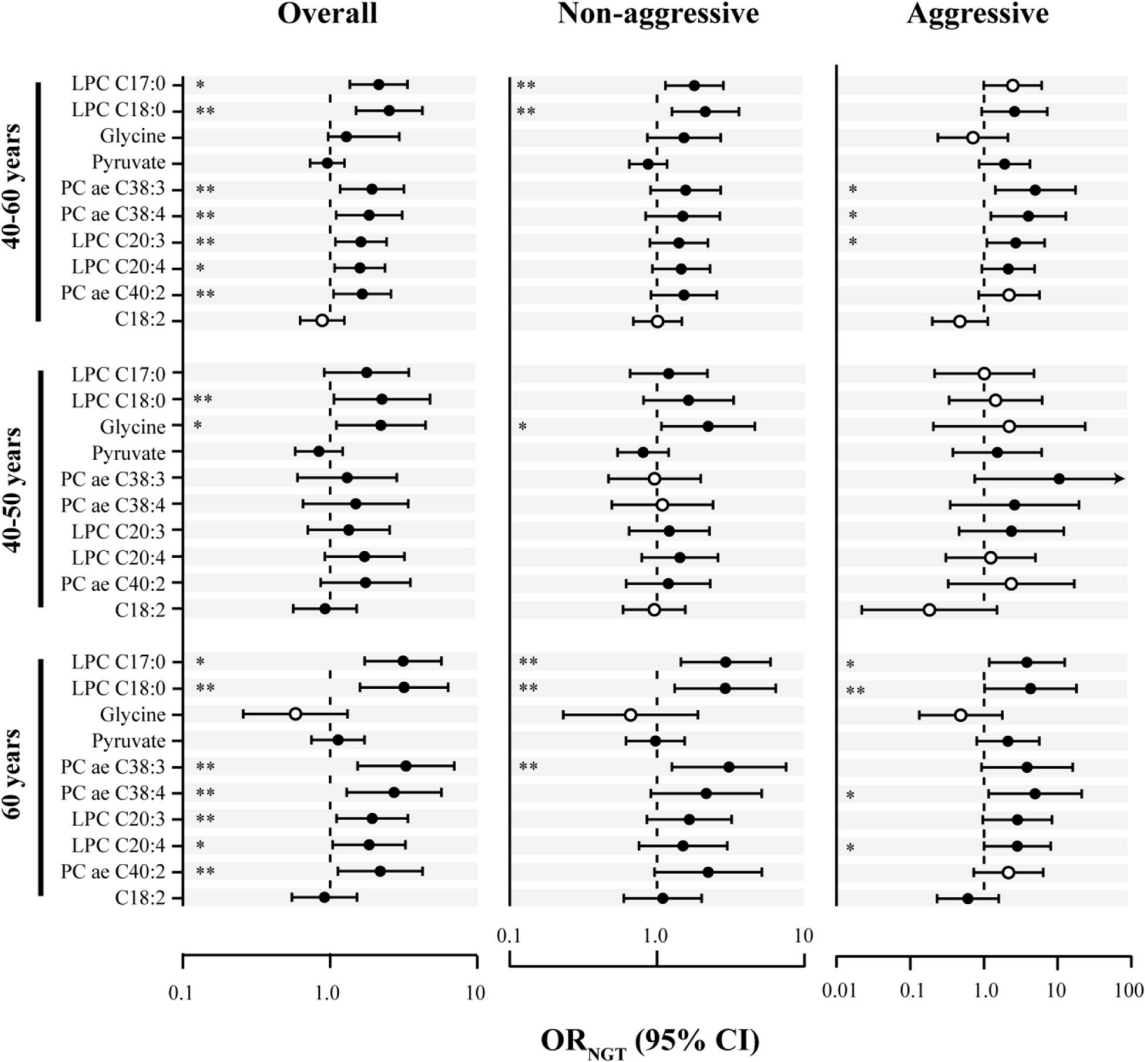
Odds ratio (OR) values for the association between individual metabolites (LPC, lysophosphatidylcholine; PC, phosphatidylcholine) and prostate cancer risk after restriction to case-controls with baseline normal glucose tolerance (NGT). Left: overall prostate cancer in subjects aged 40–60 years (387 matched case-control sets), 40–50 years (198 sets) and 60 years (189 sets). Centre: non-aggressive prostate cancer in subjects aged 40–60 years (311 sets), 40–50 years (173 sets) and 60 years (138 sets). Right: aggressive prostate cancer in subjects aged 40–60 years (76 sets *n* = 152), 40–50 years (25 sets) and 60 years (51 sets). Black filled circles: OR_NGT_ > OR_crude_. Black open circles: OR_NGT_ < OR_crude_. *p_NGT_ < 0.05. **p_NGT_ < 0.05 and *p*_NGT_ < *p*_crude_

LPCs (≤ 20 carbons) were consistently associated with risk of prostate cancer (Fig. 2b) and displayed negative correlations with glucose values from the OGTT (Fig. 3a). Further investigation of whether glucose metabolism status at baseline (i.e. NGT, IFG, IGT or T2D) was associated with prostate cancer risk revealed that IGT was associated with lower risk of prostate cancer (*p* < 0.05; Fig. 3b; Additional file 9). The associations were often stronger in younger subjects than in older subjects (Fig. 3b). For metabolites found to be associated with risk of prostate cancer after correction for multiple testing (Table 3), we investigated whether the associations were altered after limiting the analyses to case-control sets with NGT at baseline. The results showed that associations for some LPCs with ≤ 20 carbons in the FA moiety and for some PCs appeared stronger (*p* < 0.05), but the associations for pyruvate and acylcarnitine C18:2 were not changed (Fig. 4; Additional file 10). The associations for glycine became stronger for overall and non-aggressive prostate cancer in younger subjects (*p* < 0.05).

## Discussion

### Main findings

LPC C17:0 and LPC C18:0 were associated with risk of overall prostate cancer (Table 3; OR > 1), and the association appeared stronger in older subjects (for LPC C17:0, it was still significant after the Bonferroni correction). In younger subjects, glycine and pyruvate were associated with risk of overall prostate cancer (Table 3; glycine: OR > 1; pyruvate: OR < 1). Several glycerophospholipids and acylcarnitine C18:2 were associated with aggressive prostate cancer risk (glycerophospholipids: OR > 1; acylcarnitine C18:2: OR < 1) (Table 3). The strongest association was found in older subjects for LPC C17:0, where individuals in the top quartile had 3.9-fold higher odds of developing aggressive prostate cancer (Table 3).

### Strengths and limitations

The combined targeted MS- and NMR-based metabolomics methodology used in the present study provided absolute metabolite quantities with low analytical variation, and the use of two separate techniques increased the metabolite coverage. The validity of the metabolomics findings was tested using a holistic approach, in which metabolomics-based hypotheses were corroborated by data generated at baseline (FFQ and OGTT). The use of plasma samples collected after overnight fasting reduced the variation in concentrations of metabolites resulting from varying time since the last meal. A long minimum follow-up time (≥ 5 years) reduced the possibility of metabolic alterations due to subclinical prostate cancer at baseline. Each case-control set was matched for BMI and age, to account for the known risk factors for prostate cancer. The study was limited by including individuals from only one European country and by employing only targeted metabolomics, which measures a limited number of metabolites compared with an untargeted approach.

### Other studies

Seven previous studies have investigated the association between metabolite levels and the risk of prostate cancer incidence in prospectively collected human blood samples [9–15]. Three of these studies were nested within the Alpha-Tocopherol, Beta-Carotene Cancer Prevention Study (ATBC) [11, 12, 14]; one within the Prostate, Lung, Colorectal and Ovarian Cancer Screening Trial (PLCO) [9]; one within the European Prospective Investigation into Cancer and Nutrition study (EPIC) in Heidelberg [10]; and two within the EPIC-multicentre cohort (Germany, Greece, Italy, the Netherlands, Spain and the UK) [13, 15]. The ABTC and PLCO studies used untargeted metabolomics to non-quantitatively measure many metabolites, many not targeted in the present study. The EPIC studies used the same MS-based methodology, allowing more straightforward comparisons with the present study.

The present study is the first to show positive associations between LPC C17:0 and risk of overall and aggressive prostate cancer (Table 3) [9–15]. The inverse association between acylcarnitine C18:2 and risk of aggressive prostate cancer in the present study (Table 3) is consistent with the association between higher concentrations of acylcarnitine C18:2 and lower risk of advanced-stage prostate cancer in the EPIC-multicentre studies [13, 15].

The positive association between LPC C18:0 and risk of overall prostate cancer (Table 3) in the present study was not observed in any of the EPIC-multicentre studies (ORs close to one) [13, 15]. In the EPIC-Heidelberg study, an inverse association between LPC C18:0 and overall prostate cancer risk (*p* < 0.05) was observed, but the association was no longer significant after correction for multiple testing or adjusting for confounding factors in that case-cohort study [10]. The positive associations between glycerophospholipids (LPCs and PCs) and risk of aggressive prostate cancer in our study are in the opposite direction to the inverse associations between glycerophospholipids and risk of advanced-stage prostate cancer reported in the EPIC-multicentre studies [13, 15].

In this study, we only used overnight fasting samples, because varying time since the last meal can generate variations in the concentration of several metabolites that are not related to the estimation of disease risk. This was not considered in the experimental design of the EPIC studies [10, 13, 15]. Instead, the EPIC-multicentre studies matched each case-control set based on categories of time since last meal (< 3, 3–6, > 6 h) [13, 15]. However, the range of variation in the concentration of some metabolites within each of those time categories is still large. For examples, we have previously shown that LPC concentrations change by 25–34% compared with baseline within 3 h after a meal [16]. Therefore, our study population was (at least in theory) subjected to lower pre-analytical variation and this may have assisted in revealing the association between, for example, LPC C18:0 and higher risk of overall prostate cancer.

Moreover, in the present study, as in some previous studies [19, 24], the aggressive disease subtype included both high grade and non-localised tumours (T3–4). In contrast, the EPIC-multicentre studies considered only non-localised tumours (T3–4) in the advanced-stage disease subtype [13, 15]. These differences in disease subtype categorisation may partly account for the different associations observed between our study and the EPIC-multicentre studies. At this stage, it is not possible to determine the exact reason for the opposing association between LPCs and risk of aggressive/advanced-stage prostate cancer observed in the present study and the EPIC-multicentre studies. Further research, for example comparing our nested cohort with a subgroup in the EPIC-multicentre cohort and including only case-controls with samples collected after overnight fasting, may help explain the discrepancies. This suggestion is motivated by our previous observation that the extent of change in the concentrations of metabolites (i.e. phospholipids) after a meal is also associated with the physiological status of individuals (in addition to time since last meal) [16]. For example, the post-meal changes in several metabolites are blunted in individuals with impaired glucose/insulin metabolism [16]. This may indicate that the association of a metabolite with a disease may switch direction or be affected when moving from fasting samples to post-meal samples if the extent of post-meal change in the metabolite differs between cases and controls, as a result of disease-associated physiological status.

Sarcosine has been prospectively associated with risk of prostate cancer in a study performed in the PLCO [31]. No association between sarcosine and risk of prostate cancer was found in the present study (data not shown). One reason may be the shorter follow-up times used in the PLCO study (≥ 1 year) compared with the present study (≥ 5 years). Intriguingly, no association with sarcosine was found in another study performed in the PLCO that used longer follow-up times (≥ 4.4 years) [9]. This might suggest sarcosine is rather a marker of (early) diagnosis [31, 32] or progression [33, 34] in prostate cancer.

### Investigating links between prostate cancer and dietary fatty acids

After normalising the concentration of each LPC with ≤ 20 carbons to the total concentration of such LPCs, only LPC C17:0 remained associated with risk of prostate cancer (Fig. 2b; OR > 1; *p* < 0.05). This may imply an increase at two levels: (1) an overall increase in LPCs and (2) an additional increase in the LPC with 17 carbons in the FA moiety (LPC C17:0). Moreover, the LPC C17:0 concentration in plasma displayed a significant positive correlation with dietary intake of the corresponding FA (FA C17:0; Additional file 8), while for other LPCs, such a correlation was generally weak or absent.

Odd-chain FAs (e.g. C17:0) are found in products of ruminant origin (e.g. dairy products). Previous studies have shown a positive correlation between levels of plasma LPC C17:0 and dairy consumption [35, 36]. Therefore, our findings may suggest a link between consumption of dairy products and risk of prostate cancer. Some previous studies have also suggested a higher prostate cancer risk with higher intake of dairy products [37, 38], although evidence of such an association is still limited [5], and therefore, it warrants further investigation.

A previous study in the Pan-European EPIC-cohort reported dairy consumption to be higher among males in northern Sweden than in many other European countries [39]. If high dairy consumption has implications for prostate cancer, then metabolic markers of dairy consumption (e.g. LPC C17:0) [35, 36] are more likely to display a stronger association to prostate cancer risk in a population such as northern Sweden.

It should be borne in mind that according to recent findings, biosynthesis can also contribute to circulatory levels of FA C17:0 [40]. Hence, more research is needed to enable sound conclusions to be drawn. Understanding the link between consumption of dairy products and risk of prostate cancer would be of great value in formulating prevention strategies, since consumption of dairy products is a modifiable risk factor.

### Investigating the link between prostate cancer and glucose metabolism

Higher concentrations of LPC C17:0, LPC C18:0 and glycine, which were associated with higher risk of overall prostate cancer in the present study (Table 3), were found to be associated with lower risk of IGT and T2D in a previous study [41]. Similarly, FA C17:0 has been linked to glucose intolerance [40]. In addition, pyruvate, which we observed to be associated with lower risk of overall prostate cancer, is an important intermediate in glucose metabolism [42]. These findings suggest a possible link between glucose metabolism and risk of prostate cancer. In addition, aberrant glucose metabolism [24, 43] and T2D [44, 45] have previously been associated with lower risk of prostate cancer. Therefore, we investigated the correlation between metabolites frequently associated with a risk of prostate cancer (LPCs) and glucose concentrations (0 h and 2 h) from an OGTT performed on all subjects at baseline. Intriguingly, we detected negative correlations between LPCs (with ≤ 20 carbons in the FA moiety) and glucose concentrations.

Considering the negative correlation between LPCs and glucose concentrations, we then investigated whether the status of glucose metabolism at baseline (i.e. IGT, IFG and T2D) was associated with risk of prostate cancer. It was observed that IGT was associated with a lower risk of prostate cancer (Fig. 3b). Next, we postulated that the association between metabolites and prostate cancer risk may appear stronger in a population with NGT, where the risk-reducing (masking) effect of IGT does not exist. Therefore, we repeated the statistical analyses among case-controls with NGT for metabolites associated with prostate cancer risk (Table 3). We found that the associations appeared stronger for many of the glycerophospholipids and glycine, supporting our postulated effect (Fig. 4). Overall, these findings show that the reverse cross-association between T2D and prostate cancer risk [44, 45] can be observed at metabolite level.

A number of studies have shown altered metabolic regulation in relation to the development [9–15] and progression [46] of prostate cancer. It would be of great interest to acquire insights into the genes involved in controlling these metabolic alterations. The cross-association between T2D and prostate cancer observed in the present and previous studies [44, 45] may warrant further research on genes and signalling pathways at the intercept of prostate cancer and T2D. One such signalling pathway is phosphatase and tensin homologue (PTEN)-PI3K/AKT with known implications in both prostate cancer and diabetes [47, 48].

## Conclusions

We observed associations of LPCs, PCs, glycine, acylcarnitine C18:2 and pyruvate with prostate cancer risk, with the associations varying with baseline age and disease aggressiveness. The strongest and most consistent association was observed between LPC C17:0, potentially a marker of dairy consumption, and higher risk of overall and aggressive prostate cancer. Moreover, several LPCs that were associated with risk of prostate cancer were negatively correlated with blood glucose concentrations from an OGTT. The association of glycerophospholipids and glycine with prostate cancer risk was stronger in case-controls with NGT. These findings suggest a possible link between glucose metabolism and the risk of developing prostate cancer.

## Supplementary information

**Additional file 1.** Methods for targeted MS and NMR-based metabolomics.

**Additional file 2.** Quality control of metabolites quantified with targeted metabolomics.

**Additional file 3.** Associations between individual metabolites and prostate cancer risk.

**Additional file 4.** Associations significant after correction for multiple testing.

**Additional file 5.** Differential associations by age group and disease type.

**Additional file 6.** Associations with overall prostate cancer risk after stratification by follow-up time.

**Additional file 7.** Adjusting for the sum of lysophosphatidylcholines with ≤20 carbons.

**Additional file 8.** Correlation of lysophosphatidylcholines with dietary fatty acids.

**Additional file 9.** Status of glucose metabolism and prostate cancer risk.

**Additional file 10.** Prostate cancer risk in case-controls with normal glucose tolerance.

## Data Availability

The data used in the present study are available to the editorial board for evaluating the findings presented. In order to access the data and samples for another study, after receiving ethical approval, a request should be submitted to the Biobank Research Unit at Umeå University, which administers and conveys information about access to data and samples in this study (https://www.umu.se/en/biobank-research-unit/).

## Abbreviations

ABTC: Alpha-Tocopherol, Beta-Carotene Cancer Prevention Study
AQuA: Automated quantification algorithm
BMI: Body mass index
CI: Confidence interval
CV: Coefficient of variation
DBT: Diastolic blood pressure
EPIC: European Prospective Investigation into Cancer and Nutrition study
FA: Fatty acid
FDR: False discovery rate
FFQ: Food frequency questionnaire
IGF-I: Insulin-like growth factor I
IGT: Impaired glucose tolerance
IFG: Impaired fasting glucose
LPC: Lysophosphatidylcholine
MS: Mass spectrometry
NGT: Normal glucose tolerance
NMR: Nuclear magnetic resonance
NSHDS: Northern Sweden Health and Disease Study
OGTT: Oral glucose tolerance test
OR: Odds ratio
PC: Phosphatidylcholine
PLCO: Prostate, Lung, Colorectal and Ovarian Cancer Screening Trial
PTEN: Phosphatase and tensin homologue
QC: Quality control
SBT: Systolic blood pressure
T2D: Type 2 diabetes
WHO: World Health Organization
