# Supplementary material for: Identification of metabolites associated with prostate cancer risk: a nested case-control study with long follow-up in the Northern Sweden Health and Disease Study
Source: BMC Med. 2020 Jul 23;18:187. doi: 10.1186/s12916-020-01655-1 (PMC7376662; doi:10.1186/s12916-020-01655-1)
Supplement: Supplementary file 1 — Additional file 1. Methods for targeted MS and NMR-based metabolomics. [file 12916_2020_1655_MOESM1_ESM.pdf]

Methods for targeted MS and NMR-based metabolomics

Targeted MS-based metabolomics: Plasma samples from the case-controls in the nested cohort (n = 1554) were analysed with the AbsoluteIDQ p180 assay (BIOCRATES, Innsbruck, Austria) [1, 2], a targeted mass spectrometry (MS)-based metabolomics approach for quantification of 188 metabolites, including 40 acylcarnitines, 41 amino acids/biogenic amines, 77 phosphatidylcholines with an acyl-acyl residue (PC aa) or an acyl-alkyl residue (PC ae), 14 lysophosphatidylcholines (LPC), 15 sphingomyelins (SM, SM-OH) and one hexose (H). Each lipid species was abbreviated to Cx:y, based on the number of carbon atoms (x) and the number of double bonds (y) in the fatty acid (FA) moiety.

The targeted MS-based metabolomics approach used in the present study, and the required instrumentation, has been described in detail elsewhere [1, 2]. The approach has also been used in other epidemiological studies to identify metabolites associated with the risk of disease [3-5]. In brief, an internal standard solution was added to each sample, followed by derivatisation with phenylisothiocyanate. Amino acids and biogenic amines were analysed with liquid chromatography (LC) coupled to MS/MS, while acylcarnitines, lipids and hexose were analysed using flow injection analysis (FIA)-MS/MS. The case-control samples were analysed randomly in 20 batches, using an AB SCIEX QTrap® mass spectrometer (AB SCIEX, Darmstadt, Germany) equipped with electrospray ionisation. Quality control (QC) human plasma samples were incorporated into the workflow along with the case-control samples, in order to evaluate the analytical coefficient of variation (CV).

Data processing, to generate metabolite concentrations in each sample, was performed automatically using the MetIDQ™ software. Beside metabolite quantification, it was also determined whether each measurement was above or below the detection limit. Hence, it was possible to determine the occurrence for each metabolite – i.e. the percentage of measurements found to be above the detection limit in the case-control samples (Additional file 2).

Targeted NMR-based metabolomics: Plasma samples from the case-controls were also analysed with targeted nuclear magnetic resonance (NMR)-based metabolomics. The NMR-based metabolomics approach employed an automated approach (called AQuA, see below) for quantification of target metabolites from one-dimensional proton (1D; ^{1}H) NMR spectra that was specifically developed in our laboratory for large-scale epidemiological studies [6].

Sample preparation was performed as previously described [7] after modification for using small sample volumes [6]. In brief, macromolecules were removed from each sample (60 µL) using ultrafiltration. The filtrate was then mixed with phosphate buffer, deuterated water/water (D_2O/H_2O), and an internal standard (3-methylsilyl propionate-d_4; TSP-d_4). Each sample was then analysed with 1D ^{1}H NMR on a Bruker spectrometer operating at 600 MHz, which was equipped with a cryogenically cooled probe and an autosampler. Each experimental ^{1}H NMR spectrum was obtained using a zgesgp pulse sequence (Bruker Biospin; 512 scans), where the water signal was supressed by excitation sculpting and pulsed-field gradients [8]. The samples were analysed in 27 batches, with each case-control pair analysed together. As in the targeted MS-based metabolomics approach, the analytical CV was assessed by incorporating unspiked QC samples of pooled human plasma into the workflow (three replicates per batch).

Each experimental spectrum was processed manually, including phase correction and line width adjustment via line broadening (TSP-d_4 full-width at half-maximum of 1.0 Hz; 341.2 μM) using the ChenomX NMR Suite software (version 7.5, ChenomX Inc., Edmonton, Canada).

Quantification of 67 human plasma metabolites (Additional file 2) was carried out using an automated quantification algorithm (AQuA) that accounted for signal interferences [6]. The AQuA approach is based on automated library-based computations wherein one pre-selected ^{1}H NMR signal per metabolite is used to determine its concentration. AQuA was implemented in MATLAB (version R2012b; Mathworks Inc.; USA). AQuA also enabled determination of detection limits, which were used to assess the occurrence of each metabolite across the samples from the case-controls. NMR spectra were not obtained for 30 samples and therefore only 747 (out of 777) case-control pairs could be included in the statistical analyses, which were based on the NMR data.

References in supplementary methods

1. Ramsay SL, Stoegg KM, Weinberger A, Graber A, Guggenbichler W. Apparatus and methods for analyzing a metabolite profile. EP 1875401 A211-Jan-2007.
2. Bogmuil R, Koal T, Weinberger KM, Dammaeier S. Targeted metabolomics: fast, standardized mass spectrometric analysis of blood plasma with a kit. Laborwelt. 2008;2:17-23.
3. Schmidt JA, Fensom GK, Rinaldi S, Scalbert A, Appelby PN, Achaintre D, et al. Pre-diagnostic metabolite concentrations and prostate cancer risk in1077 matched controls in the European prospective investigation into cancer and nutrition. BMC Med. 2017;15:122.
4. Floegel A, Stefan N, Yu Z, Mühlenbruch K, Drogan D, Joost HG, et al. Identification of serum metabolites associated with risk of type 2 diabetes using a targeted metabolomic approach. Diabetes. 2013;62:639-48.
5. Tynkkynen J, Chouraki V, van der Lee SJ, Hernesniemi J, Yang Q, Li S, et al. Association of branched-chain amino acids and other circulating metabolites with risk of incident dementia and Alzheimer's disease: a prospective study in eight cohorts. Alzheimers Dement 2018;14:723-33.
6. Röhnisch HE, Eriksson J, Müllner E, Agback P, Sandström C, Moazzami AA. AQuA: an automated quantification algorithm for high-throughput NMR-based metabolomics and its application in human plasma. Anal Chem. 2018;90:2095-102.
7. Tiziani S, Emwas AH, Lodi A, Ludwig C, Bunce CM, Viant MR, Günther UL. Optimized metabolite extraction from blood serum for ^{1}H nuclear magnetic resonance spectroscopy. Anal Biochem. 2008;377:16-23.
8. Hwang TL, Shaka AJ. Water suppression that works. Excitation sculpting using arbitrary wave-forms and pulsed-field gradients. J Mangn Reson A. 1995;112:275-9.
