# Supplementary material for: Identification of metabolites associated with prostate cancer risk: a nested case-control study with long follow-up in the Northern Sweden Health and Disease Study
Source: BMC Med. 2020 Jul 23;18:187. doi: 10.1186/s12916-020-01655-1 (PMC7376662; doi:10.1186/s12916-020-01655-1)
Supplement: Supplementary file 2 — Additional file 2. Quality control of metabolites quantified with targeted metabolomics. [file 12916_2020_1655_MOESM2_ESM.pdf]

**Additional file 2:** Quality control of metabolites quantified with targeted metabolomics

| Name | Abbreviation | Occurrence [a] (%) | $CV_{QC}$ [b] (%) | $CV_{tot}$ [c] (%) | Included [d] |
|---|---|---|---|---|---|
| **Metabolites measured with MS** | | | | | |
| Carnitine (free) | C0 | 100 | 7.3 | 19.7 | Yes |
| Acetylcarnitine | C2 | 100 | 7.7 | 35.1 | Yes |
| Propionylcarnitine | C3 | 100 | 12.3 | 28.7 | Yes |
| Hydroxybutyrylcarnitine | C3-DC | 75.7 | 19.8 | 64.2 | No |
| Hydroxypropionylcarnitine | C3-OH | 0.1 | NA | NA | No |
| Propenoylcarnitine | C3:1 | 0.3 | NA | 31.7 | No |
| Butyrylcarnitine | C4 | 100 | 10.4 | 41.1 | Yes |
| Butenoylcarnitine | C4:1 | 3.6 | 13.6 | 30.3 | No |
| Isovalerylcarnitine | C5 | 97.4 | 13.0 | 25.6 | Yes |
| Glutarylcarnitine | C5-DC | 21.2 | 10.8 | 28.1 | No |
| Methylglutarylcarnitine | C5-M-DC | 0.1 | NA | NA | No |
| Hydroxyisovalerylcarnitine | C5-OH | 14.8 | 31.0 | 30.6 | No |
| Tiglylcarnitine | C5:1 | 0.3 | NA | 11.3 | No |
| Glutaconylcarnitine | C5:1-DC | 4.9 | NA | 112.8 | No |
| Hexanoylcarnitine | C6 | 20.9 | NA | 31.0 | No |
| Hexenoylcarnitine | C6:1 | 0.1 | NA | NA | No |
| Pimelylcarnitine | C7-DC | 70.1 | 20.4 | 33.6 | No |
| Octanoylcarnitine | C8 | 33.7 | 59.3 | 41.7 | No |
| Nonanoylcarnitine | C9 | 77.4 | 30.8 | 34.4 | No |
| Decanoylcarnitine | C10 | 89.3 | 11.2 | 43.9 | Yes |
| Decenoylcarnitine | C10:1 | 2.2 | NA | 27.6 | No |
| Decadienoylcarnitine | C10:2 | 39.2 | 2.8 | 22.1 | No |
| Dodecanoylcarnitine | C12 | 65.6 | 20.6 | 32.7 | No |
| Dodecanedioylcarnitine | C12-DC | 0.0 | NA | NA | No |
| Dodecenoylcarnitine | C12:1 | 2.5 | NA | 14.5 | No |
| Tetradecanoylcarnitine | C14 | 78.4 | 32.9 | 29.0 | No |
| Tetradecenoylcarnitine | C14:1 | 100 | 15.6 | 31.0 | No |
| Hydroxytetradecenoylcarnitine | C14:1-OH | 21.3 | NA | 29.7 | No |
| Tetradecadienoylcarnitine | C14:2 | 64 | 18.7 | 35.0 | No |
| Hydroxytetradecadienoylcarnitine | C14:2-OH | 12.7 | 9.8 | 54.1 | No |
| Hexadecanoylcarnitine | C16 | 100 | 11.1 | 22.5 | Yes |
| Hydroxyhexadecanolycarnitine | C16-OH | 8.2 | NA | 32.0 | No |
| Hexadecenoylcarnitine | C16:1 | 7.6 | NA | 21.1 | No |
| Hydroxyhexadecanolycarnitine | C16:1-OH | 11.9 | NA | 56.5 | No |
| Hexadecadienoylcarnitine | C16:2 | 22.7 | 18.7 | 29.3 | No |
| Hydroxyhexadecadienoylcarnitine | C16:2-OH | 0.1 | NA | NA | No |
| Octadecanoylcarnitine | C18 | 100 | 17.4 | 25.5 | No |
| Octadecenoylcarnitine | C18:1 | 99.4 | 8.9 | 25.6 | Yes |
| Hydroxyoctadecenoylcarnitine | C18:1-OH | 0.3 | NA | 72.6 | No |
| Octadecadienoylcarnitine | C18:2 | 100 | 11.7 | 31.0 | Yes |
| Alanine | Ala | 100 | 10.7 | 24.9 | Yes |
| Arginine | Arg | 100 | 11.8 | 25.4 | Yes |
| Asparagine | Asn | 100 | 21.2 | 21.3 | No |
| Aspartate | Asp | 99.2 | 16.8 | 38.2 | No |
| Citrulline | Cit | 100 | 23.5 | 28.8 | No |
| Glutamine | Gln | 100 | 18.6 | 22.5 | No |
| Glutamate | Glu | 100 | 22.6 | 57.7 | No |
| Glycine | Gly | 100 | 16.0 | 27.1 | No |
| Histidine | His | 100 | 11.4 | 15.4 | Yes |
| Isoleucine | Ile | 100 | 13.1 | 19.6 | Yes |
| Leucine | Leu | 100 | 10.2 | 18.2 | Yes |
| Lysine | Lys | 100 | 14.0 | 19.4 | Yes |
| Methionine | Met | 100 | 16.0 | 22.7 | No |
| Ornithine | Orn | 100 | 13.6 | 27.5 | Yes |
| Phenylalanine | Phe | 100 | 10.5 | 15.3 | Yes |

**Additional file 2:** (Continued)

| Name | Abbreviation | Occurrence [a] (%) | $CV_{QC}$ [b] (%) | $CV_{tot}$ [c] (%) | Included [d] |
|---|---|---|---|---|---|
| Proline | Pro | 100 | 11.4 | 25.2 | Yes |
| Serine | Ser | 100 | 19.8 | 28.0 | No |
| Threonine | Thr | 100 | 15.6 | 23.0 | No |
| Tryptophan | Trp | 100 | 14.5 | 19.5 | Yes |
| Tyrosine | Tyr | 100 | 13.3 | 22.8 | Yes |
| Valine | Val | 100 | 20.2 | 22.7 | No |
| Acetylornithine | Ac-Orn | 0.4 | 25.2 | 53.5 | No |
| Asymmetric dimethylarginine | ADMA | 89.4 | 23.3 | 33.8 | No |
| Alpha-aminoadipic acid | Alpha-AAA | 99.2 | 20.6 | 45.4 | No |
| Carnosine | - | 14.2 | 52.0 | 137.1 | No |
| Creatinine | - | 100 | 5.4 | 14.3 | Yes |
| Dihydroxyphenylalanine | DOPA | 19.1 | 55.5 | 49.4 | No |
| Dopamine | - | 2.1 | NA | 122.1 | No |
| Histamine | - | 0.7 | NA | 77.0 | No |
| Kynurenine | - | 100 | 16.8 | 25.5 | No |
| Methionine-sulfoxide | Met-SO | 100 | 19.5 | 76.4 | No |
| Nitrotyrosine | Nitro-Tyr | 2.7 | NA | 116.4 | No |
| Hydroxyproline | OH-Pro | 70.6 | 72.3 | 58.9 | No |
| Phenylethylamine | PEA | 0.2 | NA | 17.5 | No |
| Putrescine | - | 0.0 | NA | NA | No |
| Sarcosine | - | 100 | 15.1 | 30.4 | No |
| Symmetric dimethylarginine | SDMA | 99.7 | 46.1 | 52.4 | No |
| Serotonin | - | 100 | 40.1 | 129.2 | No |
| Spermidine | - | 99.0 | 12.6 | 48.6 | Yes |
| Spermine | - | 18.1 | 0.4 | 240.5 | No |
| Taurine | - | 100 | 11.4 | 26.1 | Yes |
| Phosphatidylcholine (diacyl) Cx:y | PC aa C24:0 | 31.2 | 14.4 | 62.0 | No |
| Phosphatidylcholine (diacyl) Cx:y | PC aa C26:0 | 0.8 | NA | 31.1 | No |
| Phosphatidylcholine (diacyl) Cx:y | PC aa C28:1 | 100 | 7.9 | 22.4 | Yes |
| Phosphatidylcholine (diacyl) Cx:y | PC aa C30:0 | 100 | 9.9 | 29.3 | Yes |
| Phosphatidylcholine (diacyl) Cx:y | PC aa C30:2 | 0.1 | NA | NA | No |
| Phosphatidylcholine (diacyl) Cx:y | PC aa C32:0 | 100 | 9.5 | 20.1 | Yes |
| Phosphatidylcholine (diacyl) Cx:y | PC aa C32:1 | 100 | 9.9 | 46.7 | Yes |
| Phosphatidylcholine (diacyl) Cx:y | PC aa C32:2 | 100 | 12.1 | 37.0 | Yes |
| Phosphatidylcholine (diacyl) Cx:y | PC aa C32:3 | 100 | 15.1 | 27.2 | No |
| Phosphatidylcholine (diacyl) Cx:y | PC aa C34:1 | 100 | 9.1 | 22.0 | Yes |
| Phosphatidylcholine (diacyl) Cx:y | PC aa C34:2 | 100 | 12.3 | 19.3 | Yes |
| Phosphatidylcholine (diacyl) Cx:y | PC aa C34:3 | 100 | 12.2 | 25.1 | Yes |
| Phosphatidylcholine (diacyl) Cx:y | PC aa C34:4 | 100 | 12.5 | 31.4 | Yes |
| Phosphatidylcholine (diacyl) Cx:y | PC aa C36:0 | 100 | 14.2 | 34.9 | Yes |
| Phosphatidylcholine (diacyl) Cx:y | PC aa C36:1 | 100 | 11.7 | 22.9 | Yes |
| Phosphatidylcholine (diacyl) Cx:y | PC aa C36:2 | 100 | 10.0 | 18.7 | Yes |
| Phosphatidylcholine (diacyl) Cx:y | PC aa C36:3 | 100 | 8.8 | 19.6 | Yes |
| Phosphatidylcholine (diacyl) Cx:y | PC aa C36:4 | 100 | 9.7 | 21.3 | Yes |
| Phosphatidylcholine (diacyl) Cx:y | PC aa C36:5 | 100 | 10.4 | 46.6 | Yes |
| Phosphatidylcholine (diacyl) Cx:y | PC aa C36:6 | 100 | 11.8 | 36.4 | Yes |
| Phosphatidylcholine (diacyl) Cx:y | PC aa C38:0 | 100 | 12.7 | 28.5 | Yes |
| Phosphatidylcholine (diacyl) Cx:y | PC aa C38:1 | 96.8 | 22.4 | 42.5 | No |
| Phosphatidylcholine (diacyl) Cx:y | PC aa C38:3 | 100 | 9.0 | 22.1 | Yes |
| Phosphatidylcholine (diacyl) Cx:y | PC aa C38:4 | 100 | 10.1 | 23.1 | Yes |
| Phosphatidylcholine (diacyl) Cx:y | PC aa C38:5 | 100 | 8.6 | 24.7 | Yes |
| Phosphatidylcholine (diacyl) Cx:y | PC aa C38:6 | 100 | 8.6 | 29.2 | Yes |
| Phosphatidylcholine (diacyl) Cx:y | PC aa C40:1 | 72.8 | 13.9 | 24.0 | Yes |
| Phosphatidylcholine (diacyl) Cx:y | PC aa C40:2 | 100 | 16.0 | 38.3 | No |
| Phosphatidylcholine (diacyl) Cx:y | PC aa C40:3 | 100 | 12.6 | 25.7 | Yes |
| Phosphatidylcholine (diacyl) Cx:y | PC aa C40:4 | 100 | 11.3 | 28.4 | Yes |

**Additional file 2:** (Continued)

| Name | Abbreviation | Occurrence [a] (%) | $CV_{QC}$ [b] (%) | $CV_{tot}$ [c] (%) | Included [d] |
|---|---|---|---|---|---|
| Phosphatidylcholine (diacyl) Cx:y | PC aa C40:5 | 100 | 8.9 | 26.7 | Yes |
| Phosphatidylcholine (diacyl) Cx:y | PC aa C40:6 | 100 | 8.7 | 32.3 | Yes |
| Phosphatidylcholine (diacyl) Cx:y | PC aa C42:0 | 100 | 14.1 | 28.9 | Yes |
| Phosphatidylcholine (diacyl) Cx:y | PC aa C42:1 | 100 | 13.2 | 28.7 | Yes |
| Phosphatidylcholine (diacyl) Cx:y | PC aa C42:2 | 100 | 16.0 | 28.5 | No |
| Phosphatidylcholine (diacyl) Cx:y | PC aa C42:4 | 100 | 13.6 | 34.5 | Yes |
| Phosphatidylcholine (diacyl) Cx:y | PC aa C42:5 | 100 | 13.1 | 29.0 | Yes |
| Phosphatidylcholine (diacyl) Cx:y | PC aa C42:6 | 100 | 15.7 | 31.7 | No |
| Phosphatidylcholine (acyl-alkyl) Cx:y | PC ae C30:0 | 99.4 | 34.8 | 33 | No |
| Phosphatidylcholine (acyl-alkyl) Cx:y | PC ae C30:1 | 27.8 | 66.8 | 62.7 | No |
| Phosphatidylcholine (acyl-alkyl) Cx:y | PC ae C30:2 | 100 | 15.0 | 33.4 | Yes |
| Phosphatidylcholine (acyl-alkyl) Cx:y | PC ae C32:1 | 100 | 16.6 | 25.4 | No |
| Phosphatidylcholine (acyl-alkyl) Cx:y | PC ae C32:2 | 100 | 19.7 | 26.9 | No |
| Phosphatidylcholine (acyl-alkyl) Cx:y | PC ae C34:0 | 100 | 11.8 | 25.5 | Yes |
| Phosphatidylcholine (acyl-alkyl) Cx:y | PC ae C34:1 | 100 | 11.4 | 20.1 | Yes |
| Phosphatidylcholine (acyl-alkyl) Cx:y | PC ae C34:2 | 100 | 9.3 | 25.0 | Yes |
| Phosphatidylcholine (acyl-alkyl) Cx:y | PC ae C34:3 | 100 | 12.4 | 28.6 | Yes |
| Phosphatidylcholine (acyl-alkyl) Cx:y | PC ae C36:0 | 100 | 27.9 | 52.4 | No |
| Phosphatidylcholine (acyl-alkyl) Cx:y | PC ae C36:1 | 100 | 11.0 | 23.0 | Yes |
| Phosphatidylcholine (acyl-alkyl) Cx:y | PC ae C36:2 | 100 | 10.7 | 22.2 | Yes |
| Phosphatidylcholine (acyl-alkyl) Cx:y | PC ae C36:3 | 100 | 10.2 | 22.6 | Yes |
| Phosphatidylcholine (acyl-alkyl) Cx:y | PC ae C36:4 | 100 | 9.7 | 24.5 | Yes |
| Phosphatidylcholine (acyl-alkyl) Cx:y | PC ae C36:5 | 100 | 10.4 | 27.4 | Yes |
| Phosphatidylcholine (acyl-alkyl) Cx:y | PC ae C38:0 | 100 | 13.4 | 29.3 | Yes |
| Phosphatidylcholine (acyl-alkyl) Cx:y | PC ae C38:1 | 58.9 | 12.5 | 80.4 | Yes |
| Phosphatidylcholine (acyl-alkyl) Cx:y | PC ae C38:2 | 100 | 13.5 | 31.4 | Yes |
| Phosphatidylcholine (acyl-alkyl) Cx:y | PC ae C38:3 | 100 | 10.0 | 24.5 | Yes |
| Phosphatidylcholine (acyl-alkyl) Cx:y | PC ae C38:4 | 100 | 8.8 | 20.7 | Yes |
| Phosphatidylcholine (acyl-alkyl) Cx:y | PC ae C38:5 | 100 | 8.7 | 20.1 | Yes |
| Phosphatidylcholine (acyl-alkyl) Cx:y | PC ae C38:6 | 100 | 11.3 | 26.6 | Yes |
| Phosphatidylcholine (acyl-alkyl) Cx:y | PC ae C40:0 | 100 | 12.0 | 28.7 | Yes |
| Phosphatidylcholine (acyl-alkyl) Cx:y | PC ae C40:1 | 100 | 12.4 | 23.5 | Yes |
| Phosphatidylcholine (acyl-alkyl) Cx:y | PC ae C40:2 | 100 | 10.3 | 23.2 | Yes |
| Phosphatidylcholine (acyl-alkyl) Cx:y | PC ae C40:3 | 100 | 10.0 | 33.3 | Yes |
| Phosphatidylcholine (acyl-alkyl) Cx:y | PC ae C40:4 | 100 | 15.1 | 23.3 | No |
| Phosphatidylcholine (acyl-alkyl) Cx:y | PC ae C40:5 | 100 | 10.0 | 23.1 | Yes |
| Phosphatidylcholine (acyl-alkyl) Cx:y | PC ae C40:6 | 100 | 9.5 | 25.3 | Yes |
| Phosphatidylcholine (acyl-alkyl) Cx:y | PC ae C42:0 | 11.5 | 13.2 | 20.3 | No |
| Phosphatidylcholine (acyl-alkyl) Cx:y | PC ae C42:1 | 100 | 14.9 | 25.5 | Yes |
| Phosphatidylcholine (acyl-alkyl) Cx:y | PC ae C42:2 | 100 | 12.0 | 22.9 | Yes |
| Phosphatidylcholine (acyl-alkyl) Cx:y | PC ae C42:3 | 100 | 13.1 | 22.7 | Yes |
| Phosphatidylcholine (acyl-alkyl) Cx:y | PC ae C42:4 | 100 | 10.7 | 23.7 | Yes |
| Phosphatidylcholine (acyl-alkyl) Cx:y | PC ae C42:5 | 100 | 8.3 | 21.2 | Yes |
| Phosphatidylcholine (acyl-alkyl) Cx:y | PC ae C44:3 | 98.8 | 11.7 | 32.8 | Yes |
| Phosphatidylcholine (acyl-alkyl) Cx:y | PC ae C44:4 | 99.9 | 11.3 | 24.3 | Yes |
| Phosphatidylcholine (acyl-alkyl) Cx:y | PC ae C44:5 | 100 | 9.9 | 25.5 | Yes |
| Phosphatidylcholine (acyl-alkyl) Cx:y | PC ae C44:6 | 100 | 10.5 | 25.5 | Yes |
| Lysophosphatidylcholine Cx:y | LPC C14:0 | 0.1 | NA | NA | No |
| Lysophosphatidylcholine Cx:y | LPC C16:0 | 100 | 13.3 | 23.1 | Yes |
| Lysophosphatidylcholine Cx:y | LPC C16:1 | 100 | 14.3 | 29.8 | Yes |
| Lysophosphatidylcholine Cx:y | LPC C17:0 | 100 | 11.7 | 28.6 | Yes |
| Lysophosphatidylcholine Cx:y | LPC C18:0 | 100 | 9.4 | 23.4 | Yes |
| Lysophosphatidylcholine Cx:y | LPC C18:1 | 100 | 9.7 | 25.0 | Yes |
| Lysophosphatidylcholine Cx:y | LPC C18:2 | 100 | 13.9 | 32.1 | Yes |
| Lysophosphatidylcholine Cx:y | LPC C20:3 | 100 | 9.3 | 26.6 | Yes |
| Lysophosphatidylcholine Cx:y | LPC C20:4 | 100 | 10.5 | 27.8 | Yes |

**Additional file 2:** (Continued)

| Name | Abbreviation | Occurrence [a] (%) | $CV_{QC}$ [b] (%) | $CV_{tot}$ [c] (%) | Included [d] |
|---|---|---|---|---|---|
| Lysophosphatidylcholine Cx:y | LPC C24:0 | 87.9 | 12.5 | 63.7 | Yes |
| Lysophosphatidylcholine Cx:y | LPC C26:0 | 71.4 | 17.9 | 87.8 | No |
| Lysophosphatidylcholine Cx:y | LPC C26:1 | 95.0 | 17.6 | 81.8 | No |
| Lysophosphatidylcholine Cx:y | LPC C28:0 | 69.0 | 20.6 | 67.2 | No |
| Lysophosphatidylcholine Cx:y | LPC C28:1 | 100 | 17.1 | 44.4 | No |
| Hydroxysphingomyelin Cx:y | SM-OH C14:1 | 100 | 12.1 | 23.0 | Yes |
| Hydroxysphingomyelin Cx:y | SM-OH C16:1 | 100 | 12.4 | 23.2 | Yes |
| Hydroxysphingomyelin Cx:y | SM-OH C22:1 | 100 | 13.0 | 23.1 | Yes |
| Hydroxysphingomyelin Cx:y | SM-OH C22:2 | 100 | 23.3 | 22.3 | No |
| Hydroxysphingomyelin Cx:y | SM-OH C24:1 | 100 | 26.7 | 31.0 | No |
| Sphingomyelin Cx:y | SM C16:0 | 100 | 12.6 | 18.7 | Yes |
| Sphingomyelin Cx:y | SM C16:1 | 100 | 11.3 | 18.5 | Yes |
| Sphingomyelin Cx:y | SM C18:0 | 100 | 11.3 | 20.6 | Yes |
| Sphingomyelin Cx:y | SM C18:1 | 100 | 11.9 | 21.9 | Yes |
| Sphingomyelin Cx:y | SM C20:2 | 99.9 | 18.3 | 48.9 | No |
| Sphingomyelin Cx:y | SM C22:3 | 4.4 | 85.0 | 123.3 | No |
| Sphingomyelin Cx:y | SM C24:0 | 100 | 20.1 | 27.1 | No |
| Sphingomyelin Cx:y | SM C24:1 | 100 | 12.0 | 20.6 | Yes |
| Sphingomyelin Cx:y | SM C26:0 | 99.9 | 70.2 | 42.5 | No |
| Sphingomyelin Cx:y | SM C26:1 | 99.9 | 54.6 | 36.8 | No |
| Hexoses | H1 | 100 | 10.6 | 16.7 | Yes |
| **Metabolites measured by NMR** | | | | | |
| 1-Methylguanidine | - | 12.9 | 9.6 | 43.3 | No |
| 1-Methylhistidine | - | 0.1 | 22.2 | 27.0 | No |
| 1, 2-Propanediol | - | 35.8 | 6.3 | 51.9 | No |
| 2-Aminobutyrate | - | 100 | 11.5 | 23.3 | Yes |
| 2-Hydroxybutyrate | - | 100 | 5.5 | 34.6 | Yes |
| 2-Hydroxyisovalerate | - | 71.5 | 7.0 | 71.0 | Yes |
| 2-Ketoglutarate | - | 95.0 | 9.1 | 19.4 | Yes |
| 2-Oxoisocaproate | - | 99.5 | 9.8 | 24.8 | Yes |
| 2-Propanol | - | 41.8 | 18.9 | 688.9 | No |
| 3-Hydroxybutyrate | - | 100 | 6.1 | 123.3 | Yes |
| 3-Hydroxyisovalerate | - | 96.5 | 8.9 | 23.5 | Yes |
| 3-Methyl-2-oxovalerate | - | 63.0 | 30.5 | 37.6 | No |
| 3-Methylhistidine | - | 0.7 | 23.4 | 78.2 | No |
| Acetate | - | 100 | 8.1 | 64.1 | Yes |
| Acetoacetate | - | 94.9 | 6.8 | 105.5 | Yes |
| Acetone | - | 100 | 15.5 | 57.7 | No |
| Acetylcarnitine | - | 100 | 6.2 | 30.0 | Yes |
| Acetylglycine | - | 37.6 | 13.4 | 48.1 | No |
| Alanine | - | 100 | 5.7 | 22.9 | Yes |
| Arginine | - | 94.7 | 8.7 | 14.5 | Yes |
| Asparagine | - | 97.7 | 6.9 | 14.9 | Yes |
| Aspartate | - | 0.3 | 15.8 | 24.9 | No |
| Betaine | - | 100 | 7.6 | 17.3 | Yes |
| Carnitine | - | 100 | 5.9 | 18.8 | Yes |
| Choline | - | 100 | 5.8 | 20.5 | Yes |
| Citrate | - | 100 | 6.2 | 21.7 | Yes |
| Creatine | - | 100 | 6.4 | 52.7 | Yes |
| Creatinine | - | 100 | 6.5 | 17.3 | Yes |
| Dimethylamine | - | 1.5 | 12.1 | 44.8 | No |
| Dimethylglycine | - | 99.6 | 6.4 | 40.7 | Yes |
| Dimethylsulfone | - | 100 | 7.3 | 45.5 | Yes |
| Ethanol | - | 96.2 | 105.6 | 506.7 | No |
| Formate | - | 99.3 | 11.3 | 33.5 | Yes |
| Glucose | - | 100 | 5.8 | 15.8 | Yes |

**Additional file 2:** (Continued)

| Name | Abbreviation | Occurrence [a] (%) | $CV_{QC}$ [b] (%) | $CV_{tot}$ [c] (%) | Included [d] |
|---|---|---|---|---|---|
| Glutamate | - | 82.6 | 7.3 | 51.4 | Yes |
| Glutamine | - | 100 | 5.8 | 13.0 | Yes |
| Glycerol | - | 100 | 70.7 | 71.5 | No |
| Glycine | - | 100 | 7.4 | 20.4 | Yes |
| Hippurate | - | 0.1 | 27.1 | 38.3 | No |
| Histidine | - | 100 | 5.9 | 12.9 | Yes |
| Hypoxanthine | - | 0.7 | 20.0 | 81.4 | No |
| Isobutyrate | - | 0.3 | 25.7 | 56.8 | No |
| Isocaproate | - | 99.1 | 7.2 | 8.5 | Yes |
| Isoleucine | - | 100 | 5.8 | 18.6 | Yes |
| Isovalerate | - | 0.3 | 55.3 | 123.1 | No |
| Lactate | - | 100 | 5.8 | 39.6 | Yes |
| Leucine | - | 100 | 5.6 | 16.3 | Yes |
| Lysine | - | 100 | 5.8 | 15.4 | Yes |
| Methanol | - | 100 | 45.2 | 62.0 | No |
| Methionine | - | 100 | 6.6 | 13.9 | Yes |
| Myo-inositol | - | 88.6 | 11.0 | 22.4 | Yes |
| O-phosphocholine | - | 98.8 | 13.7 | 29.8 | Yes |
| Ornithine | - | 100 | 5.8 | 23.0 | Yes |
| Phenylalanine | - | 99.7 | 6.9 | 13.8 | Yes |
| Proline | - | 100 | 7.5 | 25.8 | Yes |
| Propionate | - | 42.3 | 27.5 | 32.5 | No |
| Pyroglutamate | - | 1.5 | 25.1 | 29.1 | No |
| Pyruvate | - | 99.7 | 6.2 | 35.0 | Yes |
| Sarcosine | - | 12.5 | 8.9 | 23.6 | No |
| Glutamate | - | 82.6 | 7.3 | 51.4 | Yes |
| Glutamine | - | 100 | 5.8 | 13.0 | Yes |
| Glycerol | - | 100 | 70.7 | 71.5 | No |
| Glycine | - | 100 | 7.4 | 20.4 | Yes |
| Hippurate | - | 0.1 | 27.1 | 38.3 | No |
| Histidine | - | 100 | 5.9 | 12.9 | Yes |
| Hypoxanthine | - | 0.7 | 20.0 | 81.4 | No |
| Isobutyrate | - | 0.3 | 25.7 | 56.8 | No |
| Isocaproate | - | 99.1 | 7.2 | 8.5 | Yes |
| Isoleucine | - | 100 | 5.8 | 18.6 | Yes |
| Isovalerate | - | 0.3 | 55.3 | 123.1 | No |
| Lactate | - | 100 | 5.8 | 39.6 | Yes |
| Leucine | - | 100 | 5.6 | 16.3 | Yes |
| Lysine | - | 100 | 5.8 | 15.4 | Yes |
| Methanol | - | 100 | 45.2 | 62.0 | No |
| Methionine | - | 100 | 6.6 | 13.9 | Yes |
| Myo-inositol | - | 88.6 | 11.0 | 22.4 | Yes |
| O-phosphocholine | - | 98.8 | 13.7 | 29.8 | Yes |
| Ornithine | - | 100 | 5.8 | 23.0 | Yes |
| Phenylalanine | - | 99.7 | 6.9 | 13.8 | Yes |
| Proline | - | 100 | 7.5 | 25.8 | Yes |
| Propionate | - | 42.3 | 27.5 | 32.5 | No |
| Pyroglutamate | - | 1.5 | 25.1 | 29.1 | No |
| Pyruvate | - | 99.7 | 6.2 | 35.0 | Yes |
| Sarcosine | - | 12.5 | 8.9 | 23.6 | No |
| Serine | - | 100 | 6.0 | 19.5 | Yes |
| Succinate | - | 89.4 | 15.2 | 43.1 | No |
| Threonine | - | 99.7 | 6.9 | 16.3 | Yes |
| Trigonelline | - | 0.4 | 55.3 | 99.2 | No |
| Trimethylamine | - | 88.7 | 7.0 | 35.2 | Yes |
| Trimethylamine-N-oxide | - | 100 | 11.8 | 48.2 | Yes |

**Additional file 2:** (Continued)

| **Name** | **Abbreviation** | **Occurrence [a] (%)** | **$CV_{QC}$ [b] (%)** | **$CV_{tot}$ [c] (%)** | **Included [d]** |
|---|---|---|---|---|---|
| Tyrosine | - | 100 | 6.0 | 18.7 | Yes |
| Valine | - | 100 | 5.8 | 16.0 | Yes |

[a] Occurrence (%): Percentage of measurements above the detection limit in the nested cohort.

[b] $CV_{QC}$ (%): Analytical coefficient of variation, calculated based on the measurements in the unspiked quality control (QC) samples.

[c] $CV_{tot}$ (%): Total coefficient of variation, calculated based on the measurements in the nested cohort.

[d] Included (Yes): Occurrence $\geq$ 50%, and $CV_{QC} \leq$ 15%.
