# Supplementary material for: Identification of metabolites associated with prostate cancer risk: a nested case-control study with long follow-up in the Northern Sweden Health and Disease Study
Source: BMC Med. 2020 Jul 23;18:187. doi: 10.1186/s12916-020-01655-1 (PMC7376662; doi:10.1186/s12916-020-01655-1)
Supplement: Supplementary file 3 — Additional file 3. Associations between individual metabolites and prostate cancer risk. [file 12916_2020_1655_MOESM3_ESM.pdf]

Additional file 3: Association between individual metabolites prostate cancer risk

Metabolite	40-60 years [a]		40-50 years [a]		60 years [a]	
	OR_{crude} (95% CI)	p_{crude} [b]	OR_{crude} (95% CI)	p_{crude} [b]	OR_{crude} (95% CI)	p_{crude} [b]
**Metabolites measured by MS and overall prostate cancer risk [a]**						
C0	1.14 (0.80 − 1.61)	0.4763	1.32 (0.76 − 2.27)	0.3220	1.02 (0.65 − 1.62)	0.9258
C2	1.17 (0.93 − 1.45)	0.1762	1.12 (0.81 − 1.57)	0.4937	1.20 (0.89 − 1.61)	0.2305
C3	1.34 (1.05 − 1.71)	0.0206*	1.21 (0.84 − 1.75)	0.3166	1.45 (1.04 − 2.02)	0.0276*
C4	0.97 (0.80 − 1.18)	0.7738	0.95 (0.70 − 1.27)	0.7083	0.99 (0.76 − 1.30)	0.9593
C5	1.16 (0.88 − 1.53)	0.2880	1.15 (0.75 − 1.77)	0.5310	1.17 (0.82 − 1.67)	0.3895
C10	1.07 (0.89 − 1.28)	0.4977	1.07 (0.81 − 1.42)	0.6244	1.06 (0.83 − 1.35)	0.6366
C16	1.03 (0.75 − 1.41)	0.8732	1.05 (0.65 − 1.72)	0.8326	1.01 (0.66 − 1.53)	0.9774
C18:1	0.91 (0.69 − 1.20)	0.5182	1.06 (0.70 − 1.59)	0.7891	0.81 (0.56 − 1.17)	0.2633
C18:2	0.88 (0.70 − 1.12)	0.3082	0.90 (0.63 − 1.29)	0.5611	0.87 (0.63 − 1.20)	0.3970
Ala	0.96 (0.72 − 1.28)	0.7983	0.96 (0.63 − 1.46)	0.8575	0.97 (0.65 − 1.42)	0.8556
Arg	0.85 (0.64 − 1.11)	0.2296	0.66 (0.44 − 1.00)	0.0475*	1.04 (0.72 − 1.51)	0.8393
His	0.75 (0.48 − 1.19)	0.2259	1.05 (0.55 − 2.01)	0.8810	0.54 (0.28 − 1.04)	0.0645
Ile	1.05 (0.72 − 1.54)	0.7941	1.00 (0.57 − 1.73)	0.9881	1.10 (0.66 − 1.85)	0.7103
Leu	1.11 (0.75 − 1.66)	0.5993	1.16 (0.63 − 2.15)	0.6271	1.08 (0.63 − 1.83)	0.7839
Lys	0.79 (0.55 − 1.13)	0.1885	0.90 (0.52 − 1.55)	0.7021	0.71 (0.44 − 1.14)	0.1598
Orn	0.91 (0.69 − 1.19)	0.4932	1.38 (0.89 − 2.12)	0.1492	0.69 (0.48 − 0.98)	0.0400*
Phe	1.01 (0.64 − 1.59)	0.9728	0.96 (0.47 − 1.93)	0.9000	1.05 (0.58 − 1.90)	0.8792
Pro	1.10 (0.82 − 1.46)	0.5397	1.24 (0.79 − 1.93)	0.3528	1.00 (0.68 − 1.47)	0.9917
Trp	1.14 (0.80 − 1.63)	0.4583	1.04 (0.62 − 1.76)	0.8761	1.24 (0.76 − 2.01)	0.3877
Tyr	0.91 (0.66 − 1.27)	0.5916	0.81 (0.49 − 1.34)	0.4120	1.00 (0.65 − 1.55)	1.0000
Creatinine	1.44 (0.88 − 2.38)	0.1477	1.44 (0.63 − 3.26)	0.3846	1.45 (0.77 − 2.71)	0.2471
Spermidine	1.01 (0.87 − 1.17)	0.9199	0.89 (0.71 − 1.11)	0.2938	1.13 (0.92 − 1.39)	0.2604
Taurine	1.31 (0.97 − 1.77)	0.0838	0.90 (0.56 − 1.45)	0.6576	1.68 (1.13 − 2.50)	0.0107*
PC aa C28:1	1.01 (0.74 − 1.38)	0.9300	0.91 (0.58 − 1.42)	0.6646	1.12 (0.73 − 1.72)	0.5976
PC aa C30:0	1.08 (0.86 − 1.37)	0.5075	1.13 (0.79 − 1.60)	0.5075	1.05 (0.77 − 1.43)	0.7659
PC aa C32:0	1.11 (0.79 − 1.58)	0.5432	1.17 (0.69 − 2.01)	0.5599	1.07 (0.68 − 1.69)	0.7615
PC aa C32:1	0.92 (0.77 − 1.08)	0.3057	0.90 (0.71 − 1.15)	0.4030	0.93 (0.74 − 1.17)	0.5350
PC aa C32:2	0.95 (0.80 − 1.13)	0.5705	0.95 (0.74 − 1.22)	0.7100	0.95 (0.75 − 1.21)	0.6677
PC aa C34:1	1.07 (0.77 − 1.50)	0.6798	0.97 (0.58 − 1.64)	0.9182	1.15 (0.74 − 1.78)	0.5326
PC aa C34:2	1.09 (0.77 − 1.54)	0.6411	1.02 (0.59 − 1.75)	0.9497	1.14 (0.72 − 1.78)	0.5801
PC aa C34:3	1.00 (0.76 − 1.31)	0.9847	0.90 (0.59 − 1.36)	0.6063	1.08 (0.75 − 1.57)	0.6676
PC aa C34:4	1.13 (0.91 − 1.41)	0.2752	1.04 (0.76 − 1.42)	0.8079	1.23 (0.90 − 1.68)	0.1953
PC aa C36:0	1.18 (0.96 − 1.46)	0.1181	1.26 (0.92 − 1.72)	0.1498	1.12 (0.85 − 1.49)	0.4259
PC aa C36:1	1.06 (0.77 − 1.45)	0.7338	0.90 (0.54 − 1.49)	0.6857	1.17 (0.78 − 1.75)	0.4492
PC aa C36:2	1.11 (0.77 − 1.60)	0.5761	1.06 (0.59 − 1.89)	0.8453	1.14 (0.72 − 1.83)	0.5746
PC aa C36:3	1.14 (0.80 − 1.63)	0.4776	1.09 (0.62 − 1.90)	0.7740	1.18 (0.74 − 1.87)	0.4941
PC aa C36:4	1.36 (0.98 − 1.89)	0.0687	1.31 (0.77 − 2.20)	0.3182	1.40 (0.91 − 2.14)	0.1248
PC aa C36:5	1.18 (0.99 − 1.39)	0.0642	1.25 (0.96 − 1.63)	0.0971	1.12 (0.90 − 1.41)	0.3112
PC aa C36:6	1.18 (0.97 − 1.43)	0.1080	1.15 (0.86 − 1.54)	0.3340	1.19 (0.91 − 1.56)	0.1955
PC aa C38:0	1.18 (0.91 − 1.53)	0.2124	1.39 (0.93 − 2.07)	0.1089	1.05 (0.74 − 1.47)	0.7960
PC aa C38:3	1.01 (0.72 − 1.41)	0.9552	1.05 (0.63 − 1.77)	0.8432	0.98 (0.63 − 1.52)	0.9258
PC aa C38:4	1.26 (0.93 − 1.72)	0.1394	1.22 (0.75 − 1.99)	0.4203	1.29 (0.87 − 1.93)	0.2109
PC aa C38:5	1.33 (1.00 − 1.78)	0.0494*	1.35 (0.87 − 2.08)	0.1761	1.32 (0.90 − 1.94)	0.1535
PC aa C38:6	1.13 (0.88 − 1.45)	0.3399	1.13 (0.77 − 1.66)	0.5420	1.13 (0.82 − 1.57)	0.4628
PC aa C40:1	1.01 (0.76 − 1.35)	0.9344	1.29 (0.82 − 2.03)	0.2792	0.86 (0.58 − 1.25)	0.4228
PC aa C40:3	1.34 (0.99 − 1.82)	0.0572	1.53 (0.96 − 2.46)	0.0760	1.22 (0.82 − 1.82)	0.3264
PC aa C40:4	1.01 (0.79 − 1.31)	0.9135	1.03 (0.69 − 1.52)	0.9037	1.01 (0.72 − 1.41)	0.9685
PC aa C40:5	1.14 (0.87 − 1.50)	0.3300	1.00 (0.66 − 1.52)	0.9954	1.26 (0.88 − 1.80)	0.2037
PC aa C40:6	1.05 (0.83 − 1.33)	0.6909	0.97 (0.67 − 1.39)	0.8631	1.11 (0.82 − 1.51)	0.5048
PC aa C42:0	1.15 (0.89 − 1.48)	0.2928	1.38 (0.92 − 2.06)	0.1206	1.01 (0.73 − 1.41)	0.9423
PC aa C42:1	0.97 (0.75 − 1.24)	0.7885	1.09 (0.76 − 1.57)	0.6389	0.87 (0.62 − 1.22)	0.4198
PC aa C42:4	1.29 (1.00 − 1.68)	0.0546	1.34 (0.92 − 1.97)	0.1305	1.25 (0.87 − 1.79)	0.2254
PC aa C42:5	1.17 (0.90 − 1.52)	0.2487	1.32 (0.89 − 1.96)	0.1690	1.06 (0.75 − 1.50)	0.7511

Additional file 3: (Continued)

Metabolite	40-60 years [a]		40-50 years [a]		60 years [a]	
	**OR_{crude} (95% CI)**	**p_{crude} [b]**	**OR_{crude} (95% CI)**	**p_{crude} [b]**	**OR_{crude} (95% CI)**	**p_{crude} [b]**
PC ae C30:2	0.98 (0.78 − 1.22)	0.8409	0.91 (0.65 − 1.28)	0.5746	1.04 (0.77 − 1.40)	0.8198
PC ae C34:0	1.13 (0.86 − 1.48)	0.3797	0.89 (0.58 − 1.37)	0.6025	1.33 (0.93 − 1.89)	0.1183
PC ae C34:1	1.11 (0.78 − 1.57)	0.5552	0.92 (0.53 − 1.58)	0.7487	1.27 (0.81 − 1.99)	0.3038
PC ae C34:2	1.05 (0.79 − 1.40)	0.7440	0.98 (0.64 − 1.50)	0.9201	1.11 (0.76 − 1.63)	0.5959
PC ae C34:3	1.20 (0.94 − 1.54)	0.1475	1.19 (0.81 − 1.74)	0.3803	1.22 (0.87 − 1.69)	0.2477
PC ae C36:1	1.21 (0.88 − 1.66)	0.2353	0.91 (0.56 − 1.50)	0.7218	1.48 (0.97 − 2.24)	0.0664
PC ae C36:2	1.38 (0.98 − 1.93)	0.0645	1.00 (0.59 − 1.69)	0.9879	1.72 (1.10 − 2.68)	0.0167*
PC ae C36:3	1.16 (0.84 − 1.61)	0.3622	1.23 (0.75 − 2.01)	0.4179	1.12 (0.73 − 1.72)	0.6150
PC ae C36:4	1.18 (0.88 − 1.59)	0.2730	1.25 (0.81 − 1.94)	0.3153	1.12 (0.75 − 1.68)	0.5701
PC ae C36:5	1.30 (1.00 − 1.68)	0.0478*	1.27 (0.86 − 1.86)	0.2306	1.32 (0.94 − 1.87)	0.1134
PC ae C38:0	1.24 (0.97 − 1.59)	0.0925	1.30 (0.88 − 1.93)	0.1861	1.20 (0.87 − 1.65)	0.2753
PC ae C38:1	1.02 (0.95 − 1.10)	0.6020	1.02 (0.92 − 1.14)	0.7033	1.02 (0.92 − 1.13)	0.7200
PC ae C38:2	1.16 (0.89 − 1.50)	0.2741	1.20 (0.80 − 1.79)	0.3718	1.13 (0.80 − 1.60)	0.5002
PC ae C38:3	1.35 (0.97 − 1.87)	0.0765	1.18 (0.72 − 1.94)	0.5125	1.49 (0.96 − 2.30)	0.0759
PC ae C38:4	1.47 (1.04 − 2.07)	0.0303*	1.41 (0.82 − 2.41)	0.2117	1.51 (0.96 − 2.37)	0.0752
PC ae C38:5	1.29 (0.91 − 1.82)	0.1521	1.43 (0.85 − 2.39)	0.1744	1.18 (0.74 − 1.88)	0.4853
PC ae C38:6	1.21 (0.93 − 1.58)	0.1640	1.29 (0.87 − 1.91)	0.2127	1.15 (0.80 − 1.66)	0.4599
PC ae C40:0	1.19 (0.89 − 1.58)	0.2392	1.19 (0.76 − 1.85)	0.4451	1.19 (0.82 − 1.73)	0.3704
PC ae C40:1	1.44 (1.06 − 1.96)	0.0189*	1.29 (0.80 − 2.08)	0.2917	1.56 (1.04 − 2.32)	0.0298*
PC ae C40:2	1.33 (0.98 − 1.81)	0.0676	1.14 (0.71 − 1.83)	0.5827	1.49 (0.99 − 2.23)	0.0539
PC ae C40:3	1.17 (0.86 − 1.59)	0.3163	1.12 (0.73 − 1.73)	0.5971	1.22 (0.79 − 1.90)	0.3726
PC ae C40:5	1.44 (1.01 − 2.05)	0.0425*	1.73 (1.03 − 2.90)	0.0385*	1.22 (0.75 − 1.99)	0.4213
PC ae C40:6	1.33 (0.99 − 1.79)	0.0583	1.23 (0.78 − 1.93)	0.3721	1.42 (0.96 − 2.11)	0.0831
PC ae C42:1	1.08 (0.80 − 1.46)	0.6146	1.08 (0.67 − 1.72)	0.7588	1.08 (0.73 − 1.61)	0.6894
PC ae C42:2	1.35 (0.99 − 1.86)	0.0595	1.35 (0.83 − 2.21)	0.2281	1.36 (0.90 − 2.05)	0.1476
PC ae C42:3	1.32 (0.95 − 1.84)	0.0965	1.32 (0.78 − 2.21)	0.2994	1.32 (0.87 − 2.02)	0.1940
PC ae C42:4	1.35 (0.99 − 1.84)	0.0580	1.37 (0.87 − 2.15)	0.1719	1.33 (0.87 − 2.03)	0.1879
PC ae C42:5	1.20 (0.84 − 1.69)	0.3153	1.47 (0.87 − 2.46)	0.1479	1.01 (0.63 − 1.62)	0.9769
PC ae C44:3	0.99 (0.79 − 1.25)	0.9517	1.05 (0.74 − 1.49)	0.7823	0.95 (0.69 − 1.30)	0.7432
PC ae C44:4	1.10 (0.82 − 1.47)	0.5168	1.09 (0.71 − 1.68)	0.6993	1.11 (0.75 − 1.63)	0.6003
PC ae C44:5	1.00 (0.75 − 1.32)	0.9731	1.04 (0.67 − 1.62)	0.8594	0.96 (0.66 − 1.40)	0.8471
PC ae C44:6	1.03 (0.77 − 1.37)	0.8481	1.09 (0.71 − 1.66)	0.7091	0.99 (0.68 − 1.44)	0.9404
LPC C16:0	1.50 (1.09 − 2.07)	0.0122*	1.36 (0.83 − 2.22)	0.2252	1.62 (1.06 − 2.45)	0.0243*
LPC C16:1	1.08 (0.84 − 1.38)	0.5675	0.91 (0.63 − 1.32)	0.6318	1.23 (0.88 − 1.73)	0.2244
LPC C17:0	1.59 (1.21 − 2.08)	0.0007*	1.10 (0.73 − 1.66)	0.6567	2.08 (1.45 − 2.98)	<0.0001*
LPC C18:0	1.59 (1.17 − 2.18)	0.0034*	1.31 (0.81 − 2.12)	0.2791	1.83 (1.22 − 2.75)	0.0037*
LPC C18:1	1.39 (1.04 − 1.87)	0.0270*	1.22 (0.78 − 1.90)	0.3924	1.54 (1.05 − 2.28)	0.0293*
LPC C18:2	1.19 (0.94 − 1.50)	0.1486	1.05 (0.74 − 1.50)	0.7705	1.31 (0.96 − 1.80)	0.0943
LPC C20:3	1.30 (0.99 − 1.70)	0.0587	1.18 (0.77 − 1.82)	0.4484	1.38 (0.97 − 1.95)	0.0699
LPC C20:4	1.43 (1.09 − 1.86)	0.0085*	1.25 (0.83 − 1.86)	0.2854	1.58 (1.11 − 2.24)	0.0109*
LPC C24:0	0.92 (0.77 − 1.10)	0.3511	0.94 (0.70 − 1.27)	0.7031	0.90 (0.72 − 1.13)	0.3766
SM-OH C14:1	1.11 (0.82 − 1.50)	0.5039	0.99 (0.62 − 1.58)	0.9669	1.21 (0.81 − 1.80)	0.3585
SM-OH C16:1	1.12 (0.82 − 1.51)	0.4835	0.97 (0.60 − 1.56)	0.8842	1.23 (0.83 − 1.83)	0.3046
SM-OH C22:1	1.19 (0.88 − 1.62)	0.2639	1.16 (0.73 − 1.84)	0.5203	1.22 (0.80 − 1.84)	0.3543
SM C16:0	1.16 (0.81 − 1.67)	0.4180	1.39 (0.79 − 2.46)	0.2568	1.03 (0.64 − 1.65)	0.9164
SM C16:1	1.16 (0.80 − 1.68)	0.4436	1.21 (0.66 − 2.22)	0.5423	1.13 (0.7 0− 1.81)	0.6196
SM C18:0	1.04 (0.74 − 1.47)	0.8054	1.03 (0.60 − 1.75)	0.9226	1.06 (0.68 − 1.65)	0.8107
SM C18:1	1.05 (0.76 − 1.45)	0.7773	1.10 (0.65 − 1.85)	0.7314	1.02 (0.67 − 1.55)	0.9299
SM C24:1	1.23 (0.87 − 1.74)	0.2347	1.55 (0.91 − 2.65)	0.1062	1.04 (0.66 − 1.64)	0.8633
H1	1.45 (0.90 − 2.33)	0.1255	1.47 (0.71 − 3.05)	0.3026	1.44 (0.77 − 2.69)	0.2568

Additional file 3: (Continued)

Metabolite	40-60 years [a]		40-50 years [a]		60 years [a]	
	OR_{crude} (95% CI)	p_{crude} [b]	OR_{crude} (95% CI)	p_{crude} [b]	OR_{crude} (95% CI)	p_{crude} [b]
**Metabolites measured with NMR and overall prostate cancer risk [a]**						
2-Aminobutyrate	0.82 (0.60 − 1.11)	0.2014	0.75 (0.47 − 1.18)	0.2119	0.88 (0.58 − 1.34)	0.5560
2-Hydroxybutyrate	0.94 (0.75 − 1.17)	0.5695	0.87 (0.63 − 1.22)	0.4290	0.99 (0.73 − 1.34)	0.9610
2-Hydroxyisovalerate	0.98 (0.80 − 1.20)	0.8564	1.01 (0.75 − 1.37)	0.9439	0.96 (0.73 − 1.26)	0.7596
2-Ketoglutarate	0.87 (0.55 − 1.39)	0.5650	0.63 (0.33 − 1.24)	0.1823	1.19 (0.62 − 2.29)	0.6077
2-Oxoisocaproate	0.84 (0.61 − 1.14)	0.2606	0.80 (0.52 − 1.24)	0.3237	0.87 (0.56 − 1.36)	0.5465
3-Hydroxybutyrate	1.00 (0.92 − 1.10)	0.9465	1.03 (0.90 − 1.18)	0.6712	0.98 (0.87 − 1.11)	0.7676
3-Hydroxyisovalerate	0.79 (0.56 − 1.11)	0.1652	0.81 (0.48 − 1.37)	0.4336	0.77 (0.49 − 1.20)	0.2476
Acetate	1.09 (0.92 − 1.28)	0.3203	1.14 (0.91 − 1.43)	0.2669	1.03 (0.81 − 1.32)	0.7832
Acetoacetate	0.95 (0.88 − 1.04)	0.2576	0.98 (0.87 − 1.12)	0.7983	0.93 (0.84 − 1.04)	0.2052
Acetylcarnitine	1.08 (0.84 − 1.40)	0.5408	1.10 (0.75 − 1.62)	0.6152	1.07 (0.76 − 1.50)	0.7102
Alanine	0.86 (0.63 − 1.19)	0.3608	0.82 (0.51 − 1.31)	0.4023	0.90 (0.59 − 1.39)	0.6365
Arginine	0.85 (0.50 − 1.45)	0.5547	0.70 (0.33 − 1.50)	0.3586	1.03 (0.49 − 2.14)	0.9447
Asparagine	0.89 (0.53 − 1.49)	0.6458	1.24 (0.57 − 2.68)	0.5862	0.67 (0.33 − 1.36)	0.2636
Betaine	1.08 (0.65 − 1.80)	0.7658	0.65 (0.30 − 1.41)	0.2753	1.61 (0.81 − 3.20)	0.1724
Carnitine	0.99 (0.67 − 1.47)	0.9774	1.18 (0.63 − 2.22)	0.6051	0.89 (0.54 − 1.47)	0.6529
Choline	1.21 (0.79 − 1.85)	0.3886	0.88 (0.47 − 1.64)	0.6792	1.60 (0.89 − 2.87)	0.1177
Citrate	1.35 (0.95 − 1.91)	0.0914	1.54 (0.92 − 2.57)	0.1010	1.21 (0.75 − 1.93)	0.4372
Creatine	0.89 (0.77 − 1.04)	0.1468	0.88 (0.69 − 1.13)	0.3228	0.90 (0.74 − 1.09)	0.2853
Creatinine	1.10 (0.69 − 1.73)	0.6925	1.30 (0.59 − 2.83)	0.5172	1.01 (0.57 − 1.77)	0.9850
Dimethylglycine	1.10 (0.83 − 1.46)	0.4932	1.00 (0.64 − 1.57)	0.9909	1.18 (0.82 − 1.70)	0.3823
Dimethylsulfone	1.07 (0.88 − 1.30)	0.4904	1.32 (0.98 − 1.79)	0.0701	0.92 (0.71 − 1.19)	0.5172
Formate	1.25 (0.83 − 1.88)	0.2831	0.97 (0.53 − 1.77)	0.9168	1.53 (0.88 − 2.66)	0.1291
Glucose	1.18 (0.69 − 2.02)	0.5375	1.05 (0.44 − 2.47)	0.9179	1.28 (0.65 − 2.53)	0.4799
Glutamate	0.97 (0.80 − 1.17)	0.7524	0.91 (0.69 − 1.20)	0.4898	1.03 (0.79 − 1.34)	0.8231
Glutamine	0.64 (0.37 − 1.12)	0.1154	0.97 (0.47 − 2.01)	0.9259	0.41 (0.18 − 0.90)	0.0267*
Glycine	1.17 (0.81 − 1.69)	0.3943	2.11 (1.21 − 3.69)	0.0084*	0.68 (0.41 − 1.14)	0.1472
Histidine	0.59 (0.32 − 1.08)	0.0884	1.08 (0.45 − 2.63)	0.8599	0.35 (0.15 − 0.81)	0.0136*
Isocaproate	1.81 (0.57 − 5.74)	0.3139	1.30 (0.24 − 7.14)	0.7608	2.39 (0.50 − 11.4)	0.2770
Isoleucine	0.95 (0.62 − 1.47)	0.8314	0.86 (0.46 − 1.64)	0.6551	1.04 (0.58 − 1.85)	0.9058
Lactate	0.96 (0.78 − 1.20)	0.7290	0.97 (0.72 − 1.32)	0.8488	0.95 (0.70 − 1.30)	0.7643
Leucine	0.92 (0.57 − 1.49)	0.7330	0.96 (0.47 − 1.97)	0.9062	0.89 (0.46 − 1.72)	0.7231
Lysine	0.70 (0.43 − 1.13)	0.1454	0.70 (0.34 − 1.44)	0.3325	0.70 (0.37 − 1.34)	0.2772
Methionine	0.66 (0.38 − 1.13)	0.1289	0.71 (0.32 − 1.61)	0.4138	0.62 (0.30 − 1.28)	0.1918
Myo-inositol	1.08 (0.76 − 1.54)	0.6535	1.21 (0.69 − 2.10)	0.5083	1.01 (0.64 − 1.59)	0.9711
O-phosphocholine	0.81 (0.64 − 1.01)	0.0660	0.81 (0.58 − 1.13)	0.2120	0.80 (0.58 − 1.11)	0.1766
Ornithine	0.89 (0.64 − 1.24)	0.4971	1.34 (0.81 − 2.20)	0.2554	0.64 (0.41 − 1.01)	0.0542
Phenylalanine	0.70 (0.40 − 1.23)	0.2151	0.90 (0.37 − 2.15)	0.8073	0.58 (0.28 − 1.23)	0.1567
Proline	0.87 (0.64 − 1.19)	0.3778	0.95 (0.60 − 1.52)	0.8432	0.81 (0.53 − 1.22)	0.3154
Pyruvate	0.79 (0.65 − 0.96)	0.0185*	0.65 (0.48 − 0.89)	0.0066*	0.94 (0.71 − 1.24)	0.6512
Serine	0.84 (0.56 − 1.24)	0.3678	1.13 (0.63 − 2.03)	0.6744	0.65 (0.38 − 1.10)	0.1104
Threonine	0.84 (0.53 − 1.33)	0.4495	1.04 (0.51 − 2.09)	0.9223	0.71 (0.38 − 1.32)	0.2747
Trimethylamine	1.01 (0.82 − 1.25)	0.8986	1.17 (0.85 − 1.61)	0.3416	0.92 (0.70 − 1.20)	0.5219
Trimethylamine-N-oxide	1.01 (0.82 − 1.26)	0.8998	0.99 (0.75 − 1.33)	0.9661	1.04 (0.75 − 1.44)	0.8130
Tyrosine	0.74 (0.48 − 1.14)	0.1720	0.81 (0.41 − 1.59)	0.5405	0.69 (0.39 − 1.22)	0.2045
Valine	0.90 (0.54 − 1.50)	0.6819	0.90 (0.42 − 1.92)	0.7919	0.90 (0.45 − 1.79)	0.7534

Additional file 3: (Continued)

Metabolite	40-60 years [a]		40-50 years [a]		60 years [a]	
	OR_{crude} (95% CI)	p_{crude} [b]	OR_{crude} (95% CI)	p_{crude} [b]	OR_{crude} (95% CI)	p_{crude} [b]
**Metabolites measured by MS and non-aggressive prostate cancer risk [a]**						
C0	1.24 (0.83 − 1.85)	0.2934	1.20 (0.66 − 2.16)	0.5494	1.28 (0.74 − 2.21)	0.3792
C2	1.13 (0.88 − 1.44)	0.3324	1.02 (0.71 − 1.46)	0.9232	1.24 (0.88 − 1.73)	0.2177
C3	1.27 (0.96 − 1.69)	0.0900	1.01 (0.67 − 1.52)	0.9646	1.57 (1.06 − 2.33)	0.0231*
C4	0.92 (0.74 − 1.15)	0.4662	0.78 (0.56 − 1.09)	0.1491	1.06 (0.78 − 1.43)	0.7266
C5	1.09 (0.80 − 1.48)	0.5953	0.96 (0.60 − 1.53)	0.8690	1.19 (0.79 − 1.80)	0.3951
C10	1.07 (0.87 − 1.32)	0.5264	0.99 (0.73 − 1.34)	0.9313	1.15 (0.86 − 1.53)	0.3460
C16	1.09 (0.76 − 1.57)	0.6325	1.09 (0.64 − 1.86)	0.7469	1.09 (0.67 − 1.80)	0.7241
C18:1	1.00 (0.73 − 1.37)	0.9900	1.11 (0.72 − 1.72)	0.6310	0.90 (0.57 − 1.41)	0.6301
C18:2	1.01 (0.78 − 1.32)	0.9197	0.98 (0.67 − 1.42)	0.8991	1.05 (0.72 − 1.54)	0.7874
Ala	0.93 (0.68 − 1.28)	0.6558	0.99 (0.63 − 1.56)	0.9652	0.88 (0.56 − 1.37)	0.5606
Arg	0.73 (0.54 − 1.00)	0.0494*	0.61 (0.39 − 0.96)	0.0306*	0.87 (0.57 − 1.34)	0.5321
His	0.78 (0.47 − 1.31)	0.3487	1.07 (0.54 − 2.13)	0.8565	0.53 (0.25 − 1.15)	0.1105
Ile	0.97 (0.63 − 1.50)	0.8826	0.89 (0.48 − 1.63)	0.6990	1.06 (0.57 − 1.98)	0.8529
Leu	1.00 (0.64 − 1.57)	0.9930	1.01 (0.51 − 1.97)	0.9839	1.00 (0.54 − 1.83)	0.9949
Lys	0.76 (0.51 − 1.13)	0.1719	0.90 (0.50 − 1.62)	0.7264	0.65 (0.37 − 1.13)	0.1233
Orn	0.99 (0.72 − 1.35)	0.9329	1.41 (0.88 − 2.25)	0.1503	0.73 (0.47 − 1.12)	0.1503
Phe	0.99 (0.59 − 1.65)	0.9621	0.94 (0.45 − 1.98)	0.8743	1.03 (0.51 − 2.09)	0.9319
Pro	1.09 (0.79 − 1.51)	0.6019	1.23 (0.76 − 2.00)	0.3964	0.99 (0.64 − 1.52)	0.9510
Trp	1.18 (0.79 − 1.75)	0.4268	1.13 (0.64 − 2.01)	0.6647	1.21 (0.70 − 2.11)	0.4924
Tyr	0.95 (0.65 − 1.38)	0.7768	0.84 (0.49 − 1.44)	0.5266	1.06 (0.63 − 1.80)	0.8212
Creatinine	1.35 (0.77 − 2.37)	0.2944	1.36 (0.57 − 3.23)	0.4882	1.34 (0.64 − 2.81)	0.4318
Spermidine	0.99 (0.84 − 1.17)	0.9118	0.92 (0.72 − 1.16)	0.4676	1.08 (0.84 − 1.38)	0.5551
Taurine	1.20 (0.85 − 1.71)	0.2969	0.89 (0.52 − 1.50)	0.6514	1.53 (0.96 − 2.46)	0.0760
PC aa C28:1	0.94 (0.67 − 1.32)	0.7120	0.86 (0.54 − 1.39)	0.5451	1.03 (0.62 − 1.69)	0.9174
PC aa C30:0	1.03 (0.79 − 1.33)	0.8523	1.06 (0.73 − 1.54)	0.7716	1.00 (0.69 − 1.43)	0.9835
PC aa C32:0	1.09 (0.74 − 1.61)	0.6625	1.06 (0.6 − 1.90)	0.8328	1.11 (0.66 − 1.88)	0.6912
PC aa C32:1	0.91 (0.75 − 1.09)	0.3011	0.89 (0.68 − 1.16)	0.3911	0.92 (0.71 − 1.20)	0.5452
PC aa C32:2	0.96 (0.79 − 1.16)	0.6423	0.94 (0.72 − 1.22)	0.6244	0.98 (0.74 − 1.30)	0.8751
PC aa C34:1	1.07 (0.74 − 1.56)	0.7178	0.99 (0.56 − 1.74)	0.9720	1.14 (0.69 − 1.89)	0.6057
PC aa C34:2	1.23 (0.83 − 1.83)	0.2959	1.09 (0.61 − 1.96)	0.7761	1.36 (0.80 − 2.32)	0.2502
PC aa C34:3	1.07 (0.79 − 1.46)	0.6548	0.99 (0.64 − 1.54)	0.9695	1.16 (0.75 − 1.78)	0.5078
PC aa C34:4	1.11 (0.87 − 1.42)	0.3993	1.02 (0.73 − 1.43)	0.9052	1.23 (0.85 − 1.77)	0.2678
PC aa C36:0	1.20 (0.95 − 1.52)	0.1264	1.17 (0.84 − 1.65)	0.3563	1.23 (0.89 − 1.70)	0.2177
PC aa C36:1	1.02 (0.72 − 1.46)	0.8939	0.93 (0.54 − 1.59)	0.7851	1.10 (0.69 − 1.75)	0.6824
PC aa C36:2	1.13 (0.75 − 1.71)	0.5612	0.99 (0.53 − 1.86)	0.9727	1.25 (0.72 − 2.17)	0.4253
PC aa C36:3	1.09 (0.73 − 1.62)	0.6752	0.97 (0.54 − 1.76)	0.9295	1.19 (0.70 − 2.04)	0.5205
PC aa C36:4	1.34 (0.92 − 1.95)	0.1250	1.31 (0.74 − 2.34)	0.3536	1.36 (0.83 − 2.24)	0.2205
PC aa C36:5	1.19 (0.98 − 1.45)	0.0780	1.24 (0.94 − 1.65)	0.1341	1.15 (0.88 − 1.50)	0.3132
PC aa C36:6	1.19 (0.96 − 1.49)	0.1204	1.11 (0.81 − 1.51)	0.5232	1.28 (0.94 − 1.76)	0.1200
PC aa C38:0	1.33 (0.99 − 1.78)	0.0547	1.36 (0.88 − 2.09)	0.1630	1.31 (0.88 − 1.94)	0.1847
PC aa C38:3	0.94 (0.65 − 1.36)	0.7406	0.98 (0.56 − 1.73)	0.9470	0.91 (0.56 − 1.49)	0.7031
PC aa C38:4	1.21 (0.85 − 1.72)	0.2799	1.24 (0.73 − 2.13)	0.4282	1.19 (0.75 − 1.89)	0.4579
PC aa C38:5	1.28 (0.93 − 1.78)	0.1336	1.23 (0.77 − 1.96)	0.3916	1.34 (0.85 − 2.10)	0.2085
PC aa C38:6	1.20 (0.90 − 1.59)	0.2139	1.05 (0.69 − 1.61)	0.8172	1.32 (0.91 − 1.94)	0.1468
PC aa C40:1	1.09 (0.79 − 1.50)	0.6030	1.35 (0.82 − 2.21)	0.2337	0.93 (0.60 − 1.42)	0.7250
PC aa C40:3	1.42 (1.00 − 2.00)	0.0486*	1.50 (0.91 − 2.48)	0.1141	1.35 (0.83 − 2.17)	0.2247
PC aa C40:4	0.86 (0.65 − 1.16)	0.3240	0.90 (0.59 − 1.38)	0.6263	0.84 (0.56 − 1.24)	0.3719
PC aa C40:5	1.08 (0.80 − 1.47)	0.6173	0.90 (0.57 − 1.41)	0.6372	1.27 (0.84 − 1.93)	0.2652
PC aa C40:6	1.08 (0.82 − 1.40)	0.5958	0.89 (0.60 − 1.32)	0.5664	1.26 (0.88 − 1.80)	0.2178
PC aa C42:0	1.21 (0.91 − 1.61)	0.1900	1.31 (0.86 − 2.02)	0.2141	1.13 (0.77 − 1.66)	0.5197
PC aa C42:1	1.01 (0.77 − 1.33)	0.9271	1.05 (0.71 − 1.55)	0.8005	0.98 (0.66 − 1.44)	0.9012
PC aa C42:4	1.29 (0.96 − 1.73)	0.0878	1.41 (0.93 − 2.15)	0.1079	1.18 (0.78 − 1.79)	0.4227
PC aa C42:5	1.20 (0.89 − 1.63)	0.2254	1.31 (0.85 − 2.01)	0.2167	1.11 (0.73 − 1.69)	0.6311

Additional file 3: (Continued)

Metabolite	40-60 years [a]		40-50 years [a]		60 years [a]	
	**OR_{crude} (95% CI)**	**p_{crude} [b]**	**OR_{crude} (95% CI)**	**p_{crude} [b]**	**OR_{crude} (95% CI)**	**p_{crude} [b]**
PC ae C30:2	0.89 (0.69 − 1.15)	0.3569	0.85 (0.59 − 1.24)	0.4024	0.92 (0.64 − 1.31)	0.6362
PC ae C34:0	1.02 (0.75 − 1.39)	0.9132	0.83 (0.52 − 1.31)	0.4167	1.22 (0.80 − 1.86)	0.3677
PC ae C34:1	1.06 (0.72 − 1.57)	0.7568	0.90 (0.50 − 1.62)	0.7324	1.22 (0.72 − 2.06)	0.4680
PC ae C34:2	1.03 (0.75 − 1.42)	0.8444	0.97 (0.62 − 1.52)	0.8867	1.10 (0.70 − 1.74)	0.6748
PC ae C34:3	1.25 (0.95 − 1.66)	0.1125	1.23 (0.82 − 1.86)	0.3147	1.27 (0.87 − 1.86)	0.2180
PC ae C36:1	1.04 (0.73 − 1.49)	0.8128	0.85 (0.50 − 1.43)	0.5375	1.25 (0.77 − 2.05)	0.3661
PC ae C36:2	1.21 (0.82 − 1.78)	0.3308	0.89 (0.50 − 1.57)	0.6860	1.57 (0.93 − 2.66)	0.0930
PC ae C36:3	1.19 (0.83 − 1.72)	0.3446	1.23 (0.73 − 2.07)	0.4468	1.16 (0.70 − 1.92)	0.5613
PC ae C36:4	1.12 (0.80 − 1.57)	0.5117	1.23 (0.77 − 1.98)	0.3939	1.02 (0.63 − 1.64)	0.9430
PC ae C36:5	1.24 (0.93 − 1.66)	0.1374	1.23 (0.81 − 1.87)	0.3310	1.25 (0.85 − 1.86)	0.2605
PC ae C38:0	1.31 (0.99 − 1.73)	0.0601	1.29 (0.84 − 1.97)	0.2408	1.33 (0.91 − 1.93)	0.1411
PC ae C38:1	0.99 (0.91 − 1.07)	0.7869	0.99 (0.89 − 1.12)	0.9169	0.98 (0.88 − 1.11)	0.7800
PC ae C38:2	1.12 (0.84 − 1.51)	0.4345	1.16 (0.76 − 1.77)	0.5001	1.10 (0.73 − 1.64)	0.6624
PC ae C38:3	1.09 (0.76 − 1.57)	0.6426	1.03 (0.61 − 1.74)	0.9176	1.15 (0.69 − 1.91)	0.5864
PC ae C38:4	1.24 (0.84 − 1.83)	0.2903	1.33 (0.75 − 2.37)	0.3368	1.16 (0.68 − 1.98)	0.5794
PC ae C38:5	1.25 (0.85 − 1.84)	0.2554	1.36 (0.78 − 2.36)	0.2728	1.15 (0.67 − 1.98)	0.6074
PC ae C38:6	1.22 (0.90 − 1.66)	0.1912	1.19 (0.78 − 1.82)	0.4236	1.26 (0.82 − 1.94)	0.2935
PC ae C40:0	1.32 (0.95 − 1.84)	0.1010	1.07 (0.66 − 1.74)	0.7789	1.58 (1.00 − 2.50)	0.0489*
PC ae C40:1	1.54 (1.09 − 2.18)	0.0157*	1.21 (0.73 − 2.01)	0.4687	1.90 (1.17 − 3.09)	0.0093*
PC ae C40:2	1.12 (0.80 − 1.59)	0.5079	1.04 (0.64 − 1.71)	0.8656	1.21 (0.74 − 1.96)	0.4462
PC ae C40:3	1.10 (0.79 − 1.55)	0.5646	1.10 (0.70 − 1.72)	0.6775	1.11 (0.66 − 1.87)	0.6892
PC ae C40:5	1.49 (1.00 − 2.21)	0.0502	1.73 (0.99 − 3.00)	0.0535	1.26 (0.71 − 2.24)	0.4294
PC ae C40:6	1.28 (0.91 − 1.79)	0.1549	1.04 (0.65 − 1.68)	0.8647	1.55 (0.97 − 2.50)	0.0693
PC ae C42:1	1.05 (0.75 − 1.48)	0.7817	1.01 (0.60 − 1.68)	0.9846	1.09 (0.69 − 1.72)	0.7231
PC ae C42:2	1.25 (0.87 − 1.78)	0.2282	1.13 (0.67 − 1.92)	0.6380	1.35 (0.83 − 2.20)	0.2283
PC ae C42:3	1.37 (0.94 − 1.99)	0.1015	1.22 (0.69 − 2.15)	0.4893	1.49 (0.91 − 2.46)	0.1165
PC ae C42:4	1.30 (0.92 − 1.82)	0.1350	1.29 (0.80 − 2.06)	0.2988	1.31 (0.80 − 2.12)	0.2822
PC ae C42:5	1.18 (0.81 − 1.73)	0.3848	1.47 (0.85 − 2.54)	0.1686	0.96 (0.56 − 1.64)	0.8842
PC ae C44:3	1.07 (0.82 − 1.38)	0.6238	1.07 (0.74 − 1.55)	0.7207	1.06 (0.74 − 1.52)	0.7364
PC ae C44:4	1.00 (0.72 − 1.38)	0.9919	0.95 (0.60 − 1.52)	0.8308	1.05 (0.67 − 1.64)	0.8476
PC ae C44:5	0.99 (0.72 − 1.36)	0.9654	1.06 (0.66 − 1.71)	0.7962	0.94 (0.61 − 1.44)	0.7693
PC ae C44:6	1.06 (0.78 − 1.45)	0.7117	1.03 (0.65 − 1.62)	0.9068	1.09 (0.71 − 1.68)	0.6899
LPC C16:0	1.39 (0.97 − 1.99)	0.0764	1.32 (0.77 − 2.26)	0.3073	1.44 (0.89 − 2.34)	0.1426
LPC C16:1	0.97 (0.74 − 1.28)	0.8474	0.87 (0.59 − 1.27)	0.4668	1.10 (0.74 − 1.65)	0.6290
LPC C17:0	1.36 (1.01 − 1.85)	0.0454*	1.03 (0.67 − 1.60)	0.8951	1.77 (1.15 − 2.72)	0.0089*
LPC C18:0	1.49 (1.05 − 2.12)	0.0256*	1.24 (0.73 − 2.09)	0.4280	1.75 (1.08 − 2.81)	0.0225*
LPC C18:1	1.35 (0.97 − 1.87)	0.0787	1.21 (0.75 − 1.94)	0.4349	1.49 (0.94 − 2.37)	0.0909
LPC C18:2	1.13 (0.87 − 1.47)	0.3486	1.02 (0.70 − 1.50)	0.9020	1.24 (0.87 − 1.79)	0.2397
LPC C20:3	1.15 (0.85 − 1.56)	0.3803	1.14 (0.72 − 1.81)	0.5722	1.15 (0.76 − 1.73)	0.5016
LPC C20:4	1.28 (0.95 − 1.74)	0.1095	1.24 (0.81 − 1.92)	0.3256	1.32 (0.86 − 2.02)	0.2017
LPC C24:0	0.91 (0.74 − 1.11)	0.3575	0.92 (0.67 − 1.27)	0.6046	0.90 (0.70 − 1.17)	0.4453
SM-OH C14:1	1.03 (0.74 − 1.45)	0.8493	0.99 (0.61 − 1.62)	0.9671	1.07 (0.68 − 1.71)	0.7640
SM-OH C16:1	1.02 (0.72 − 1.43)	0.9328	0.96 (0.58 − 1.59)	0.8612	1.07 (0.67 − 1.70)	0.7825
SM-OH C22:1	1.06 (0.76 − 1.50)	0.7267	1.10 (0.68 − 1.79)	0.6899	1.02 (0.63 − 1.66)	0.9239
SM C16:0	1.25 (0.83 − 1.88)	0.2782	1.46 (0.80 − 2.66)	0.2167	1.10 (0.63 − 1.91)	0.7414
SM C16:1	1.29 (0.84 − 1.96)	0.2461	1.25 (0.66 − 2.39)	0.4982	1.31 (0.75 − 2.30)	0.3437
SM C18:0	1.13 (0.77 − 1.65)	0.5457	1.11 (0.63 − 1.96)	0.7273	1.14 (0.68 − 1.90)	0.6179
SM C18:1	1.19 (0.83 − 1.72)	0.3442	1.17 (0.67 − 2.06)	0.5780	1.21 (0.75 − 1.95)	0.4421
SM C24:1	1.29 (0.88 − 1.91)	0.1974	1.61 (0.91 − 2.85)	0.1027	1.06 (0.63 − 1.81)	0.8197
H1	1.57 (0.92 − 2.70)	0.1009	1.75 (0.80 − 3.85)	0.1625	1.42 (0.67 − 3.01)	0.3573

Additional file 3: (Continued)

Metabolite	40-60 years [a]		40-50 years [a]		60 years [a]	
	OR_{crude} (95% CI)	p_{crude} [b]	OR_{crude} (95% CI)	p_{crude} [b]	OR_{crude} (95% CI)	p_{crude} [b]
**Metabolites measured with NMR and non-aggressive prostate cancer risk [a]**						
2-Aminobutyrate	0.88 (0.62 − 1.25)	0.4568	0.85 (0.51 − 1.41)	0.5285	0.90 (0.55 − 1.47)	0.6716
2-Hydroxybutyrate	0.90 (0.70 − 1.15)	0.3978	0.90 (0.63 − 1.29)	0.5646	0.90 (0.63 − 1.27)	0.5358
2-Hydroxyisovalerate	0.96 (0.77 − 1.19)	0.6970	1.02 (0.74 − 1.40)	0.9219	0.91 (0.67 − 1.23)	0.5303
2-Ketoglutarate	0.92 (0.55 − 1.55)	0.7583	0.70 (0.34 − 1.42)	0.3166	1.29 (0.59 − 2.80)	0.5224
2-Oxoisocaproate	0.81 (0.57 − 1.15)	0.2418	0.81 (0.50 − 1.31)	0.3879	0.82 (0.49 − 1.35)	0.4290
3-Hydroxybutyrate	1.00 (0.90 − 1.11)	0.9885	1.02 (0.88 − 1.18)	0.8016	0.98 (0.85 − 1.13)	0.7908
3-Hydroxyisovalerate	0.73 (0.49 − 1.09)	0.1210	0.78 (0.45 − 1.36)	0.3876	0.68 (0.39 − 1.20)	0.1837
Acetate	1.05 (0.87 − 1.27)	0.5964	1.07 (0.84 − 1.37)	0.5876	1.03 (0.77 − 1.37)	0.8586
Acetoacetate	0.96 (0.87 − 1.05)	0.3593	0.98 (0.86 − 1.12)	0.7850	0.94 (0.82 − 1.06)	0.3155
Acetylcarnitine	1.11 (0.83 − 1.47)	0.4895	1.05 (0.70 − 1.58)	0.8076	1.15 (0.78 − 1.71)	0.4708
Alanine	0.92 (0.64 − 1.31)	0.6333	0.86 (0.52 − 1.42)	0.5539	0.98 (0.59 − 1.62)	0.9331
Arginine	0.81 (0.45 − 1.46)	0.4749	0.74 (0.33 − 1.68)	0.4748	0.88 (0.37 − 2.09)	0.7775
Asparagine	0.99 (0.56 − 1.78)	0.9802	1.23 (0.54 − 2.79)	0.6216	0.80 (0.35 − 1.83)	0.5944
Betaine	1.15 (0.66 − 2.02)	0.6217	0.61 (0.27 − 1.38)	0.2384	2.17 (0.96 − 4.89)	0.0618
Carnitine	1.25 (0.80 − 1.97)	0.3331	1.16 (0.59 − 2.30)	0.6634	1.33 (0.72 − 2.43)	0.3631
Choline	1.43 (0.89 − 2.31)	0.1440	0.98 (0.52 − 1.88)	0.9565	2.20 (1.08 − 4.47)	0.0290*
Citrate	1.48 (0.99 − 2.20)	0.0565	1.57 (0.89 − 2.77)	0.1209	1.39 (0.79 − 2.44)	0.2522
Creatine	0.93 (0.78 − 1.12)	0.4377	0.87 (0.66 − 1.14)	0.3082	0.98 (0.78 − 1.25)	0.8828
Creatinine	1.17 (0.69 − 1.97)	0.5576	1.24 (0.54 − 2.86)	0.6106	1.13 (0.57 − 2.20)	0.7316
Dimethylglycine	1.01 (0.74 − 1.38)	0.9560	0.90 (0.57 − 1.44)	0.6644	1.10 (0.73 − 1.68)	0.6449
Dimethylsulfone	1.08 (0.87 − 1.35)	0.4976	1.24 (0.89 − 1.73)	0.2093	0.97 (0.72 − 1.30)	0.8287
Formate	1.31 (0.82 − 2.08)	0.2546	1.02 (0.53 − 1.96)	0.9578	1.66 (0.86 − 3.19)	0.1289
Glucose	1.34 (0.72 − 2.49)	0.3512	1.29 (0.51 − 3.24)	0.5862	1.39 (0.60 − 3.19)	0.4435
Glutamate	1.00 (0.81 − 1.23)	1.0000	0.97 (0.73 − 1.29)	0.8419	1.03 (0.76 − 1.40)	0.8329
Glutamine	0.84 (0.47 − 1.50)	0.5523	1.05 (0.50 − 2.23)	0.8949	0.60 (0.24 − 1.50)	0.2722
Glycine	1.32 (0.87 − 1.99)	0.1938	2.01 (1.13 − 3.58)	0.0180*	0.78 (0.41 − 1.46)	0.4271
Histidine	0.77 (0.39 − 1.52)	0.4526	1.22 (0.48 − 3.11)	0.6778	0.47 (0.18 − 1.25)	0.1307
Isocaproate	1.94 (0.54 − 7.04)	0.3133	1.24 (0.20 − 7.73)	0.8168	2.99 (0.49 − 18.3)	0.2360
Isoleucine	0.95 (0.58 − 1.58)	0.8554	0.88 (0.43 − 1.81)	0.7333	1.03 (0.51 − 2.10)	0.9343
Lactate	0.98 (0.77 − 1.25)	0.8765	1.03 (0.75 − 1.43)	0.8408	0.92 (0.65 − 1.31)	0.6517
Leucine	0.92 (0.53 − 1.61)	0.7729	0.95 (0.43 − 2.13)	0.9080	0.89 (0.41 − 1.95)	0.7717
Lysine	0.75 (0.44 − 1.29)	0.3008	0.78 (0.36 − 1.70)	0.5327	0.72 (0.34 − 1.55)	0.4016
Methionine	0.71 (0.38 − 1.33)	0.2852	0.75 (0.31 − 1.81)	0.5203	0.68 (0.28 − 1.63)	0.3868
Myo-inositol	1.06 (0.72 − 1.58)	0.7550	1.09 (0.61 − 1.96)	0.7792	1.05 (0.62 − 1.77)	0.8667
O-phosphocholine	0.80 (0.62 − 1.05)	0.1020	0.77 (0.54 − 1.11)	0.1596	0.84 (0.57 − 1.24)	0.3775
Ornithine	1.07 (0.73 − 1.57)	0.7134	1.48 (0.87 − 2.53)	0.1492	0.76 (0.44 − 1.32)	0.3343
Phenylalanine	0.74 (0.38 − 1.41)	0.3547	1.08 (0.42 − 2.82)	0.8702	0.53 (0.22 − 1.29)	0.1586
Proline	0.92 (0.65 − 1.30)	0.6185	0.97 (0.58 − 1.62)	0.9038	0.87 (0.54 − 1.41)	0.5707
Pyruvate	0.76 (0.61 − 0.95)	0.0173*	0.64 (0.46 − 0.89)	0.0075*	0.94 (0.68 − 1.30)	0.6939
Serine	0.86 (0.55 − 1.34)	0.4933	0.96 (0.51 − 1.81)	0.8971	0.76 (0.41 − 1.44)	0.4022
Threonine	0.95 (0.56 − 1.62)	0.8610	1.11 (0.53 − 2.32)	0.7917	0.82 (0.39 − 1.74)	0.6067
Trimethylamine	0.98 (0.77 − 1.24)	0.8451	1.20 (0.85 − 1.69)	0.3123	0.82 (0.59 − 1.13)	0.2259
Trimethylamine-N-oxide	0.92 (0.73 − 1.17)	0.4946	0.90 (0.67 − 1.22)	0.5072	0.95 (0.65 − 1.38)	0.7888
Tyrosine	0.79 (0.48 − 1.32)	0.3705	0.89 (0.43 − 1.84)	0.7465	0.71 (0.35 − 1.45)	0.3502
Valine	0.92 (0.52 − 1.65)	0.7878	0.88 (0.38 − 2.03)	0.7627	0.97 (0.43 − 2.18)	0.9351

Additional file 3: (Continued)

Metabolite	40-60 years [a]		40-50 years [a]		60 years [a]	
	OR_{crude} (95% CI)	p_{crude} [b]	OR_{crude} (95% CI)	p_{crude} [b]	OR_{crude} (95% CI)	p_{crude} [b]
**Metabolites measured with MS and aggressive prostate cancer risk [a]**						
C0	0.85 (0.41 − 1.76)	0.6596	2.26 (0.54 − 9.51)	0.2663	0.58 (0.24 − 1.40)	0.2262
C2	1.33 (0.80 − 2.21)	0.2738	2.10 (0.80 − 5.54)	0.1339	1.08 (0.58 − 2.00)	0.8113
C3	1.57 (0.94 − 2.63)	0.0867	3.09 (1.10 − 8.71)	0.0328*	1.17 (0.63 − 2.18)	0.6136
C4	1.20 (0.77 − 1.85)	0.4262	2.55 (1.09 − 5.95)	0.0306*	0.81 (0.47 − 1.41)	0.4624
C5	1.53 (0.82 − 2.88)	0.1852	4.20 (1.03 − 17.1)	0.0454*	1.10 (0.53 − 2.28)	0.8075
C10	1.05 (0.72 − 1.54)	0.7989	1.59 (0.79 − 3.19)	0.1934	0.86 (0.53 − 1.37)	0.5183
C16	0.83 (0.43 − 1.62)	0.5850	0.88 (0.26 − 2.98)	0.8321	0.81 (0.37 − 1.80)	0.6076
C18:1	0.67 (0.37 − 1.20)	0.1740	0.73 (0.22 − 2.38)	0.6003	0.65 (0.33 − 1.27)	0.2062
C18:2	0.51 (0.29 − 0.89)	0.0167*	0.44 (0.14 − 1.41)	0.1658	0.53 (0.29 − 1.00)	0.0488*
Ala	1.11 (0.58 − 2.12)	0.7442	0.81 (0.27 − 2.48)	0.7124	1.31 (0.59 − 2.90)	0.5083
Arg	1.46 (0.79 − 2.69)	0.2254	1.01 (0.36 − 2.82)	0.9816	1.78 (0.83 − 3.84)	0.1398
His	0.65 (0.23 − 1.81)	0.4078	0.94 (0.14 − 6.43)	0.9516	0.56 (0.17 − 1.89)	0.3492
Ile	1.37 (0.63 − 2.96)	0.4292	1.78 (0.45 − 7.06)	0.4145	1.21 (0.47 − 3.09)	0.6952
Leu	1.68 (0.69 − 4.11)	0.2556	2.48 (0.51 − 12.0)	0.2584	1.38 (0.46 − 4.14)	0.5645
Lys	0.92 (0.42 − 2.03)	0.8336	0.89 (0.19 − 4.10)	0.8763	0.93 (0.37 − 2.36)	0.8797
Orn	0.71 (0.41 − 1.23)	0.2233	1.18 (0.37 − 3.74)	0.7738	0.61 (0.33 − 1.15)	0.1274
Phe	1.09 (0.40 − 2.93)	0.8681	1.08 (0.13 − 9.31)	0.9416	1.09 (0.36 − 3.32)	0.8815
Pro	1.12 (0.58 − 2.16)	0.7423	1.25 (0.39 − 3.98)	0.7037	1.06 (0.47 − 2.36)	0.8924
Trp	1.03 (0.46 − 2.29)	0.9478	0.65 (0.17 − 2.53)	0.5338	1.32 (0.49 − 3.60)	0.5860
Tyr	0.81 (0.41 − 1.61)	0.5513	0.61 (0.14 − 2.68)	0.5159	0.88 (0.41 − 1.90)	0.7422
Creatinine	1.84 (0.63 − 5.38)	0.2653	2.3 (0.18 − 28.75)	0.5182	1.75 (0.54 − 5.73)	0.3543
Spermidine	1.07 (0.78 − 1.48)	0.6734	0.74 (0.41 − 1.35)	0.3230	1.26 (0.85 − 1.87)	0.2456
Taurine	1.67 (0.90 − 3.10)	0.1034	0.96 (0.30 − 3.04)	0.9383	2.10 (0.99 − 4.46)	0.0548
PC aa C28:1	1.45 (0.70 − 3.03)	0.3221	1.56 (0.30 − 8.04)	0.5954	1.42 (0.63 − 3.24)	0.4002
PC aa C30:0	1.35 (0.80 − 2.30)	0.2655	1.80 (0.63 − 5.14)	0.2747	1.22 (0.66 − 2.27)	0.5319
PC aa C32:0	1.21 (0.56 − 2.62)	0.6280	2.20 (0.49 − 9.85)	0.3038	0.96 (0.39 − 2.39)	0.9340
PC aa C32:1	0.96 (0.66 − 1.39)	0.8242	0.96 (0.51 − 1.84)	0.9116	0.96 (0.61 − 1.51)	0.8464
PC aa C32:2	0.94 (0.65 − 1.35)	0.7386	1.08 (0.55 − 2.11)	0.8245	0.89 (0.57 − 1.37)	0.5891
PC aa C34:1	1.08 (0.52 − 2.23)	0.8418	0.88 (0.23 − 3.41)	0.8549	1.17 (0.49 − 2.78)	0.7237
PC aa C34:2	0.69 (0.33 − 1.45)	0.3256	0.69 (0.17 − 2.85)	0.6039	0.69 (0.29 − 1.66)	0.4038
PC aa C34:3	0.74 (0.39 − 1.38)	0.3437	0.34 (0.08 − 1.42)	0.1392	0.91 (0.45 − 1.85)	0.7919
PC aa C34:4	1.22 (0.74 − 2.01)	0.4478	1.18 (0.49 − 2.83)	0.7145	1.23 (0.67 − 2.27)	0.5026
PC aa C36:0	1.11 (0.69 − 1.78)	0.6642	1.85 (0.80 − 4.30)	0.1504	0.83 (0.46 − 1.51)	0.5451
PC aa C36:1	1.20 (0.59 − 2.43)	0.6217	0.74 (0.18 − 3.05)	0.6756	1.41 (0.62 − 3.20)	0.4168
PC aa C36:2	1.04 (0.48 − 2.24)	0.9197	1.51 (0.35 − 6.40)	0.5795	0.90 (0.36 − 2.22)	0.8141
PC aa C36:3	1.37 (0.61 − 3.12)	0.4472	2.73 (0.46 − 16.2)	0.2691	1.13 (0.45 − 2.86)	0.7993
PC aa C36:4	1.42 (0.71 − 2.85)	0.3219	1.27 (0.37 − 4.36)	0.7098	1.50 (0.65 − 3.49)	0.3452
PC aa C36:5	1.12 (0.78 − 1.61)	0.5286	1.30 (0.64 − 2.62)	0.4708	1.07 (0.70 − 1.62)	0.7642
PC aa C36:6	1.11 (0.73 − 1.70)	0.6205	1.51 (0.67 − 3.38)	0.3192	0.98 (0.59 − 1.63)	0.9513
PC aa C38:0	0.74 (0.41 − 1.31)	0.2977	1.57 (0.53 − 4.64)	0.4108	0.53 (0.26 − 1.07)	0.0782
PC aa C38:3	1.37 (0.64 − 2.93)	0.4227	1.54 (0.41 − 5.73)	0.5194	1.28 (0.50 − 3.28)	0.6010
PC aa C38:4	1.46 (0.75 − 2.84)	0.2648	1.13 (0.36 − 3.58)	0.8359	1.66 (0.73 − 3.76)	0.2249
PC aa C38:5	1.52 (0.83 − 2.78)	0.1754	2.38 (0.73 − 7.75)	0.1515	1.28 (0.63 − 2.61)	0.4952
PC aa C38:6	0.92 (0.54 − 1.57)	0.7626	1.55 (0.62 − 3.92)	0.3517	0.70 (0.36 − 1.36)	0.2934
PC aa C40:1	0.72 (0.36 − 1.45)	0.3584	0.96 (0.29 − 3.23)	0.9500	0.63 (0.27 − 1.47)	0.2844
PC aa C40:3	1.12 (0.60 − 2.11)	0.7264	1.80 (0.46 − 7.11)	0.3990	0.98 (0.48 − 2.00)	0.9554
PC aa C40:4	1.81 (1.03 − 3.19)	0.0396*	2.11 (0.74 − 6.04)	0.1621	1.70 (0.87 − 3.33)	0.1233
PC aa C40:5	1.41 (0.78 − 2.54)	0.2530	2.06 (0.63 − 6.74)	0.2323	1.24 (0.63 − 2.45)	0.5388
PC aa C40:6	0.96 (0.58 − 1.59)	0.8773	1.53 (0.60 − 3.90)	0.3755	0.79 (0.43 − 1.45)	0.4388
PC aa C42:0	0.92 (0.51 − 1.65)	0.7686	1.98 (0.60 − 6.53)	0.2639	0.70 (0.35 − 1.40)	0.3159
PC aa C42:1	0.78 (0.43 − 1.41)	0.4089	1.44 (0.50 − 4.15)	0.5040	0.59 (0.29 − 1.21)	0.1503
PC aa C42:4	1.30 (0.73 − 2.34)	0.3765	1.04 (0.41 − 2.66)	0.9300	1.50 (0.71 − 3.19)	0.2899
PC aa C42:5	1.06 (0.62 − 1.81)	0.8452	1.38 (0.49 − 3.88)	0.5407	0.95 (0.50 − 1.80)	0.8799

Additional file 3: (Continued)

Metabolite	40-60 years [a]		40-50 years [a]		60 years [a]	
	OR_{crude} (95% CI)	p_{crude} [b]	OR_{crude} (95% CI)	p_{crude} [b]	OR_{crude} (95% CI)	p_{crude} [b]
PC ae C30:2	1.37 (0.84 − 2.21)	0.2038	1.29 (0.53 − 3.17)	0.5790	1.40 (0.79 − 2.47)	0.2498
PC ae C34:0	1.62 (0.91 − 2.90)	0.1040	1.59 (0.44 − 5.71)	0.4775	1.63 (0.85 − 3.13)	0.1437
PC ae C34:1	1.31 (0.61 − 2.79)	0.4929	1.00 (0.21 − 4.69)	0.9993	1.42 (0.59 − 3.41)	0.4318
PC ae C34:2	1.12 (0.59 − 2.10)	0.7374	1.07 (0.30 − 3.76)	0.9206	1.13 (0.54 − 2.36)	0.7414
PC ae C34:3	1.02 (0.58 − 1.78)	0.9543	0.91 (0.31 − 2.65)	0.8600	1.06 (0.55 − 2.05)	0.8603
PC ae C36:1	2.16 (1.05 − 4.44)	0.0358*	1.83 (0.36 − 9.23)	0.4629	2.25 (1.01 − 5.04)	0.0482*
PC ae C36:2	2.15 (1.03 − 4.48)	0.0415*	2.11 (0.46 − 9.70)	0.3362	2.16 (0.93 − 5.00)	0.0722
PC ae C36:3	1.06 (0.52 − 2.15)	0.8791	1.22 (0.30 − 5.07)	0.7812	1.01 (0.44 − 2.28)	0.9880
PC ae C36:4	1.41 (0.76 − 2.63)	0.2771	1.39 (0.44 − 4.38)	0.5733	1.42 (0.68 − 2.98)	0.3523
PC ae C36:5	1.54 (0.86 − 2.77)	0.1463	1.46 (0.55 − 3.84)	0.4441	1.59 (0.77 − 3.32)	0.2137
PC ae C38:0	1.01 (0.59 − 1.72)	0.9741	1.38 (0.50 − 3.85)	0.5334	0.90 (0.48 − 1.68)	0.7289
PC ae C38:1	1.18 (0.98 − 1.42)	0.0852	1.37 (0.91 − 2.05)	0.1315	1.13 (0.92 − 1.39)	0.2556
PC ae C38:2	1.30 (0.73 − 2.34)	0.3773	1.62 (0.48 − 5.52)	0.4387	1.22 (0.63 − 2.38)	0.5621
PC ae C38:3	3.29 (1.50 − 7.24)	0.0030*	4.17 (0.76 − 23.0)	0.1005	3.08 (1.26 − 7.49)	0.0133*
PC ae C38:4	2.69 (1.25 − 5.76)	0.0110*	2.04 (0.47 − 8.84)	0.3415	2.97 (1.21 − 7.26)	0.0172*
PC ae C38:5	1.43 (0.67 − 3.05)	0.3569	1.95 (0.47 − 8.10)	0.3609	1.26 (0.51 − 3.10)	0.6176
PC ae C38:6	1.16 (0.65 − 2.08)	0.6155	2.17 (0.70 − 6.74)	0.1821	0.90 (0.45 − 1.81)	0.7693
PC ae C40:0	0.86 (0.48 − 1.54)	0.6040	2.12 (0.66 − 6.79)	0.2078	0.61 (0.30 − 1.23)	0.1642
PC ae C40:1	1.16 (0.62 − 2.19)	0.6427	2.03 (0.53 − 7.74)	0.3003	0.98 (0.48 − 2.03)	0.9605
PC ae C40:2	2.49 (1.25 − 4.97)	0.0095*	3.17 (0.56 − 17.8)	0.1909	2.38 (1.12 − 5.04)	0.0239*
PC ae C40:3	1.55 (0.73 − 3.28)	0.2505	1.51 (0.29 − 7.89)	0.6246	1.56 (0.67 − 3.62)	0.2982
PC ae C40:5	1.28 (0.58 − 2.81)	0.5384	1.75 (0.40 − 7.68)	0.4565	1.13 (0.44 − 2.86)	0.8012
PC ae C40:6	1.55 (0.82 − 2.92)	0.1773	4.26 (1.03 − 17.6)	0.0452*	1.15 (0.56 − 2.36)	0.6997
PC ae C42:1	1.19 (0.63 − 2.25)	0.5859	1.52 (0.47 − 4.89)	0.4817	1.08 (0.50 − 2.30)	0.8521
PC ae C42:2	1.81 (0.92 − 3.54)	0.0842	4.45 (1.04 − 19.1)	0.0448*	1.37 (0.64 − 2.94)	0.4213
PC ae C42:3	1.18 (0.59 − 2.34)	0.6414	1.98 (0.52 − 7.48)	0.3164	0.97 (0.43 − 2.17)	0.9347
PC ae C42:4	1.64 (0.77 − 3.49)	0.1963	2.5 (0.56 − 11.15)	0.2311	1.42 (0.59 − 3.40)	0.4376
PC ae C42:5	1.25 (0.53 − 2.94)	0.6053	1.45 (0.28 − 7.45)	0.6558	1.19 (0.44 − 3.23)	0.7391
PC ae C44:3	0.71 (0.40 − 1.24)	0.2311	0.90 (0.31 − 2.65)	0.8496	0.65 (0.34 − 1.26)	0.2004
PC ae C44:4	1.61 (0.84 − 3.06)	0.1514	2.79 (0.77 − 10.2)	0.1199	1.31 (0.62 − 2.78)	0.4861
PC ae C44:5	1.00 (0.52 − 1.94)	0.9902	0.88 (0.23 − 3.30)	0.8437	1.05 (0.49 − 2.24)	0.8990
PC ae C44:6	0.89 (0.45 − 1.75)	0.7314	1.72 (0.46 − 6.53)	0.4233	0.70 (0.32 − 1.55)	0.3769
LPC C16:0	2.01 (1.01 − 3.99)	0.0475*	1.58 (0.44 − 5.71)	0.4850	2.20 (0.97 − 4.97)	0.0579
LPC C16:1	1.62 (0.92 − 2.86)	0.0970	1.64 (0.45 − 6.02)	0.4532	1.61 (0.86 − 3.04)	0.1387
LPC C17:0	2.67 (1.48 − 4.83)	0.0011*	1.77 (0.53 − 5.94)	0.3579	3.02 (1.52 − 6.01)	0.0016*
LPC C18:0	2.01 (1.02 − 3.94)	0.0426*	1.83 (0.50 − 6.74)	0.3659	2.08 (0.95 − 4.56)	0.0684
LPC C18:1	1.57 (0.84 − 2.95)	0.1611	1.27 (0.34 − 4.79)	0.7216	1.67 (0.81 − 3.41)	0.1624
LPC C18:2	1.44 (0.85 − 2.46)	0.1780	1.25 (0.49 − 3.17)	0.6413	1.54 (0.81 − 2.95)	0.1890
LPC C20:3	2.05 (1.12 − 3.75)	0.0206*	1.53 (0.43 − 5.48)	0.5157	2.21 (1.11 − 4.40)	0.0233*
LPC C20:4	1.99 (1.15 − 3.44)	0.0142*	1.26 (0.43 − 3.65)	0.6755	2.33 (1.22 − 4.45)	0.0105*
LPC C24:0	0.95 (0.64 − 1.42)	0.8035	1.09 (0.52 − 2.29)	0.8156	0.90 (0.56 − 1.45)	0.6546
SM-OH C14:1	1.51 (0.74 − 3.07)	0.2529	0.99 (0.23 − 4.24)	0.9939	1.72 (0.76 − 3.90)	0.1920
SM-OH C16:1	1.60 (0.81 − 3.15)	0.1753	1.05 (0.23 − 4.78)	0.9492	1.77 (0.83 − 3.80)	0.1411
SM-OH C22:1	1.94 (0.95 − 3.98)	0.0701	1.86 (0.43 − 8.10)	0.4110	1.97 (0.87 − 4.47)	0.1063
SM C16:0	0.86 (0.38 − 1.95)	0.7131	0.89 (0.14 − 5.44)	0.8965	0.85 (0.34 − 2.14)	0.7291
SM C16:1	0.80 (0.36 − 1.78)	0.5783	0.92 (0.15 − 5.69)	0.9292	0.77 (0.31 − 1.88)	0.5652
SM C18:0	0.76 (0.35 − 1.67)	0.4946	0.58 (0.12 − 2.85)	0.4987	0.83 (0.34 − 2.07)	0.6914
SM C18:1	0.62 (0.29 − 1.30)	0.2051	0.70 (0.16 − 2.99)	0.6277	0.59 (0.25 − 1.41)	0.2362
SM C24:1	1.04 (0.49 − 2.20)	0.9294	1.21 (0.27 − 5.40)	0.8033	0.98 (0.41 − 2.36)	0.9651
H1	1.09 (0.40 − 2.98)	0.8669	0.31 (0.03 − 3.33)	0.3360	1.47 (0.47 − 4.58)	0.5070

Additional file 3: (Continued)

Metabolite	40-60 years [a]		40-50 years [a]		60 years [a]	
	OR_{crude} (95% CI)	p_{crude} [b]	OR_{crude} (95% CI)	p_{crude} [b]	OR_{crude} (95% CI)	p_{crude} [b]
**Metabolites measured with NMR and aggressive prostate cancer risk [a]**						
2-Aminobutyrate	0.66 (0.35 − 1.25)	0.1999	0.39 (0.12 − 1.27)	0.1177	0.84 (0.39 − 1.84)	0.6668
2-Hydroxybutyrate	1.10 (0.68 − 1.79)	0.6942	0.75 (0.32 − 1.76)	0.5105	1.33 (0.73 − 2.42)	0.3451
2-Hydroxyisovalerate	1.12 (0.68 − 1.84)	0.6630	0.97 (0.40 − 2.36)	0.9485	1.19 (0.65 − 2.18)	0.5686
2-Ketoglutarate	0.70 (0.25 − 1.98)	0.5043	0.33 (0.05 − 2.34)	0.2647	0.97 (0.28 − 3.32)	0.9563
2-Oxoisocaproate	0.94 (0.48 − 1.86)	0.8571	0.77 (0.26 − 2.24)	0.6281	1.08 (0.44 − 2.64)	0.8619
3-Hydroxybutyrate	1.02 (0.83 − 1.26)	0.8557	1.11 (0.76 − 1.62)	0.6013	0.98 (0.77 − 1.26)	0.8954
3-Hydroxyisovalerate	0.97 (0.49 − 1.93)	0.9350	1.09 (0.22 − 5.43)	0.9163	0.95 (0.44 − 2.02)	0.8888
Acetate	1.24 (0.86 − 1.78)	0.2530	1.82 (0.90 − 3.66)	0.0934	1.05 (0.68 − 1.63)	0.8187
Acetoacetate	0.94 (0.78 − 1.13)	0.4847	1.00 (0.66 − 1.52)	0.9891	0.92 (0.75 − 1.13)	0.4317
Acetylcarnitine	0.99 (0.55 − 1.79)	0.9774	1.57 (0.51 − 4.83)	0.4346	0.83 (0.41 − 1.67)	0.5936
Alanine	0.67 (0.32 − 1.38)	0.2740	0.58 (0.15 − 2.22)	0.4286	0.71 (0.30 − 1.67)	0.4295
Arginine	1.07 (0.33 − 3.45)	0.9146	0.47 (0.06 − 4.02)	0.4891	1.54 (0.37 − 6.39)	0.5501
Asparagine	0.56 (0.17 − 1.80)	0.3278	1.32 (0.13 − 13.4)	0.8129	0.41 (0.10 − 1.63)	0.2053
Betaine	0.80 (0.24 − 2.69)	0.7206	1.37 (0.07 − 28.3)	0.8384	0.72 (0.19 − 2.72)	0.6316
Carnitine	0.48 (0.21 − 1.10)	0.0821	1.29 (0.25 − 6.69)	0.7641	0.35 (0.13 − 0.92)	0.0340*
Choline	0.59 (0.22 − 1.59)	0.2973	0.20 (0.02 − 2.24)	0.1909	0.75 (0.25 − 2.20)	0.5968
Citrate	1.02 (0.51 − 2.05)	0.9576	1.40 (0.43 − 4.59)	0.5784	0.86 (0.36 − 2.05)	0.7312
Creatine	0.80 (0.59 − 1.07)	0.1359	0.95 (0.54 − 1.68)	0.8680	0.75 (0.53 − 1.06)	0.1031
Creatinine	0.89 (0.34 − 2.29)	0.8058	1.76 (0.18 − 16.9)	0.6246	0.77 (0.27 − 2.19)	0.6168
Dimethylglycine	1.66 (0.85 − 3.26)	0.1406	3.16 (0.63 − 16.0)	0.1633	1.43 (0.68 − 3.01)	0.3415
Dimethylsulfone	1.04 (0.69 − 1.57)	0.8458	1.84 (0.85 − 3.94)	0.1198	0.79 (0.47 − 1.32)	0.3612
Formate	1.06 (0.45 − 2.51)	0.8914	0.72 (0.15 − 3.59)	0.6904	1.25 (0.44 − 3.51)	0.6734
Glucose	0.80 (0.27 − 2.38)	0.6923	0.15 (0.01 − 2.93)	0.2127	1.09 (0.33 − 3.56)	0.8912
Glutamate	0.85 (0.55 − 1.32)	0.4663	0.44 (0.16 − 1.19)	0.1050	1.02 (0.62 − 1.69)	0.9334
Glutamine	0.15 (0.04 − 0.64)	0.0100*	0.22 (0.01 − 5.30)	0.3510	0.14 (0.03 − 0.69)	0.0161*
Glycine	0.77 (0.35 − 1.70)	0.5176	3.82 (0.46 − 31.6)	0.2138	0.53 (0.22 − 1.31)	0.1689
Histidine	0.19 (0.05 − 0.80)	0.0232*	0.36 (0.02 − 6.55)	0.4868	0.16 (0.03 − 0.82)	0.0279*
Isocaproate	1.36 (0.10 − 18.3)	0.8174	1.77 (0.02 − 182)	0.8088	1.20 (0.05 − 27.9)	0.9088
Isoleucine	0.96 (0.42 − 2.18)	0.9123	0.79 (0.19 − 3.36)	0.7500	1.05 (0.38 − 2.87)	0.9296
Lactate	0.88 (0.53 − 1.47)	0.6249	0.56 (0.21 − 1.52)	0.2496	1.06 (0.57 − 1.97)	0.8528
Leucine	0.91 (0.34 − 2.43)	0.8552	0.97 (0.19 − 4.98)	0.9744	0.88 (0.26 − 2.99)	0.8383
Lysine	0.54 (0.19 − 1.54)	0.2472	0.34 (0.05 − 2.58)	0.2965	0.64 (0.19 − 2.20)	0.4784
Methionine	0.50 (0.16 − 1.55)	0.2312	0.54 (0.07 − 4.43)	0.5666	0.49 (0.13 − 1.85)	0.2920
Myo-inositol	1.17 (0.53 − 2.60)	0.7020	2.85 (0.52 − 16.2)	0.2386	0.90 (0.37 − 2.24)	0.8280
O-phosphocholine	0.81 (0.50 − 1.32)	0.4006	1.07 (0.45 − 2.56)	0.8766	0.71 (0.39 − 1.29)	0.2668
Ornithine	0.48 (0.24 − 0.98)	0.0429*	0.62 (0.14 − 2.68)	0.5196	0.45 (0.20 − 1.01)	0.0518
Phenylalanine	0.59 (0.19 − 1.90)	0.3798	0.34 (0.04 − 3.10)	0.3361	0.74 (0.19 − 2.91)	0.6680
Proline	0.73 (0.37 − 1.41)	0.3472	0.89 (0.30 − 2.68)	0.8351	0.65 (0.28 − 1.49)	0.3094
Pyruvate	0.91 (0.58 − 1.43)	0.6712	0.79 (0.30 − 2.08)	0.6272	0.94 (0.57 − 1.57)	0.8231
Serine	0.77 (0.34 − 1.74)	0.5335	2.90 (0.60 − 14.1)	0.1875	0.44 (0.16 − 1.18)	0.1027
Threonine	0.53 (0.20 − 1.42)	0.2066	0.59 (0.07 − 5.28)	0.6366	0.51 (0.17 − 1.56)	0.2398
Trimethylamine	1.15 (0.74 − 1.80)	0.5250	1.01 (0.42 − 2.43)	0.9781	1.21 (0.72 − 2.02)	0.4723
Trimethylamine-N-oxide	1.69 (0.97 − 2.94)	0.0663	2.74 (0.95 − 7.90)	0.0623	1.37 (0.71 − 2.63)	0.3522
Tyrosine	0.60 (0.25 − 1.42)	0.2439	0.44 (0.07 − 2.95)	0.4008	0.65 (0.24 − 1.72)	0.3828
Valine	0.82 (0.29 − 2.38)	0.7180	1.02 (0.17 − 6.13)	0.9810	0.73 (0.20 − 2.73)	0.6421

[a] **Stratifications:**

Overall prostate cancer risk in subjects aged 40-60 years (777/747 pairs; MS/NMR), 40-50 years (333/326; MS/NMR), and 60 years (444/421 pairs; MS/NMR).

Non-aggressive prostate cancer risk in subjects aged 40-60 years (608/586 pairs; MS/NMR), 40-50 years (289/283 pairs; MS/NMR), and 60 years (319/303 pairs; MS/NMR).

Aggressive prostate cancer risk in subjects aged 40-60 years (169/161 pairs; MS/NMR), 40-50 years (44/43 pairs; MS/NMR), and 60 years (125/118 pairs; MS/NMR).

Note that the smaller sample size in specific subgroups results in uncertain risk estimates.

[b] Metabolites with nominal p-value <0.05 are marked with (*).
