# Supplementary material for: Identification of metabolites associated with prostate cancer risk: a nested case-control study with long follow-up in the Northern Sweden Health and Disease Study
Source: BMC Med. 2020 Jul 23;18:187. doi: 10.1186/s12916-020-01655-1 (PMC7376662; doi:10.1186/s12916-020-01655-1)
Supplement: Supplementary file 4 — Additional file 4. Associations significant after correction for multiple testing. [file 12916_2020_1655_MOESM4_ESM.pdf]

Additional file 4: Associations significant after correction for multiple testing

Metabolite [a, b]	Method	Model	1st quartile	2st quartile	3rd quartile	4th quartile
**Overall prostate cancer (40-60 years)**						
LPC C17:0	MS	Cut-point (μM)	≤1.30	>1.30 to ≤1.57	>1.57 to ≤1.89	>1.89
		OR (95% CI)	1.00 (referent)	1.44 (1.07 − 1.96)	1.43 (1.06 − 1.94)	1.63 (1.20 − 2.21)
		P-value	-	0.0178	0.0184	0.0017
		N (cases/controls)	147/195	202/194	202/194	226/194
LPC C18:0	MS	Cut-point (μM)	≤21.6	21.6> to ≤24.7	24.7> to ≤28.9	>28.9
		OR (95% CI)	1.00 (referent)	1.08 (0.81 − 1.45)	1.32 (1.00 − 1.75)	1.36 (1.02 − 1.81)
		P-value	-	0.5947	0.0514	0.0342
		N (cases/controls)	163/195	177/194	216/194	221/194
**Overall prostate cancer (40-50 years)**						
Glycine	NMR	Cut-point (μM)	≤170.1	170.1> to ≤197.2	197.2> to ≤217.7	>217.7
		OR (95% CI)	1.00 (referent)	1.13 (0.70 − 1.83)	1.12 (0.70 − 1.81)	1.75 (1.09 − 2.82)
		P-value	-	0.6216	0.6316	0.0212
		N (cases/controls)	69/82	73/81	74/82	110/81
Pyruvate	NMR	Cut-point (μM)	≤64.5	64.5> to ≤78.8	78.8> to ≤99.5	>99.5
		OR (95% CI)	1.00 (referent)	0.62 (0.40 − 0.97)	1.04 (0.69 − 1.57)	0.46 (0.29 − 0.74)
		P-value	-	0.0374	0.8673	0.0013
		N (cases/controls)	102/82	70/81	105/82	49/81
**Overall prostate cancer (60 years)**						
LPC C17:0*	MS	Cut-point (μM)	≤1.26	>1.26 to ≤1.53	>1.53 to ≤1.84	>1.84
		OR (95% CI)	1.00 (referent)	1.97 (1.29 − 3.01)	2.10 (1.37 − 3.24)	2.56 (1.67 − 3.93)
		P-value	-	0.0016	0.0007	<0.0001
		N (cases/controls)	63/111	116/111	119/111	146/111
LPC C18:0	MS	Cut-point (μM)	≤21.4	21.4> to ≤24.4	24.4> to ≤28.5	>28.5
		OR (95% CI)	1.00 (referent)	0.95 (0.64 − 1.39)	1.30 (0.89 − 1.90)	1.54 (1.07 − 2.22)
		P-value	-	0.7722	0.1820	0.0206
		N (cases/controls)	93/111	87/111	119/111	145/111
**Aggressive prostate cancer (40-60 years)**						
LPC C17:0	MS	Cut-point (μM)	≤1.26	1.26> to ≤1.53	1.53> to ≤1.85	>1.85
		OR (95% CI)	1.00 (referent)	1.64 (0.80 − 3.38)	2.69 (1.29 − 5.60)	3.35 (1.66 − 6.76)
		P-value	-	0.1719	0.0085	0.0008
		N (cases/controls)	22/43	33/42	50/42	64/42
PC ae C38:3	MS	Cut-point (μM)	≤3.39	3.39> to ≤3.92	3. 92> to ≤4.58	>4.58
		OR (95% CI)	1.00 (referent)	1.54 (0.77 − 3.10)	1.98 (0.96 − 4.12)	2.52 (1.28 − 4.97)
		P-value	-	0.2246	0.0665	0.0077
		N (cases/controls)	27/43	37/42	45/42	60/42
PC ae C38:4	MS	Cut-point (μM)	≤9.65	9.65> to ≤11.3	11.3> to ≤13.0	>13.0
		OR (95% CI)	1.00 (referent)	1.32 (0.68 − 2.56)	1.70 (0.85 − 3.39)	2.24 (1.17 − 4.29)
		P-value	-	0.4135	0.1340	0.0147
		N (cases/controls)	29/43	36/42	44/42	60/42

Additional file 4: Continued

Metabolite [a, b]	Method	Model	1st quartile	2st quartile	3rd quartile	4th quartile
**Aggressive prostate cancer (40-60 years)**						
LPC C20:4	MS	Cut-point (μM)	≤3.69	3.69> to ≤4.43	4.43> to ≤5.46	>5.46
		OR (95% CI)	1.00 (referent)	1.24 (0.59 − 2.59)	1.98 (0.98 − 4.02)	2.13 (1.08 − 4.23)
		P-value	-	0.5673	0.0569	0.0300
		N (cases/controls)	30/43	32/42	51/42	56/42
LPC C20:3	MS	Cut-point (μM)	≤1.59	1.59> to ≤1.92	1.92> to ≤2.32	>2.32
		OR (95% CI)	1.00 (referent)	1.41 (0.76 − 2.61)	1.97 (1.03 − 3.77)	1.83 (0.96 − 3.48)
		P-value	-	0.2828	0.0410	0.0652
		N (cases/controls)	28/43	40/42	52/42	49/42
PC ae C40:2	MS	Cut-point (μM)	≤1.78	1.78> to ≤2.06	2.06> to ≤2.42	>2.42
		OR (95% CI)	1.00 (referent)	1.02 (0.54 − 1.94)	1.69 (0.90 − 3.18)	1.85 (1.00 − 3.41)
		P-value	-	0.9413	0.1053	0.0488
		N (cases/controls)	31/43	33/42	50/42	55/42
C18:2	MS	Cut-point (μM)	≤0.035	0.035> to ≤0.042	0.042> to ≤0.051	>0.051
		OR (95% CI)	1.00 (referent)	0.80 (0.46 − 1.40)	0.48 (0.25 − 0.92)	0.55 (0.30 − 1.00)
		P-value	-	0.4212	0.0263	0.0494
		N (cases/controls)	60/43	47/42	30/42	32/42
**Aggressive prostate cancer (60 years)**						
LPC C17:0	MS	Cut-point (μM)	≤1.24	1.24> to ≤1.52	1.52> to ≤1.78	>1.78
		OR (95% CI)	1.00 (referent)	1.63 (0.69 − 3.84)	2.17 (0.92 − 5.15)	3.90 (1.70 − 8.99)
		P-value	-	0.2684	0.0776	0.0014
		N (cases/controls)	16/32	25/31	31/31	53/31

[a] All metabolites listed were significant after correction for multiple testing (FDR, 20%) based on p_{crude}-values in Additional file 3.
[b] Metabolites that were significant after Bonferroni correction based on p_{crude}-values in Additional file 3 are marked with (*).
