# Supplementary material for: Identification of metabolites associated with prostate cancer risk: a nested case-control study with long follow-up in the Northern Sweden Health and Disease Study
Source: BMC Med. 2020 Jul 23;18:187. doi: 10.1186/s12916-020-01655-1 (PMC7376662; doi:10.1186/s12916-020-01655-1)
Supplement: Supplementary file 5 — Additional file 5. Differential associations by age group and disease type. [file 12916_2020_1655_MOESM5_ESM.pdf]

Additional file 5: Differential associations by age group and disease type

Metabolite	Group [a]	OR per doubling	(95% CI)	p_{crude}	N	$p_{interaction}$ [b]
**Stratification by baseline age (overall prostate cancer)**						
LPC C17:0	40-50 years	1.10	(0.73 – 1.66)	0.6567	666	<0.0001*
	60 years*	2.08	(1.45 – 2.98)	<0.0001*	777	
LPC C18:0	40-50 years	1.31	(0.81 – 2.12)	0.2791	666	0.0012*
	60 years	1.83	(1.22 – 2.75)	0.0037*	777	
Glycine	40-50 years	2.11	(1.21 – 3.69)	0.0084*	652	<0.0001*
	60 years	0.68	(0.41 – 1.14)	0.1472	842	
Pyruvate	40-50 years	0.65	(0.48 – 0.89)	0.0066*	652	0.2209
	60 years	0.94	(0.71 – 1.24)	0.6512	842	
**Stratification by disease type (all ages at baseline)**						
LPC C17:0	Non-aggressive	1.36	(1.01 – 1.85)	0.0454	1216	0.0940
	Aggressive	2.67	(1.48 – 4.83)	0.0011*	338	
PC ae C38:3	Non-aggressive	1.09	(0.76 – 1.57)	0.6426	1216	0.0458*
	Aggressive	3.29	(1.50 – 7.24)	0.0030*	338	
PC ae C38:4	Non-aggressive	1.24	(0.84 – 1.83)	0.2903	1216	0.3957
	Aggressive	2.69	(1.25 – 5.76)	0.0110*	338	
LPC C20:4	Non-aggressive	1.28	(0.95 – 1.74)	0.1095	1216	0.7617
	Aggressive	1.99	(1.15 – 3.44)	0.0142*	338	
LPC C20:3	Non-aggressive	1.15	(0.85 – 1.56)	0.3803	1216	0.2258
	Aggressive	2.05	(1.12 – 3.75)	0.0206*	338	
PC ae C40:2	Non-aggressive	1.12	(0.80 – 1.59)	0.5079	1216	0.7486
	Aggressive	2.49	(1.25 – 4.97)	0.0095*	338	
C18:2	Non-aggressive	1.01	(0.78 – 1.32)	0.9197	1216	0.2184
	Aggressive	0.51	(0.29 – 0.89)	0.0167	338	
**Stratification by baseline age (aggressive prostate cancer)**						
LPC C17:0	40-50 years	1.77	(0.53 – 5.94)	0.3579	88	0.0794
	60 years	3.02	(1.52 – 6.01)	0.0016	250	

[a] Listed metabolites were significant after correction for multiple testing (FDR, 20%) according to p-values (p_{crude}) from conditional logistic regression analyses using log2 transformed metabolite data in one of the subgroups (Additional file 3). Associations significant after Bonferroni correction (for 148 independent tests; 0.05/148 = 0.000338) in the log2-based statistical analyses are indicated by (*).

[b] Differential associations by age group or disease type were investigated for each of the listed metabolites using a Wald test, wherein regression coefficients in two subgroups were compared (for age: binary, 40-50, 60 years; for disease: binary, non-aggressive, aggressive). Differential associations were regarded as significant for p-values ($p_{interaction}$) <0.05 as indicated by (*).
