# Supplementary material for: Identification of metabolites associated with prostate cancer risk: a nested case-control study with long follow-up in the Northern Sweden Health and Disease Study
Source: BMC Med. 2020 Jul 23;18:187. doi: 10.1186/s12916-020-01655-1 (PMC7376662; doi:10.1186/s12916-020-01655-1)
Supplement: Supplementary file 6 — Additional file 6. Associations with overall prostate cancer risk after stratification by follow-up time. [file 12916_2020_1655_MOESM6_ESM.pdf]

Additional file 6: Association with overall prostate cancer risk after stratification by follow up-time

Metabolite [a, b]	All subjects (n=1554)		Follow-up ≤10 years (n=800)		Follow-up >10 year (n=754)		Follow-up
	OR (95% CI)	p_{crude}	OR (95% CI)	p_{crude}	OR (95% CI)	p_{crude}	$p_{interaction}$
LPC C17:0	1.59 (1.21 – 2.08)	0.0007**	1.44 (1.00 – 2.07)	0.0501	1.77 (1.20 – 2.63)	0.0044*	0.6952
LPC C18:0	1.59 (1.17 – 2.18)	0.0034**	1.65 (1.08 – 2.54)	0.0216*	1.53 (0.97 – 2.41)	0.0666	0.9260
Glycine	1.17 (0.81 – 1.69)	0.3943	1.26 (0.74 – 2.14)	0.3999	1.10 (0.67 – 1.82)	0.7057	0.1199
Pyruvate	0.79 (0.65 – 0.96)	0.0185*	0.81 (0.60 – 1.11)	0.1844	0.77 (0.60 – 1.00)	0.0515	0.5685
PC ae C38:3	1.35 (0.97 – 1.87)	0.0765	1.10 (0.69 – 1.77)	0.6860	1.62 (1.02 – 2.56)	0.0402*	0.3011
PC ae C38:4	1.47 (1.04 – 2.07)	0.0303*	1.35 (0.82 – 2.20)	0.2370	1.59 (0.98 – 2.59)	0.0613	0.2850
LPC C20:4	1.43 (1.09 – 1.86)	0.0085*	1.42 (1.00 – 2.03)	0.0503	1.43 (0.96 – 2.13)	0.0791	0.3970
LPC C20:3	1.30 (0.99 – 1.70)	0.0587	1.06 (0.75 – 1.51)	0.7418	1.71 (1.12 – 2.61)	0.0125*	0.2139
PC ae C40:2	1.33 (0.98 – 1.81)	0.0676	1.51 (0.97 – 2.34)	0.0660	1.18 (0.77 – 1.81)	0.4554	0.1353
C18:2	0.88 (0.70 – 1.12)	0.3082	0.95 (0.68 – 1.33)	0.7687	0.82 (0.59 – 1.15)	0.2531	0.9954

[a] Only LPC C17:0 and LPC C18:0 were significant (FDR, 20%) when including all subjects (n_{MS} =1554, n_{NMR}=1494), while the other listed metabolites were significant in subgroups stratified by baseline age and/or disease type.

[b] Note that missing NMR data for some subjects resulted in smaller subgroups for glycine and pyruvate ($n_{\leq 10}$ =766, $n_{>10}$ =728).
