# Supplementary material for: Identification of metabolites associated with prostate cancer risk: a nested case-control study with long follow-up in the Northern Sweden Health and Disease Study
Source: BMC Med. 2020 Jul 23;18:187. doi: 10.1186/s12916-020-01655-1 (PMC7376662; doi:10.1186/s12916-020-01655-1)
Supplement: Supplementary file 7 — Additional file 7. Adjusting for the sum of lysophosphatidylcholines with ≤20 carbons. [file 12916_2020_1655_MOESM7_ESM.pdf]

Additional file 7: Adjusting for the sum of lysophosphatidylcholines with ≤20 carbons

Metabolite	Crude model [a]		Adjusted model	
	OR_{crude} (95% CI)	p_{crude} [b]	$OR_{adjusted}$ (95% CI)	$p_{adjusted}$ [b]
**Overall prostate cancer (40-60 years)**				
LPCs Σ(x ≤20)	1.54 (1.11 − 2.14)	0.0106*	-	-
LPC C14:0	1.11 (0.77 − 1.62)	0.5753	0.74 (0.46 − 1.19)	0.2089
LPC C16:0	1.50 (1.09 − 2.07)	0.0122*	1.11 (0.33 − 3.67)	0.8672
LPC C16:1	1.08 (0.84 − 1.38)	0.5675	0.68 (0.47 − 0.99)	0.0441*
LPC C17:0	1.59 (1.21 − 2.08)	0.0007*	1.58 (1.06 − 2.36)	0.0252*
LPC C18:0	1.59 (1.17 − 2.18)	0.0034*	1.66 (0.84 − 3.27)	0.1439
LPC C18:1	1.39 (1.04 − 1.87)	0.0270*	0.92 (0.47 − 1.82)	0.8142
LPC C18:2	1.19 (0.94 − 1.50)	0.1486	0.84 (0.58 − 1.23)	0.3728
LPC C20:3	1.30 (0.99 − 1.70)	0.0587	1.04 (0.72 − 1.50)	0.8357
LPC C20:4	1.43 (1.09 − 1.86)	0.0085*	1.25 (0.84 − 1.85)	0.2743
**Overall prostate cancer (40-50 years)**				
LPC Σ(x ≤20)	1.30 (0.79 − 2.17)	0.3044	-	-
LPC C14:0	0.98 (0.56 − 1.72)	0.9523	0.74 (0.36 − 1.49)	0.3916
LPC C16:0	1.36 (0.83 − 2.22)	0.2252	2.31 (0.34 − 15.5)	0.3900
LPC C16:1	0.91 (0.63 − 1.32)	0.6318	0.61 (0.35 − 1.05)	0.0738
LPC C17:0	1.10 (0.73 − 1.66)	0.6567	0.81 (0.42 − 1.59)	0.5481
LPC C18:0	1.31 (0.81 − 2.12)	0.2791	1.22 (0.44 − 3.37)	0.7062
LPC C18:1	1.22 (0.78 − 1.90)	0.3924	0.91 (0.31 − 2.61)	0.8535
LPC C18:2	1.05 (0.74 − 1.50)	0.7705	0.77 (0.43 − 1.38)	0.3819
LPC C20:3	1.18 (0.77 − 1.82)	0.4484	1.03 (0.58 − 1.83)	0.9127
LPC C20:4	1.25 (0.83 − 1.86)	0.2854	1.15 (0.62 − 2.13)	0.6557
**Overall prostate cancer (60 years)**				
LPC Σ(x ≤20)	1.74 (1.12 − 2.69)	0.0133*	-	-
LPC C14:0	1.23 (0.74 − 2.05)	0.4182	0.74 (0.39 − 1.42)	0.3703
LPC C16:0	1.62 (1.06 − 2.45)	0.0243*	0.67 (0.14 − 3.14)	0.6102
LPC C16:1	1.23 (0.88 − 1.73)	0.2244	0.76 (0.45 − 1.28)	0.2955
LPC C17:0	2.08 (1.45 − 2.98)	<0.0001*	2.33 (1.39 − 3.9)	0.0013*
LPC C18:0	1.83 (1.22 − 2.75)	0.0037*	2.08 (0.83 − 5.2)	0.1163
LPC C18:1	1.54 (1.05 − 2.28)	0.0293*	0.94 (0.39 − 2.3)	0.8992
LPC C18:2	1.31 (0.96 − 1.80)	0.0943	0.91 (0.56 − 1.49)	0.7099
LPC C20:3	1.38 (0.97 − 1.95)	0.0699	1.03 (0.64 − 1.67)	0.9033
LPC C20:4	1.58 (1.11 − 2.24)	0.0109*	1.33 (0.79 − 2.23)	0.2821
**Non-aggressive prostate cancer (40-60 years)**				
LPC Σ(x ≤20)	1.41 (0.97 − 2.05)	0.0732	-	-
LPC C14:0	0.95 (0.63 − 1.44)	0.8058	0.63 (0.37 − 1.07)	0.0876
LPC C16:0	1.39 (0.97 − 1.99)	0.0764	1.13 (0.30 − 4.29)	0.8605
LPC C16:1	0.97 (0.74 − 1.28)	0.8474	0.61 (0.40 − 0.93)	0.0220*
LPC C17:0	1.36 (1.01 − 1.85)	0.0454*	1.26 (0.80 − 1.98)	0.3126
LPC C18:0	1.49 (1.05 − 2.12)	0.0256*	1.73 (0.81 − 3.70)	0.1592
LPC C18:1	1.35 (0.97 − 1.87)	0.0787	1.14 (0.53 − 2.45)	0.7413
LPC C18:2	1.13 (0.87 − 1.47)	0.3486	0.86 (0.57 − 1.30)	0.4819
LPC C20:3	1.15 (0.85 − 1.56)	0.3803	0.90 (0.59 − 1.37)	0.6333
LPC C20:4	1.28 (0.95 − 1.74)	0.1095	1.10 (0.71 − 1.72)	0.6718
**Non-aggressive prostate cancer (40 – 50 years)**				
LPC Σ(x ≤20)	1.26 (0.73 − 2.17)	0.4117	-	-
LPC C14:0	0.90 (0.50 − 1.63)	0.7345	0.66 (0.31 − 1.41)	0.2845
LPC C16:0	1.32 (0.77 − 2.26)	0.3073	2.55 (0.32 − 20.3)	0.3754
LPC C16:1	0.87 (0.59 − 1.27)	0.4668	0.55 (0.31 − 0.99)	0.0463*
LPC C17:0	1.03 (0.67 − 1.60)	0.8951	0.74 (0.37 − 1.51)	0.4102
LPC C18:0	1.24 (0.73 − 2.09)	0.4280	1.09 (0.36 − 3.27)	0.8827
LPC C18:1	1.21 (0.75 − 1.94)	0.4349	1.05 (0.33 − 3.31)	0.9371
LPC C18:2	1.02 (0.70 − 1.50)	0.9020	0.76 (0.40 − 1.42)	0.3822
LPC C20:3	1.14 (0.72 − 1.81)	0.5722	1.01 (0.55 − 1.86)	0.9722
LPC C20:4	1.24 (0.81 − 1.92)	0.3256	1.20 (0.63 − 2.31)	0.5772

Supplementary Table S4: (Continued)

Metabolite	Crude model [a]<br>OR_{crude} (95% CI)	p_{crude} [b]	Adjusted model<br>$OR_{adjusted}$ (95% CI)	$p_{adjusted}$ [b]
**Non-aggressive prostate cancer (60 years)**				
LPC Σ(x ≤20)	1.55 (0.93 − 2.60)	0.0926	-	-
LPC C14:0	1.00 (0.55 − 1.80)	0.9931	0.60 (0.28 − 1.28)	0.1850
LPC C16:0	1.44 (0.89 − 2.34)	0.1426	0.61 (0.10 − 3.54)	0.5801
LPC C16:1	1.10 (0.74 − 1.65)	0.6290	0.69 (0.38 − 1.27)	0.2373
LPC C17:0	1.77 (1.15 − 2.72)	0.0089*	1.87 (1.02 − 3.40)	0.0417*
LPC C18:0	1.75 (1.08 − 2.81)	0.0225*	2.60 (0.90 − 7.55)	0.0782
LPC C18:1	1.49 (0.94 − 2.37)	0.0909	1.25 (0.45 − 3.50)	0.6746
LPC C18:2	1.24 (0.87 − 1.79)	0.2397	0.96 (0.56 − 1.67)	0.8909
LPC C20:3	1.15 (0.76 − 1.73)	0.5016	0.81 (0.45 − 1.44)	0.4632
LPC C20:4	1.32 (0.86 − 2.02)	0.2017	1.03 (0.56 − 1.89)	0.9248
**Aggressive prostate cancer (40-60 years)**				
LPCs Σ(x ≤20)	2.11 (1.03 − 4.32)	0.0409*	-	-
LPC C14:0	2.29 (0.93 − 5.61)	0.0714	1.53 (0.51 − 4.57)	0.4439
LPC C16:0	2.01 (1.01 − 3.99)	0.0475*	1.01 (0.07 − 15.1)	0.9960
LPC C16:1	1.62 (0.92 − 2.86)	0.0970	1.09 (0.46 − 2.53)	0.8511
LPC C17:0	2.67 (1.48 − 4.83)	0.0011*	3.59 (1.43 − 9.03)	0.0065*
LPC C18:0	2.01 (1.02 − 3.94)	0.0426*	1.41 (0.31 − 6.31)	0.6572
LPC C18:1	1.57 (0.84 − 2.95)	0.1611	0.42 (0.09 − 1.88)	0.2558
LPC C18:2	1.44 (0.85 − 2.46)	0.1780	0.76 (0.31 − 1.91)	0.5613
LPC C20:3	2.05 (1.12 − 3.75)	0.0206*	1.72 (0.77 − 3.80)	0.1844
LPC C20:4	1.99 (1.15 − 3.44)	0.0142*	1.89 (0.78 − 4.56)	0.1580
**Aggressive prostate cancer (40-50 years)**				
LPC Σ(x ≤20)	1.63 (0.42 − 6.39)	0.4817	-	-
LPC C14:0	2.16 (0.35 − 13.3)	0.4068	1.80 (0.21 − 15.4)	0.5903
LPC C16:0	1.58 (0.44 − 5.71)	0.4850	1.19 (0.01 − 164)	0.9439
LPC C16:1	1.64 (0.45 − 6.02)	0.4532	1.40 (0.27 − 7.37)	0.6905
LPC C17:0	1.77 (0.53 − 5.94)	0.3579	1.99 (0.22 − 18.1)	0.5423
LPC C18:0	1.83 (0.50 − 6.74)	0.3659	2.35 (0.16 − 35.0)	0.5362
LPC C18:1	1.27 (0.34 − 4.79)	0.7216	0.42 (0.02 − 7.50)	0.5557
LPC C18:2	1.25 (0.49 − 3.17)	0.6413	0.87 (0.18 − 4.20)	0.8567
LPC C20:3	1.53 (0.43 − 5.48)	0.5157	1.24 (0.23 − 6.72)	0.8036
LPC C20:4	1.26 (0.43 − 3.65)	0.6755	0.77 (0.12 − 4.87)	0.7842
**Aggressive prostate cancer (60 years)**				
LPC Σ(x ≤20)	2.32 (1.00 − 5.38)	0.0500	-	-
LPC C14:0	2.33 (0.83 − 6.54)	0.1091	1.43 (0.40 − 5.12)	0.5839
LPC C16:0	2.20 (0.97 − 4.97)	0.0579	0.97 (0.04 − 24.8)	0.9858
LPC C16:1	1.61 (0.86 − 3.04)	0.1387	0.94 (0.34 − 2.60)	0.9063
LPC C17:0	3.02 (1.52 − 6.01)	0.0016*	4.06 (1.46 − 11.3)	0.0075*
LPC C18:0	2.08 (0.95 − 4.56)	0.0684	1.10 (0.18 − 6.72)	0.9191
LPC C18:1	1.67 (0.81 − 3.41)	0.1624	0.38 (0.06 − 2.29)	0.2915
LPC C18:2	1.54 (0.81 − 2.95)	0.1890	0.73 (0.24 − 2.26)	0.5842
LPC C20:3	2.21 (1.11 − 4.40)	0.0233*	1.85 (0.75 − 4.56)	0.1793
LPC C20:4	2.33 (1.22 − 4.45)	0.0105*	2.45 (0.89 − 6.76)	0.0826

[a] The results for the individual LPCs (crude models) are also presented in Additional file 3.

[b] Metabolites with nominal p-value <0.05 are marked with (*).
