# Supplementary material for: Identification of metabolites associated with prostate cancer risk: a nested case-control study with long follow-up in the Northern Sweden Health and Disease Study
Source: BMC Med. 2020 Jul 23;18:187. doi: 10.1186/s12916-020-01655-1 (PMC7376662; doi:10.1186/s12916-020-01655-1)
Supplement: Supplementary file 8 — Additional file 8. Correlation of lysophosphatidylcholines with dietary fatty acids. [file 12916_2020_1655_MOESM8_ESM.pdf]

Additional file 8: Correlation of lysophosphatidylcholines with dietary fatty acids

**Cx:y** [a]	**Correlation**	**p-value**
C14:0	0.10	<0.0001
C16:0	-0.02	0.5020
C17:0	0.19	<0.0001
C18:2	-0.02	0.4552
C20:4	0.04	0.1020

[a] The spearman correlation coefficients (and p-values) were derived based on values in case-controls with a full dietary fatty acid profile (n = 1524).
