# Supplementary material for: Identification of metabolites associated with prostate cancer risk: a nested case-control study with long follow-up in the Northern Sweden Health and Disease Study
Source: BMC Med. 2020 Jul 23;18:187. doi: 10.1186/s12916-020-01655-1 (PMC7376662; doi:10.1186/s12916-020-01655-1)
Supplement: Supplementary file 9 — Additional file 9. Status of glucose metabolism and prostate cancer risk. [file 12916_2020_1655_MOESM9_ESM.pdf]

Additional file 9: Status of glucose metabolism and prostate cancer risk

	NGT [a]	IGT [a]	IFG [a]	T2D [a, b]	Missing
**Overall prostate cancer (40-60 years)** [c]					
Cases (N)	580	93	70	31	3
Controls (N)	541	138	67	27	4
OR (95% CI)	1.00 (referent)	0.63 (0.47 − 0.85)	0.95 (0.65 − 1.37)	1.02 (0.59 – 1.76)	
p-value [d]	−	0.0024*	0.7659	0.9443	
**Overall prostate cancer (40-50 years)** [c]					
Cases (N)	272	23	27	10	1
Controls (N)	255	46	26	5	1
OR (95% CI)	1.00 (referent)	0.44 (0.25 − 0.77)	0.90 (0.49 – 1.67)	1.91 (0.65 – 5.61)	
p-value [d]	−	0.0039*	0.7443	0.2399	
**Overall prostate cancer (60 years)** [c]					
Cases (N)	308	70	43	21	2
Controls (N)	286	92	41	22	3
OR (95% CI)	1.00 (referent)	0.73 (0.51 – 1.04)	0.97 (0.61 – 1.54)	0.83 (0.61 – 1.58)	
p-value [d]	−	0.0781	0.8891	0.5651	
**Non-aggressive prostate cancer (40-60 years)** [c]					
Cases (N)	453	77	52	24	2
Controls (N)	434	108	46	18	2
OR (95% CI)	1.00 (referent)	0.69 (0.49 – 0.95)	1.04 (0.67 – 1.59)	1.29 (0.67 – 2.46)	
p-value [d]	−	0.0248*	0.8737	0.4480	
**Non-aggressive prostate cancer (40-50 years)** [c]					
Cases (N)	234	21	24	9	1
Controls (N)	224	41	21	2	1
OR (95% CI)	1.00 (referent)	0.44 (0.24 – 0.81)	0.97 (0.52 – 1.90)	4.5 (0.97 – 20.8)	
p-value [d]	−	0.0077*	0.9899	0.0544	
**Non-aggressive prostate cancer (60 years)** [c]					
Cases (N)	219	56	28	15	1
Controls (N)	210	67	25	16	1
OR (95% CI)	1.00 (referent)	0.82 (0.55 – 1.23)	1.06 (0.60 – 1.89)	0.88 (0.41 – 1.89)	
p-value [d]	−	0.3415	0.8414	0.7480	
**Aggressive prostate cancer (40-60 years)** [c]					
Cases (N)	127	16	18	7	1
Controls (N)	107	30	21	9	2
OR (95% CI)	1.00 (referent)	0.47 (0.24 – 0.91)	0.72 (0.34 – 1.51)	0.53 (0.18 – 1.53)	
p-value [d]	−	0.0256*	0.3789	0.2410	
**Aggressive prostate cancer (40-50 years)** [c]					
Cases (N)	38	2	3	1	0
Controls (N)	31	5	5	3	0
OR (95% CI)	1.00 (referent)	0.31 (0.05 – 1.81)	0.33 (0.04 – 3.21)	0.24 (0.02 – 2.61)	
p-value [d]	−	0.1939	0.3414	0.2386	
**Aggressive prostate cancer (60 years)** [c]					
Cases (N)	89	14	15	6	1
Controls (N)	76	25	16	6	2
OR (95% CI)	1.00 (referent)	0.51 (0.25 – 1.06)	0.82 (0.37 – 1.82)	0.68 (0.20 – 2.31)	
p-value [d]	−	0.0709	0.6228	0.5371	

[a] NGT if glucose (0h) <6.1 mmol/L, IGT if glucose (0h) <7.0 and (2h) <7.8 mmol/L, IFG if glucose (0h) ≥6.1, <7.0mmol/L, T2D if glucose (0h) ≥7.0 or (2h) ≥11.1 mmol/L.

[b] Individuals with diagnosed diabetes when enrolled to the NSHDS were not included in the present study.

[c] **Stratifications:** overall prostate cancer (40-60 years, n = 770 pairs; 40-50 years, n = 331 pairs; 60 years, n = 439 pairs), non-aggressive prostate cancer (40-60 years, n = 604 pairs; 40-50 years, n = 287; 60 years, n = 317 pairs), aggressive prostate cancer (40-60 years, n = 166 pairs; 40-50 years, n = 44 pairs; 60 years, n = 122 pairs). Note that the small sample size in some subgroups yields uncertain risk estimates.

[d] p-values <0.05 are indicated with (*).
