# Supplementary material for: Identification of metabolites associated with prostate cancer risk: a nested case-control study with long follow-up in the Northern Sweden Health and Disease Study
Source: BMC Med. 2020 Jul 23;18:187. doi: 10.1186/s12916-020-01655-1 (PMC7376662; doi:10.1186/s12916-020-01655-1)
Supplement: Supplementary file 10 — Additional file 10. Prostate cancer risk in case-controls with normal glucose tolerance. [file 12916_2020_1655_MOESM10_ESM.pdf]

Additional file 10: Prostate cancer risk in case-controls with normal glucose tolerance

Metabolite	OR_{NGT} (95% CI)	p_{NGT} [a]	ΔOR [b]	Δp [b]
**Overall prostate cancer (40-60 years)** [c]				
LPC C17:0	1.91 (1.28 – 2.86)	0.0016*	+0.33	+0.001
LPC C18:0	2.22 (1.39 – 3.53)	0.0008*	+0.63	-0.003
Glycine	1.29 (0.78 – 2.13)	0.3167	+0.12	-0.078
Pyruvate	0.96 (0.73 – 1.25)	0.7638	+0.17	+0.745
PC ae C38:3	1.93 (1.17 – 3.18)	0.0098*	+0.59	-0.067
PC ae C38:4	1.85 (1.10 – 3.09)	0.0198*	+0.38	-0.011
LPC C20:3	1.62 (1.09 – 2.42)	0.0176*	+0.33	-0.041
LPC C20:4	1.60 (1.08 – 2.37)	0.0196*	+0.17	+0.011
PC ae C40:2	1.66 (1.06 – 2.60)	0.0282*	+0.33	-0.039
C18:2	0.89 (0.63 – 1.25)	0.4891	±0.00	+0.181
**Overall prostate cancer (40-50 years)** [c]				
LPC C17:0	1.76 (0.92 – 3.44)	0.0888	+0.68	-0.568
LPC C18:0	2.26 (1.07 – 4.78)	0.0336*	+0.95	-0.246
Glycine	2.22 (1.11 – 4.46)	0.0251*	+0.11	+0.017
Pyruvate	0.84 (0.58 – 1.22)	0.3662	+0.19	+0.360
PC ae C38:3	1.31 (0.60 – 2.84)	0.4999	+0.13	-0.013
PC ae C38:4	1.50 (0.66 – 3.40)	0.3374	+0.09	+0.126
LPC C20:3	1.34 (0.71 – 2.55)	0.3669	+0.16	-0.082
LPC C20:4	1.72 (0.92 – 3.21)	0.0875	+0.48	-0.198
PC ae C40:2	1.74 (0.86 – 3.52)	0.1206	+0.60	-0.462
C18:2	0.93 (0.56 – 1.52)	0.7618	+0.03	+0.201
**Overall prostate cancer (60 years)** [c]				
LPC C17:0	3.14 (1.72 – 5.73)	0.0002*	+1.07	±0.000
LPC C18:0	3.19 (1.60 – 6.36)	0.0010*	+1.36	-0.003
Glycine	0.58 (0.26 – 1.32)	0.1949	-0.10	+0.048
Pyruvate	1.14 (0.75 – 1.72)	0.5494	+0.20	-0.102
PC ae C38:3	3.28 (1.54 – 6.98)	0.0020*	+1.80	-0.074
PC ae C38:4	2.73 (1.30 – 5.72)	0.0079*	+1.22	-0.067
LPC C20:3	1.94 (1.11 – 3.39)	0.0203*	+0.56	-0.050
LPC C20:4	1.84 (1.04 – 3.26)	0.0349*	+0.27	+0.024
PC ae C40:2	2.20 ()1.13 – 4.27)	0.0199*	+0.71	-0.034
C18:2	0.92 (0.55 – 1.53)	0.7454	+0.05	+0.348
**Non-aggressive prostate cancer (40-60 years)** [c]				
LPC C17:0	1.79 (1.14 – 2.81)	0.0114*	+0.43	-0.034
LPC C18:0	2.12 (1.26 – 3.59)	0.0048*	+0.63	-0.021
Glycine	1.52 (0.86 – 2.70)	0.1504	+0.41	-0.709
Pyruvate	0.87 (0.65 – 1.17)	0.3526	+0.11	+0.335
PC ae C38:3	1.56 (0.90 – 2.70)	0.1121	+0.47	-0.531
PC ae C38:4	1.50 (0.84 – 2.67)	0.1745	+0.26	-0.116
LPC C20:3	1.41 (0.89 – 2.21)	0.1425	+0.26	-0.238
LPC C20:4	1.46 (0.93 – 2.29)	0.1015	+0.18	-0.008
PC ae C40:2	1.52 (0.91 – 2.54)	0.1095	+0.40	-0.398
C18:2	1.01 (0.69 – 1.47)	0.9718	-0.01	+0.052
**Non-aggressive prostate cancer (40-50 years)** [c]				
LPC C17:0	1.20 (0.66 – 2.19)	0.5567	+0.17	-0.338
LPC C18:0	1.63 (0.81 – 3.30)	0.1721	+0.40	-0.256
Glycine	2.22 (1.07 – 4.61)	0.0321*	+0.21	+0.014
Pyruvate	0.80 (0.54 – 1.20)	0.2796	+0.16	+0.272
PC ae C38:3	0.96 (0.48 – 1.97)	0.9133	-0.07	-0.004
PC ae C38:4	1.09 (0.49 – 2.40)	0.8372	-0.24	+0.500
LPC C20:3	1.21 (0.65 – 2.27)	0.5512	+0.07	-0.021
LPC C20:4	1.43 (0.79 – 2.59)	0.2421	+0.18	-0.084
PC ae C40:2	1.19 (0.62 – 2.29)	0.6019	+0.15	-0.264
C18:2	0.96 (0.59 – 1.55)	0.8569	-0.02	-0.042

Additional file 10: (Continued)

Metabolite	OR_{NGT} (95% CI)	p_{NGT} [a]	ΔOR [b]	Δp [b]
**Non-aggressive prostate cancer (60 years)** [c]				
LPC C17:0	2.92 (1.45 – 5.88)	0.0027*	+1.15	-0.006
LPC C18:0	2.90 (1.32 – 6.38)	0.0081*	+1.16	-0.014
Glycine	0.66 (0.23 – 1.90)	0.4420	-0.11	+0.015
Pyruvate	0.97 (0.62 – 1.54)	0.9102	+0.04	+0.216
PC ae C38:3	3.07 (1.26 – 7.49)	0.0136*	+1.92	-0.573
PC ae C38:4	2.16 (0.91 – 5.12)	0.0821	+0.99	-0.497
LPC C20:3	1.65 (0.85 – 3.20)	0.1367	+0.50	-0.365
LPC C20:4	1.50 (0.75 – 2.99)	0.2497	+0.18	+0.048
PC ae C40:2	2.22 (0.96 – 5.14)	0.0618	+1.02	-0.384
C18:2	1.09 (0.60 – 2.00)	0.7775	+0.04	-0.010
**Aggressive prostate cancer (40-60 years)** [c]				
LPC C17:0	2.45 (1.00 – 6.06)	0.0511	-0.21	+0.050
LPC C18:0	2.61 (0.94 – 7.24)	0.0661	+0.60	+0.024
Glycine	0.71 (0.24 – 2.11)	0.5384	-0.06	+0.021
Pyruvate	1.90 (0.86 – 4.22)	0.1124	+1.00	-0.559
PC ae C38:3	4.98 (1.43 – 17.4)	0.0119*	+1.69	+0.009
PC ae C38:4	4.01 (1.24 – 17.4)	0.0202*	+1.32	+0.009
LPC C20:3	2.70 (1.09 – 6.66)	0.0314*	+0.65	+0.011
LPC C20:4	2.15 (0.94 – 4.89)	0.0688	+0.16	+0.055
PC ae C40:2	2.20 (0.85 – 5.69)	0.1039	-0.29	+0.094
C18:2	0.47 (0.20 – 1.13)	0.0919	-0.04	+0.075
**Aggressive prostate cancer (40-50 years)** [c]				
LPC C17:0	1.02 (0.21 – 4.79)	0.9880	-0.75	+0.630
LPC C18:0	1.44 (0.34 – 6.13)	0.6200	-0.38	+0.254
Glycine	2.20 (0.21 – 23.6)	0.5133	-1.62	+0.300
Pyruvate	1.52 (0.38 – 6.08)	0.5547	+0.73	-0.073
PC ae C38:3	10.5 (0.75 – 147)	0.0809	+6.33	-0.020
PC ae C38:4	2.60 (0.35 – 19.4)	0.3509	+0.57	+0.009
LPC C20:3	2.36 (0.46 – 12.1)	0.3016	+0.85	-0.214
LPC C20:4	1.24 (0.21 – 5.01)	0.7657	-0.02	+0.090
PC ae C40:2	2.35 (0.33 – 16.8)	0.3964	-0.82	+0.206
C18:2	0.18 (0.02 – 1.50)	0.1138	-0.25	-0.052
**Aggressive prostate cancer (60 years)** [c]				
LPC C17:0	3.82 (1.18 – 12.4)	0.0258*	+0.80	+0.024
LPC C18:0	4.28 (1.02 – 18.0)	0.0467*	+2.21	-0.022
Glycine	0.48 (0.13 – 1.77)	0.2724	-0.05	+0.104
Pyruvate	2.12 (0.78 – 5.65)	0.1322	+1.18	-0.691
PC ae C38:3	3.85 (0.93 – 16.0)	0.0629	+0.78	+0.050
PC ae C38:4	4.94 (1.15 – 21.2)	0.0313*	+1.98	+0.014
LPC C20:3	2.85 (0.97 – 8.40)	0.0574	+0.64	+0.034
LPC C20:4	1.60 (1.08 – 2.37)	0.0196*	+0.17	+0.011
PC ae C40:2	2.16 (0.73 – 6.38)	0.1645	-0.22	+0.141
C18:2	0.61 (0.23 – 1.59)	0.3078	+0.07	+0.259

[a] p-values <0.05 are indicated with (*).

[b] ΔOR= (OR_{NGT} - OR_{crude}) and Δp = (p_{NGT} - p_{crude}), where each corresponding OR_{crude} and p_{crude} is listed in Additional file 3.

[c] **Stratifications:** Overall prostate cancer in subjects aged 40-60 years (387/370 pairs, MS/NMR), 40-50 years (198/193, MS/NMR) and 60 years, 189/177 pairs, MS/NMR). Non-aggressive prostate cancer in subjects aged 40-60 years (311/298 pairs, MS/NMR), 40-50 years (173/168 pairs, MS/NMR) and 60 years (138/130 pairs, MS/NMR). Aggressive prostate cancer amongst subjects aged: 40-60 years (76/72 pairs, MS/NMR)**,** 40-50 years (25/25, MS/NMR) and 60 years (51/47 pairs, MS/NMR). Note that the small sample size in some subgroups yields uncertain risk estimates.
